# SFPQ and Tau: critical factors contributing to rapid progression of Alzheimer’s disease

**DOI:** 10.1007/s00401-020-02178-y

**Published:** 2020-06-23

**Authors:** Neelam Younas, Saima Zafar, Mohsin Shafiq, Aneeqa Noor, Anna Siegert, Amandeep Singh Arora, Alexey Galkin, Ayesha Zafar, Mathias Schmitz, Christine Stadelmann, Olivier Andreoletti, Isidre Ferrer, Inga Zerr

**Affiliations:** 1 Department of Neurology, University Medical Center Göttingen and the German Center for Neurodegenerative Diseases (DZNE), Robert-Koch-Straße 40, 37075 Göttingen, Germany; 2 Biomedical Engineering and Sciences Department, School of Mechanical and Manufacturing Engineering (SMME), National University of Sciences and Technology (NUST), Islamabad, Pakistan; 3 Institute of Neuropathology, University Medical Center Hamburg-Eppendorf, Martinistraße 52, 20246 Hamburg, Germany; 4 Institute for Behavioral Medicine Research, The Ohio State University, 460 Medical Center Dr, Columbus, OH 43210 USA; 5 St. Petersburg Branch, Vavilov Institute of General Genetics, St. Petersburg, Russia; 6 College of Medicine Center for Pharmacogenomics, The Ohio State University, 460 W 12th Avenue, Columbus, OH 1004 BRT USA; 7 Institute of Neuropathology, University Medical Center, Göttingen, Germany; 8 UMR INRA ENVT 1225- Interactions Hôte Agent Pathogène–École Nationale Vétérinaire de Toulouse, Toulouse, France; 9 Department of Pathology and Experimental Therapeutics, University of Barcelona, Barcelona, Spain; 10 Bellvitge University Hospital-IDIBELL, Barcelona, Spain; 11 CIBERNED, Barcelona, Spain; 12 Hospitalet de Llobregat, Barcelona, Spain

**Keywords:** RNA-binding proteins, Rapidly progressive Alzheimer’s disease, SFPQ, Stress granules, Dislocation, 3xTg mice

## Abstract

Dysfunctional RNA-binding proteins (RBPs) have been implicated in several neurodegenerative disorders. Recently, this paradigm of RBPs has been extended to pathophysiology of Alzheimer’s disease (AD). Here, we identified disease subtype specific variations in the RNA-binding proteome (RBPome) of sporadic AD (spAD), rapidly progressive AD (rpAD), and sporadic Creutzfeldt Jakob disease (sCJD), as well as control cases using RNA pull-down assay in combination with proteomics. We show that one of these identified proteins, splicing factor proline and glutamine rich (SFPQ), is downregulated in the post-mortem brains of rapidly progressive AD patients, sCJD patients and 3xTg mice brain at terminal stage of the disease. In contrast, the expression of SFPQ was elevated at early stage of the disease in the 3xTg mice, and in vitro after oxidative stress stimuli. Strikingly, in rpAD patients’ brains SFPQ showed a significant dislocation from the nucleus and cytoplasmic colocalization with TIA-1. Furthermore, in rpAD brain lesions, SFPQ and p-tau showed extranuclear colocalization. Of note, association between SFPQ and tau-oligomers in rpAD brains suggests a possible role of SFPQ in oligomerization and subsequent misfolding of tau protein. In line with the findings from the human brain, our in vitro study showed that SFPQ is recruited into TIA-1-positive stress granules (SGs) after oxidative stress induction, and colocalizes with tau/p-tau in these granules, providing a possible mechanism of SFPQ dislocation through pathological SGs. Furthermore, the expression of human tau in vitro induced significant downregulation of SFPQ, suggesting a causal role of tau in the downregulation of SFPQ. The findings from the current study indicate that the dysregulation and dislocation of SFPQ, the subsequent DNA-related anomalies and aberrant dynamics of SGs in association with pathological tau represents a critical pathway which contributes to rapid progression of AD.

**Electronic supplementary material:**

The online version of this article (10.1007/s00401-020-02178-y) contains supplementary material, which is available to authorized users.

## Introduction

Alzheimer’s disease (AD) is the most prevalent form of dementia with progressive neurodegeneration, affecting more than 40 million people worldwide [57, 61]. Typically, sporadic AD cases are characterized by a slow progression in cognitive decline and a disease duration of ~ 8 years after the onset of clinical symptoms [66]. However, mounting evidence show variability in both clinical phenotypes and progression rates [1, 6, 21, 68]. Recently, a rapidly progressive form of AD (rpAD) has been identified with a steep decline in Mini-Mental State Examination (MMSE: a psychometric test) score (e.g. ≥ 5 points/year), and/or a reduced disease duration (~ 4 years) [47, 54, 67, 74, 82]. The clinical definition of rpAD differs greatly across various studies in the literature. Preliminary evidence provide a compelling view that rpAD is associated with a distinct molecular and pathogenic cascade [6, 21, 26]. However, no significant differences have been detected in the core neuropathological features between spAD and rpAD [21, 68], suggesting a great demand for understanding of underlying pathogenic mechanisms responsible for this heterogeneity. Rapidly progressive AD cases exhibit a partial clinical overlap with sporadic CJD, another rapidly progressive dementia, which makes early differential diagnosis a challenge [1, 68, 75]. The identification of the common pathogenic mechanisms and molecular factors that lead to faster progression, may be of common therapeutic interests [83].

Extracellular Aβ plaques and intracellular neurofibrillary tangles (NFTs) of hyperphosphorylated tau protein are two cardinal features of AD. The amyloid hypothesis proposes Aβ pathology as the principal pathological feature of the disease [32] that triggers a cascade of additional pathological events, including the formation of NFTs, neuroinflammation, oxidative stress and neuronal loss [32, 62, 78]. Recent evidence suggests a novel pathological feature of tau, in relation to cytoplasmic stress granules (SGs) and RNA-binding proteins (RBPs), through which tau disrupts cellular homeostasis. RBPs, like TIA-1, co-localize with hyperphosphorylated-tau and aggravate tau pathology [85, 86]. SGs are reversible cytoplasmic aggregates composed of RBPs, RNA and stalled translation initiation complexes that usually resolubilize after removal of stress [3, 38]. The association of several RBPs with AD and other neurological diseases redirects research efforts toward understanding the role of RBPs in neurodegeneration and progression of these diseases. To this end, this study aimed to identify and characterize RNA-binding proteome (RBPome) variations in subtypes of Alzheimer’s and prion disease (sCJD), to find out potential candidates underlying phenotypic heterogeneity and variant progression rates at molecular level.

The proteomic investigation, in combination with several bioinformatic and computational approaches highlighted quantitative and qualitative changes in the identified RBPome, in a disease subtype specific manner. Here, we show that one of these identified proteins- splicing factor proline and glutamine rich (SFPQ), is dysregulated in association with tau and TIA-1 proteins, particularly in rpAD patients.

## Materials and methods

### Ethics statement

Frontal cortex samples from spAD, rpAD and age-matched controls were provided by Institute of Neuropathology Brain Bank (HUB-ICO-IDIBELL Biobank) and Biobank of Hospital Clinic-IDIBAPS Spain, following the legislation (Ley de la Investigación Biomédica 2013 and Real DecretoBiobancos, 2014) and the approval of local ethics committees. Frontal cortex samples from patients with sCJD subtypes (-MM1 & -VV2) were obtained from the Department of Neurology at the University Medical Centre, Göttingen, Germany. Ethics approval was obtained from the Ethical Committees of the University Medical Centre, Göttingen, Germany and all the procedures were conducted in accordance to ethics regulations (Nr. 1/11/93 and Nr. 9/6/08).

All animal experiments were conducted according to the ethical standards of the Regierungspräsidium Tübingen (Regional Council) experimental no. FLI 231/07 file reference number 35/9185.81-2. All procedures were carried out in accordance with the institutional and French national guidelines within the European Community Council Directive 86/609/EEC. The experimental procedures approved by the INRA Toulouse/ENVT ethics committee were used.

### Patient cohorts and rpAD/spAD/sCJD subtype characterization

Patient cohort processing, neuropathological examination and brain tissue collection for this study were all conducted as previously described [90, 91]. The post‐mortem interval was between 3 and 18 h. Both spAD and rpAD samples had AD pathologies with Braak stage ≥ V. The samples for AD subtypes were included without co-pathologies. Likewise, the sCJD subtypes (sCJD-MM1 & -VV2) were exclusively composed of prionopathies. Details of the patient cohorts are described in the supplementary tables (suppl. Tables 4 and 5, online resource). Diagnosis of all the control and spAD cases is described in detail by Hernandez et al. [33]. All the cases were confirmed by neuropathological examination of 25 regions of the cerebral cortex, thalamus, diencephalon, cerebellum and brainstem as described previously [33]. Haematoxylin and eosin, Klüver-Barrera and immunohistochemistry (for glialfibrillary acidic protein, β-amyloid, phosphorylated tau, α-synuclein, TDP 43, ubiquitin, p62 and microglial specific markers) were performed [33]. Neuropathological assessment of spAD was done following the Braak and Braak stages [16–18]. All rpAD cases met the current criteria of rpAD [21]. Braak stages of NFT and plaque pathology: I-VI/0-C, and Thal phases 1–5 [80] of all the cases are described in the supplementary table (suppl. Table 4, online resource). There were no significant differences observed in the age distribution (suppl. Figure 1a, online resource).

### Humanized mice model of combined Aβ and tau pathology (3xTg)

The mouse model (3xTg) was initially generated and characterized by Oddo et al. [58]. Transgenic mice between 3 and 4 months of age were inoculated with10% brain homogenate from AD patient (Case no. 10 in the suppl. Table 4, online resource), in the thalamus. The cortical brain tissue was homogenized in PBS. After centrifugation, supernatant was transferred to new tube and 20 μL of the supernatant was used for inoculation. All 3xTg-mice inoculated (AD) and non-inoculated (controls) were sacrificed and brains were collected at 4 time-points corresponding to 3-, 6-, 9- and 12-months post-inoculation (mpi). All the mice (*n* = 48) were anesthetized and decapitated. The cortical tissues were obtained and immediately stored in liquid nitrogen for further analysis.

### RNA extraction and tissue lysis for pull-down assay

Before RNA extraction, surfaces and lab-ware were cleaned with RNAseZap (Thermo Fischer Scientific). Total RNA was isolated, from the human brain frontal cortices of spAD, rpAD, sCJD and control cases, using RNeasy Plus Universal kits (Qiagen, Germany), including DNAse treatment in accordance with the manufacturer’s instructions. The concentration of RNA was measured using a NanoDrop 2000 (Thermo Scientific). The integrity of isolated RNA was confirmed with the Bioanalyzer (Agilent Technologies, Santa Clara, CA). All the samples bearing RNA integration number (RIN) ≥ 5 were selected for downstream analysis. Frontal cortex tissues from same cases were lysed in ‘’tissue protein extraction reagent’’ (T-PER; Thermo Fischer Scientific), supplemented with phosphatase and protease inhibitor cocktails (Roche, Germany). The concentration of protein extracts was determined using Bradford assay (Bio-Rad, Germany). The concentration of total protein extract was kept greater than 2 mg/mL, such that there is a significant dilution into the binding reaction buffer.

### RNA pull-down assay

**Fig. 1:**
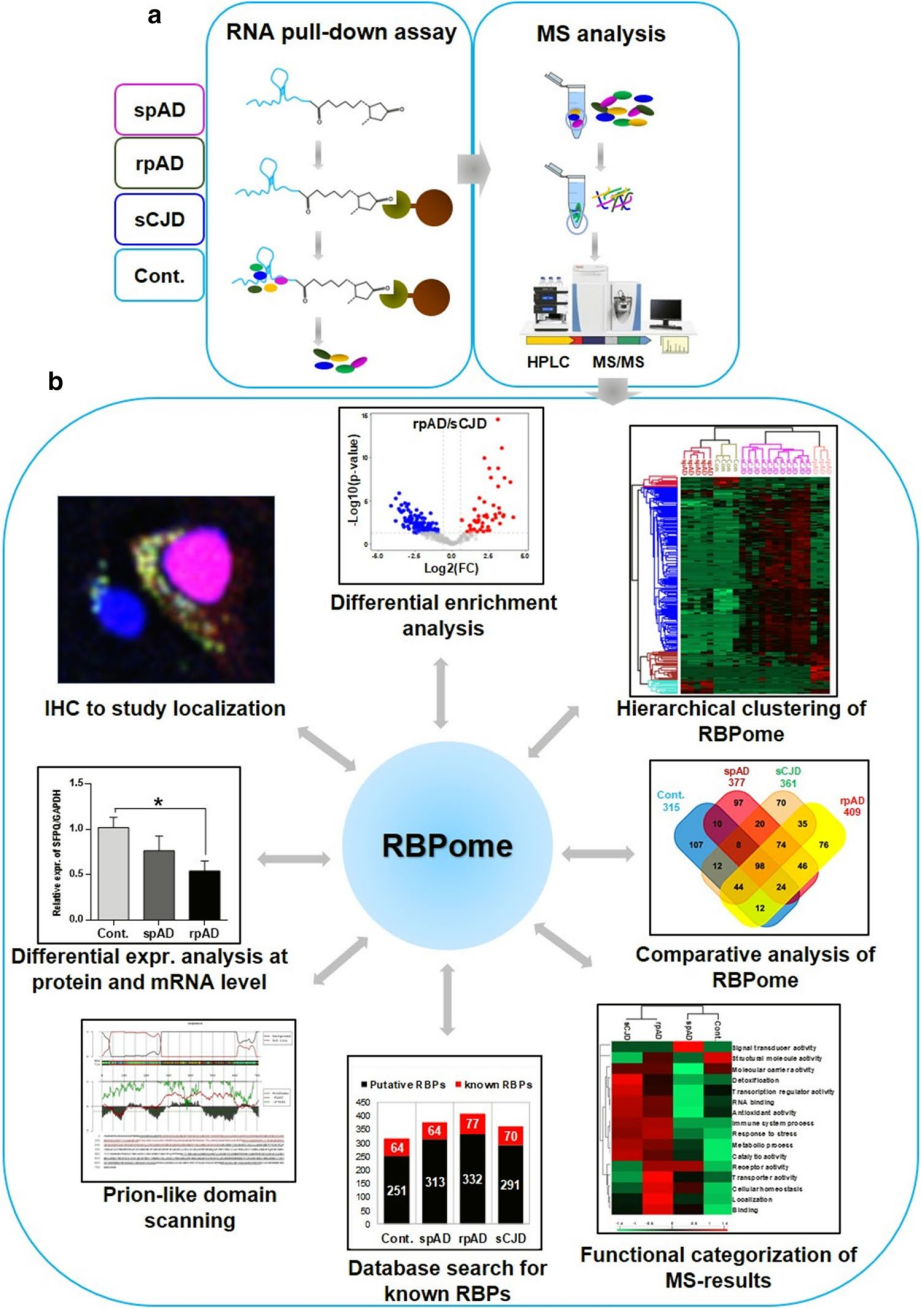
Identification of RNA-binding proteins by RNA pull-down assay and mass spectrometry analysis. **a** Total RNA was isolated from the human brain frontal cortical region of 20 cases (spAD, rpAD, sCJD-MM1, sCJD-VV2 as well as controls). Bead-only control was used for non-specific binding. Isolated protein complexes were identified quantitatively using MS/MS analysis. In total, 1091 proteins were identified and quantified at a minimum peptide count of 2 and a protein threshold of 99%. **b** Target selection from proteomic investigation and their pathological characterization in the post-mortem human brains: a combination of bioinformatic and computational approaches was used to find out significant hits from the proteomic study, including differential enrichment analysis of MS data, hierarchical clustering analysis to visualize global proteome profile, comparative RBPome analysis, Gene Ontology (GO) functional enrichment analysis, database search for identification of bona-fide and novel/putative RBP candidates, and prion-like domain scanning with PLAAC database. Target candidates prioritized from proteomic study were pathologically characterized in the post-mortem human brain, using various techniques including immunoblotting, qRT-PCR and immunohistochemical analysis

Total brain-derived RNA was labelled with desthiobiotin at 3′- end using T4-RNA ligase from Pierce RNA 3′-End Desthiobiotinylation Kit, which were a part of the Pierce™ Magnetic RNA–protein pull-down kit (Thermo Fisher Scientific). The labelling efficiency of experimentally labelled samples (diseased and control) was assessed by dot blotting [(Thermo Scientific Chemiluminescent Detection Module (Product No. 89880)], according to instructions from the manufacturer. Desthiobiotinylated RNA was used for the enrichment of RNA-binding protein complexes, according to the manufacturer’s recommendations (Thermo Fischer Scientific). Isolated protein complexes were eluted with biotin elution buffer and processed for mass spectrometry (MS) analysis (Fig. 1a). In this assay, streptavidin magnetic beads were mixed with protein extracts in the absence of biotinylated transcript as a control for nonspecific binding. A combination of bioinformatic, computational, and biochemical approaches was used to comprehensively analyse the isolated RBPome (Fig. 1b).

### Label-free quantification mass spectrometry (LFQ-MS) analysis

Mass spectrometry analysis was performed as published previously [90]. Briefly, isolated protein complexes were resolved onto 4–20% Bis–Tris gels (NuPAGE Novex Bis–Tris Mini gels, Invitrogen) for a length of ~ 1 cm and stained with Coomassie stain. The bands were sliced into small cubes (1–2 mm^2^). The digestion of proteins into peptides, identification and quantification of these peptides was carried out as described previously. Scaffold (version Scaffold_4.8.4, Proteome Software Inc., Portland/OR, USA) was used to validate MS/MS based peptide and protein identifications. Threshold for peptide identifications was established at greater than 95% confidence. For protein identifications, a minimum of 2 peptide count and a confidence threshold of 99% was used. Protein probabilities were allotted by the Protein Prophet algorithm [55].

### Co-immunofluorescence

Formalin fixed and paraffin embedded human brain cortical sections (5 µm thick) from spAD, rpAD and age matched controls were analysed by co-immunofluorescence using protocols validated previously [42, 86]. Imaging was performed with confocal laser scanning microscope TCS-SPE (Leica, Germany; 543 and 633 nm helium–neon and 488 nm argon excitation wavelengths). All images were separately analysed for colocalization using ImageJ (WCIF plugin) and FIJI (coloc 2) software. Threshold Mander’s overlap coefficients (tM1 & tM2) and Pearson's linear correlation coefficient (rP) were calculated to measure the strength and direction of linear correlations between two fluorescence channels. In addition, intensity correlation analysis (ICA) was also performed [43].

### Cell model for stress induction

Confluent cells (HeLa, SH-SY5Y and HEK-293) were subjected to oxidative stress by adding a pre-warmed culture medium containing sodium arsenite (0.6 mM) for 1 h at 37 °C. After one hour of treatment, the cells were either fixed with 4% paraformaldehyde (PFA) for immunocytochemistry or proceeded for immunoblotting analysis.

### Tau-transfections

Plasmids for human wild type-tau (pRK5-EGFP-tau) and mutated-tau (pRK5-EGFP-tau P301L) were obtained from Addgene. The plasmids were propagated in *E. coli.* DH5alpha strain and were extracted for the transfection assay using the Hispeed Plasmid Midi kit (Qiagen, Germany). Transient transfections were achieved using Lipofectamine 2000 (Invitrogen) reagent according to the instructions of the manufacturer. HeLa cells (2 × 10^5^/well) were plated in 6-well plates and cultured for 24 h in the culture medium. The cells were washed with Opti-MEM I, followed by transfection with 2 μg of DNA/well in the Opti-MEM I medium. The cells were harvested from cultures after 24- and 48-h post-transfection followed by immunoblotting and SWATH-MS (Sequential Windowed Acquisition of All Theoretical Fragment Ion Mass Spectra) analysis (detailed protocol is provided in supplementary Material, online resource).

### Statistical analysis

All the data in the present study was obtained after performing at least three independent experiments. All the results are described as mean ± SEM (standard error of the mean). The densitometry analysis of immunoblots was performed with Image Lab software (version 3.0.1). Statistical tests were applied using GraphPad Prism 6.01. The data from mass spectrometry analysis were analysed using the Perseus software. Hierarchical clustering analysis was also performed with Perseus software. Two-dimensional interaction plots were plotted in R (version 3.4.3), followed by editing using Inkscape (version 0.92). For comparisons between two groups, the student's *t*-tests or Welch’s *t*-tests were used. For comparisons between three or more groups, a one-way ANOVA followed by the Tukey post-hoc analysis was used unless otherwise stated in the text. Statistical significance was considered for a *p*-value < 0.05.

## Results

### Global enrichment profile of RNA-binding protein candidates

**Fig. 2:**
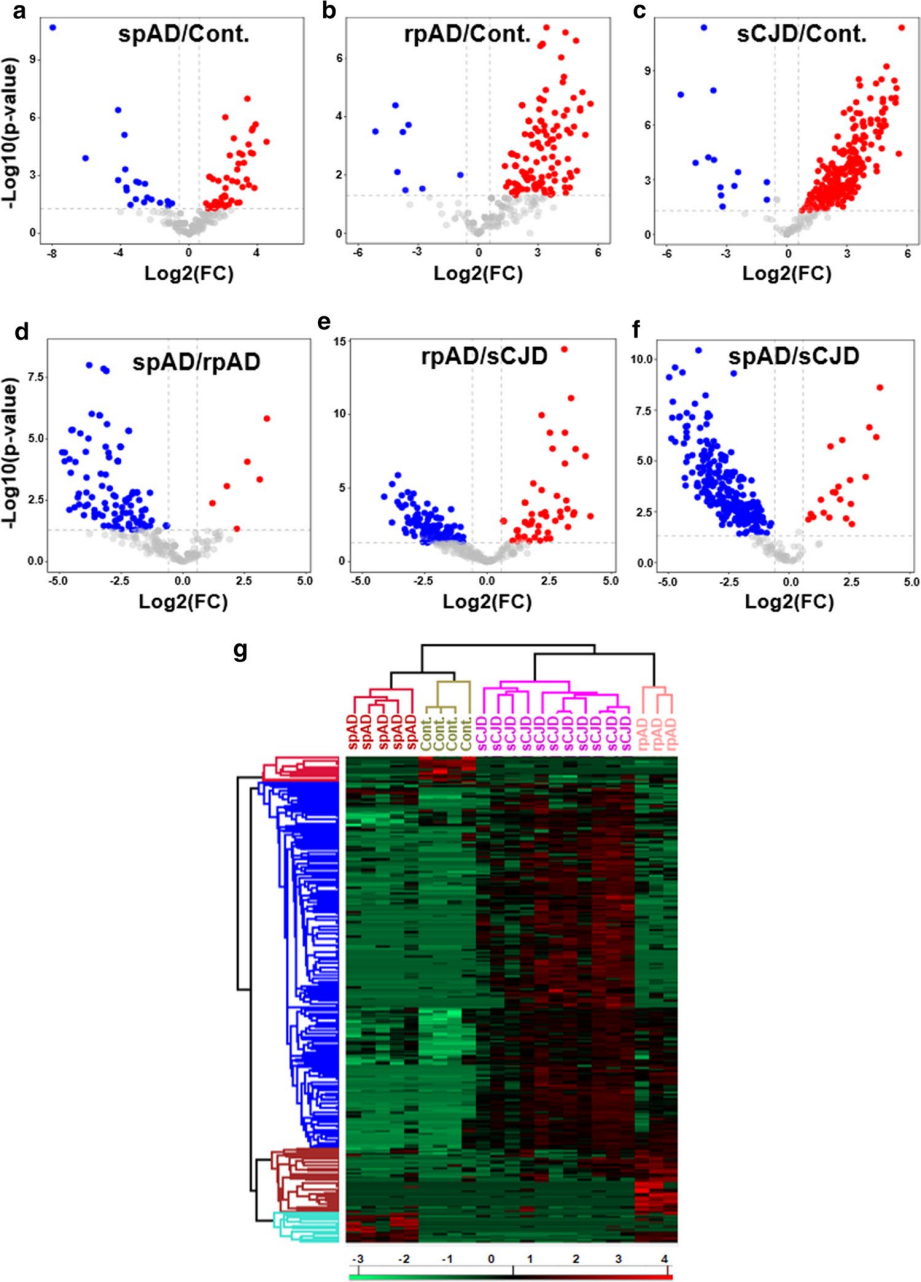
Global enrichment profile of RBPome candidates isolated from the human brain frontal cortical region of 20 cases (spAD, rpAD, sCJD-MM1, sCJD-VV2 as well as controls). **a**–**f** Volcano plots of pairwise comparisons (*t*-tests with BH correction) showing, the -log10-*p*-values (*y*-axis) and the log2(FC) of the proteins that were significantly abundant (*x*-axis) in all the group combinations (spAD vs control, rpAD vs control, sCJD vs control, spAD vs rpAD, rpAD vs sCJD, and spAD vs sCJD). The data points above the dashed lines represent proteins having a *p*-value < 0.05 and FC >  ± 1.5 as significant hits and are depicted in red (for enriched) and blue (for depleted). **g** The heatmap (hierarchical clustering analysis) showing significantly-enriched proteins. The Log2-transformed expression values of proteins were normalized by *Z*-score for each biological replicate. Horizontal-axis indicates the differentially-enriched proteins, and the vertical axis shows the biological replicates from all the groups. Blue denotes depleted proteins, red represents enriched proteins

Emerging evidence demonstrate that numerous neurodegenerative disorders, including AD display RBP pathologies, highlighting a vital role of RBPs in neurodegeneration [23, 52, 86]. In the present study, using brain-derived total RNA based pull-down approach in combination with label free quantitative proteomics, we identified subtype specific variations in the RBPome from the human brain of spAD, rpAD and sCJD. Differential enrichment analysis of RBP-candidates revealed 338 proteins with significant differences (*p* < 0.05) in abundance between any of the experimental groups (Fig. 2a–f, and suppl. Table 11, online resource). Using significantly enriched proteins in the hierarchical clustering analysis, four different expression signatures were identified, which are represented in the form of heatmap (Fig. 2g). Based on RBP-signatures, rpAD and sCJD groups appeared largely similar as they were segregated into a single cluster, whereas both were different from the control group (Fig. 2g). The control and spAD cases were segregated into a separate cluster based on the full set of differentially enriched proteins, which indicated similarities in the protein profiles between control and spAD groups. Overall, clustering of the individual replicates indicated low biological variance and segregation of all the groups, showing the reproducibility and robustness of the workflow (Fig. 2g).

**Fig. 3:**
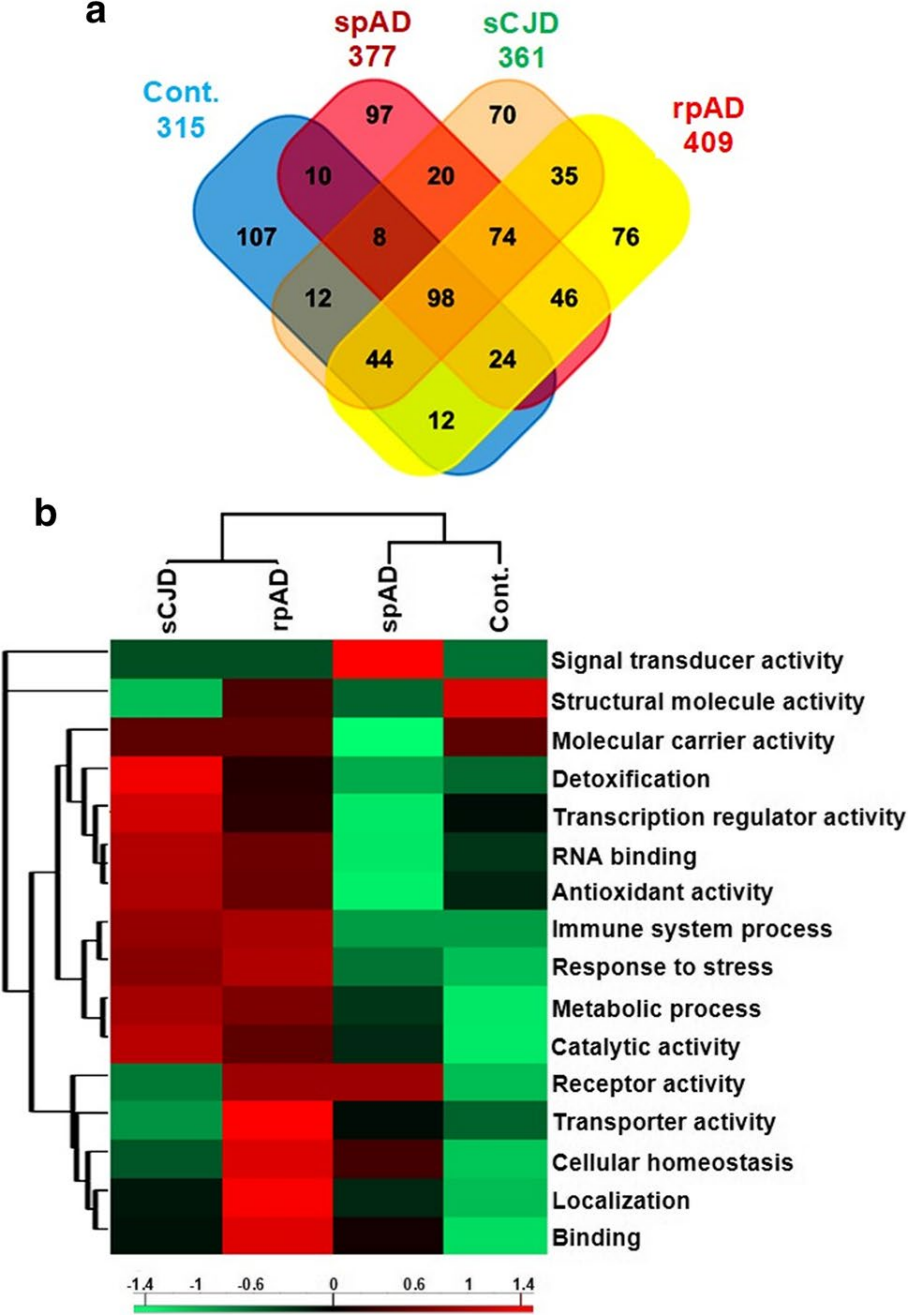
Comparative RNA-binding proteome profiling and functional categorization of MS results. RNA-binding proteome was isolated and identified from the human brain frontal cortical region of 20 cases (spAD, rpAD, sCJD-MM1, sCJD-VV2 as well as controls). **a** Venn diagram representing unique and shared RBP candidates in all groups. **b** Functional classification of MS results: RBP candidates identified in each group were analysed for associated Gene ontology functional-terms in two domains “biological process and molecular function’’. The protein counts associated with GO-terms from each group were uploaded to Perseus software, to prepare heatmap showing relative enrichment of different functional categories across all the groups. The variation in each term across the groups was calculated by *Z*-score. The columns of heatmap are representing disease groups (vertical-axis) and rows are displaying functional terms (*x*-axis), with red indicating an enriched category as compared with other groups, and green indicating depleted terms

The proteins which were specifically enriched in each group were used to find out a comparative proteome profile of the RBP-candidates. This analysis revealed disease-subtype specific differences in the enriched RBP-candidates (Fig. 3a). There were 98 proteins that were shared among all the groups. The common and unique proteins from all the groups are shown in Fig. 3a (suppl. Table 6, online resource).

#### Functional categorization of RBP candidates

To gain functional insights into the identified RBP candidates, classification based on gene ontology (GO) annotations (biological process and molecular function) was performed. Based on clustering of GO-terms, rpAD and sCJD groups were segregated into a single cluster, showing similarities in the enriched functional-terms (Fig. 3b). The most enriched functional categories for both groups included RNA binding, antioxidant activity, immune system process and response to stress (e.g. response to oxidative stress, cellular response to stress) among others (Fig. 3b). The signal transducer activity was particularly enriched in the spAD group when compared with other groups. Additionally, one cluster of cellular processes (including transporter activity, cellular homeostasis, localization and binding) was particularly enriched in the rpAD group, showing more aggressive disturbances in the overall cellular homeostasis in the rpAD group.

#### Classification of known and putative-RBP candidates

**Fig. 4:**
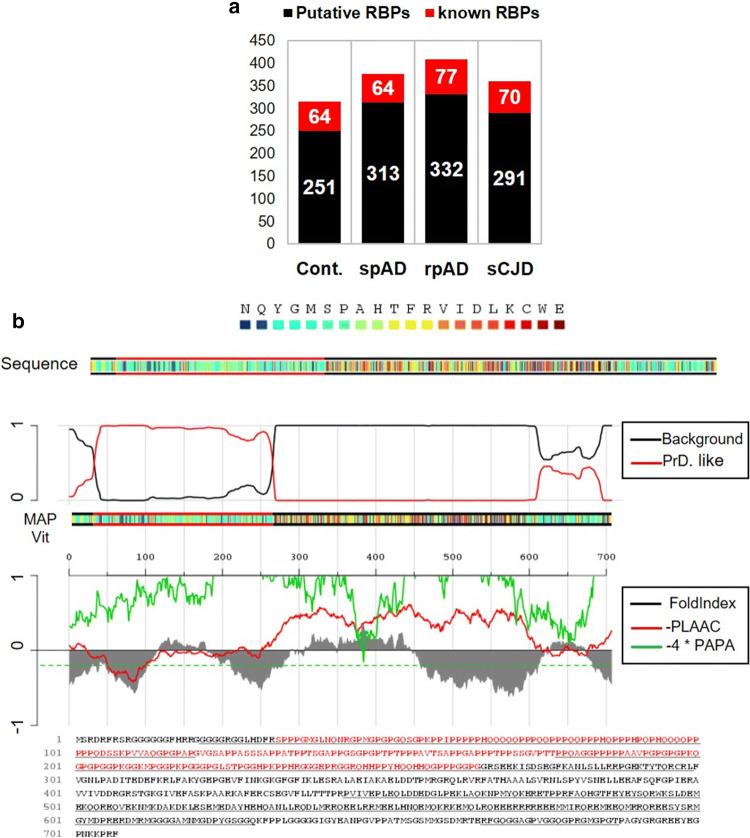
Identification of canonical and putative RBP candidates. RNA-binding protein candidates were isolated and identified from the human brain frontal cortical region of 20 cases (spAD, rpAD, sCJD-MM1, sCJD-VV2 as well as controls). **a** The identified proteomic candidates were searched for RNA-binding annotation in the UniProtKB database. The bar graph is representing two categories. The category I (red) indicates canonical RBPs (known), and category II (black bar) represents potential novel/putative RBP candidates from each group. **b** Identification of SFPQ-prion-like domain by PLAAC database. The amino acid sequence of SFPQ is represented in colour coded boxes. The red line in the top panel represents the probability of a prion domain against the background. The plots in the middle panel show fold-index scores in grey [60], the log-likelihood (LLR) ratio scores in red [2], and the predicted prion propensity (PPP) in green [81]. Negative scores represent disorder and prion propensity, dashed green line is indicating the cutoff value of PPP > 0.05. The bottom panel is showing the primary sequence of SFPQ with PLD in red colour [2]

In order to discriminate bona-fide RBPs from putative RBP candidates, identified proteins were classified, as demonstrated previously [11]. Two classes were organized to give clarity to the data (Fig. 4a). The class I (bona-fide RBP candidates: red bars) contains proteins that were having molecular function annotation as RNA-binding. There were 64 RBPs in the control group, 64 in spAD, 77 in rpAD and 70 in sCJD that were annotated as RNA-binding proteins. The class II (putative-RBP candidates: black bars) (Fig. 4a) contains the rest of the proteins, which include metabolic and catalytic enzymes among others. It should be noted that some of the identified candidate proteins may not have RNA-binding activity themselves, but, instead, interact with other proteins that do, and are hence identified in the RBPome.

#### Prion-like domain (PLD) prediction

Prion-like domains (PLD) are often present in the RBPs that carry out protein aggregation in neurodegenerative diseases, e.g. amyotrophic lateral sclerosis (ALS) and, more recently, in Alzheimer’s disease [40, 86, 87]. To evaluate the presence and biological significance of PLD-containing proteins in our dataset, identified RBP candidates were analysed using highly-stringent computational algorithms [81, 92]. We used an updated web-based PLAAC (Prion Like Amino Acid Composition) database to identify proteins with prion-like amino acid composition in our data-set. Criteria for a protein to have a PLD consisted of four requirements: it had to (1) be rich in Q/N-sequences, (2) exhibit disorderliness (according to PAPA algorithm) [60], (3) have compositional similarity with yeast prion domains, and (4) contain short amyloidogenic stretches able to carry out their self-assembly into an amyloidogenic state. Applying these criteria, we identified 24 PLD-containing proteins in the current study (suppl. Table 3, online resource). PLD of one of these proteins **(**SFPQ) is shown in Fig. 4b. Given that, SFPQ exhibited quite high PLD score, we further identified the amyloidogenic stretch in the sequence of SFPQ with algorithm ArchCandy (suppl. Figure 4, online resource).

### Pathological characterization of target RBP– SFPQ– in the postmortem brains

From the PLD-containing proteins described in the suppl. Table 3 (online resource), we selected SFPQ based on specific enrichment of this protein in rapidly progressive forms of dementia (rpAD and sCJD). Further, SFPQ exhibited a high score for PLD, which is a very crucial factor contributing to the pathophysiology of RBPs. TIA-1 (T-cell intracellular antigen 1) was also added as a target candidate for further investigation, given that its role in tau-aberrations in AD [4], although it was not detected in our MS dataset (most likely due to hydrophobic nature of the protein).

In order to explore specific mechanisms by which our selected target candidates (particularly SFPQ) are involved in the disease pathogenesis, and to further ascertain whether our selected approaches were appropriate for target selection, we began to analyze these proteins in more detail.

#### SFPQ and TIA-1 are dysregulated in rpAD and sCJD brains

**Fig. 5:**
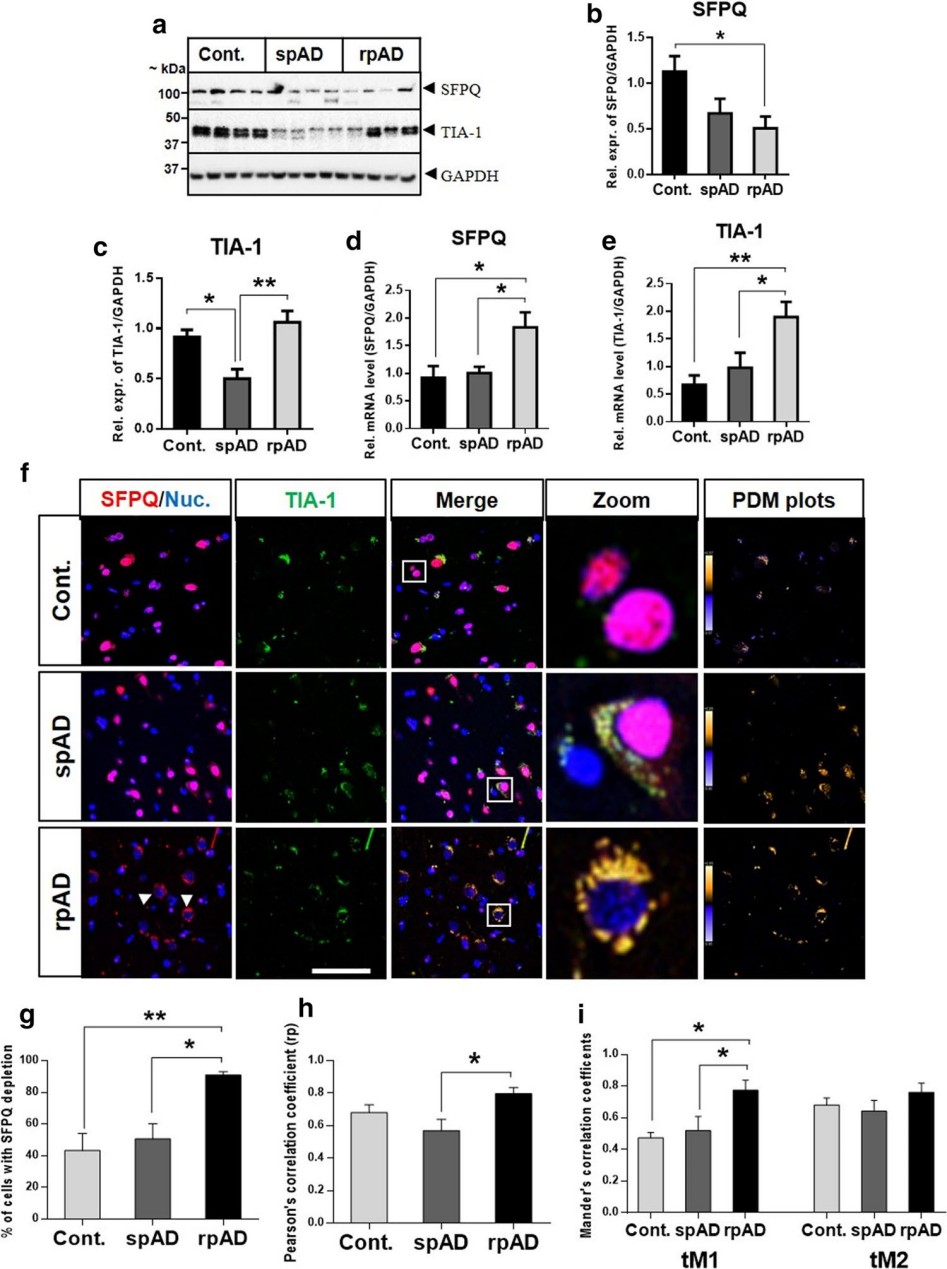
Differential expression analysis of SFPQ and TIA-1 at protein and mRNA level. **a** Representative immunoblot images. Immunoblotting analysis was performed with frontal cortical human brain tissues from spAD (*n* = 8), rpAD (*n* = 6), sCJD (-MM1 & -VV2 subtypes, *n* = 8) and non-demented controls (*n* = 8). **b**, **c** The densitometric analysis of SFPQ and TIA-1. **d**,** e** Expression of SFPQ and TIA-1 at mRNA level was analysed in spAD, rpAD, and controls with qRT-PCR (*n* = 5–8). GAPDH was used to normalize the expression levels of mRNA. The comparative Ct method (2^^−ΔΔCt^) was used for calculation of relative mRNA levels [45]. **f** Dislocation of SFPQ from the nucleus and colocalization with SG-marker-TIA-1. Co-immunofluorescence of SFPQ (red) and TIA-1 (green) in the human brain of spAD (*n* = 4), rpAD (*n* = 4), and controls (*n* = 5). Cell nuclei were visualized with To-Pro-3 iodide staining (blue). Representative images are shown (scale bar = 50 um). Intensity correlation analysis (PDM plots) was performed with ImageJ (WCIF Plugin). **g** Quantification of the cells showing SFPQ dislocation/depletion (*n* = 150/group). **h** Pearson’s correlation coefficient (rP) graph, showing significant colocalization between SFPQ and TIA-1 in rpAD cases in comparison to spAD cases.** i** Colocalization analysis with Threshold Mander’s correlation coefficients (tM1 & tM2). The value of tM1 represents the overlap of TIA-1 channel pixels with SFPQ channel pixels, and tM2 represents the overlap of SFPQ channel pixels with TIA-1 channel pixels. The colocalization analysis was performed with FIJI software (Coloc 2). One-way ANOVA and Tukey post-hoc test for multiple comparisons was used to calculate significance, *p < 0.05, **p < 0.01

As neurons are very sensitive to aberrant dosage and dynamics of RBPs [22], firstly we investigated the differential expression of SFPQ and TIA-1 in the human brain. Immunoblotting analysis was performed in the frontal cortical brain tissues from spAD, rpAD, sCJD-MM1, and sCJD-VV2 subtypes, as well as non-demented controls. The expression of SFPQ was reduced in the frontal cortex of rpAD (Fig. 5a, b) and sCJD subtypes (suppl. Figure 2a, b, online resource), compared with controls. A significant reduction was observed for TIA-1 levels in the spAD cases (Fig. 5a, c) and both subtypes of sCJD (-MM1 & -VV2) (suppl. Figure 2a, c, online resource), in comparison to control cases. In contrast, the levels of TIA-1 were significantly elevated in rpAD group in comparison to spAD group (Fig. 5a, c).

Dysregulation of SFPQ and TIA-1 at protein level prompted us to investigate their expression at the mRNA level. Quantitative RT-PCR analysis was performed to examine mRNA levels. Contrary to a reduced expression at the protein level in rpAD brains, SFPQ was significantly elevated at mRNA level, in comparison to control and spAD groups (Fig. 5d). For TIA-1 mRNA levels, a significant increase was observed in rpAD cases, in line with an increase at protein level (Fig. 5e).

#### SFPQ dislocation and colocalization with SG marker TIA-1 in the rpAD brain

Mislocalization of RBPs has been identified as a pathological feature in several neurodegenerative disorders [10, 12, 56, 84]. Next, we sought to explore the localization of SFPQ in postmortem brains. In order to study the localization of SFPQ, we immuno-stained the frontal cortical brain tissues from spAD, rpAD, and control cases. In most cells of the control and spAD brains, SFPQ was localized in the nucleus, the typical localization of SFPQ, as reported previously [53]. The typical cells were zoomed-in for closer details (see Fig. 5f, right panel). Strikingly, in rpAD cases, a massive dislocation of SFPQ was identified from the nucleus. A ring-shaped SFPQ was observed around the nuclei (see arrows in Fig. 5f, zoom-in section). A higher dislocation rate was observed in rpAD cases (91% cells), in comparison to spAD (51%) and control cases (43%) (Fig. 5g). These results indicate that nuclear depletion and dislocation of SFPQ occurred in both control and patient brains; however, the dislocation rate was particularly higher in rpAD brains.

Recently, mislocalization of some nuclear factors has been related to their cytoplasmic accumulation in the SGs [10, 84]. To test this possibility, we used co-immunofluorescence with primary antibodies specific for SFPQ (red) and TIA-1 (green): a classical marker of SGs (Fig. 5f). Sudan black was used to quench lipofuscin fluorescence, as differentiation of SG reactivity from lipofuscin can be quite challenging [46, 86]. TIA-1 immunoreactivity was also observed in control cases and a partial colocalization was also observed with SFPQ (Fig. 5f, zoom-in section). In spAD cases, a moderate colocalization was observed. For rpAD cases, a significant colocalization was identified between SFPQ and TIA-1 in the perinuclear/cytoplasmic area (Fig. 5f, zoom-in panel).

Multiple methods were used to provide a quantitative measure of the extent of colocalization between SFPQ and TIA-1, using FIJI (Coloc2) and image J (WCIF plugin) software. We examined the colocalization between SFPQ and TIA-1 using Pearson’s correlation coefficient (rP), Threshold Mander’s coefficients (tM1, tM2) [14], and intensity correlation analysis (ICA). ICA (PDM plots) showed highest degree of colocalization between SFPQ and TIA-1 in the rpAD brains, followed by spAD and controls (Fig. 5f, right panels). A significant degree of colocalization was observed in rpAD cases, compared with spAD, by both colocalization coefficients (rP and tM) (Fig. 5h, i). The value of tM1 shows the overlap of TIA-1 channel pixels with SFPQ channel pixels, which is significantly higher in rpAD when compared with either spAD or control cases. For tM2 (representing the overlap of SFPQ channel pixels with TIA-1 channel pixels), an increasing trend was also observed for rpAD cases. In summary, quantitative analysis of the co-immunofluorescence demonstrated that, colocalization between SFPQ and TIA-1 can be observed in both control and patients, with a specifically stronger degree of colocalization in rpAD cases.

#### SFPQ is colocalized with p-tau and oligomeric-tau in the rpAD brain

**Fig. 6:**
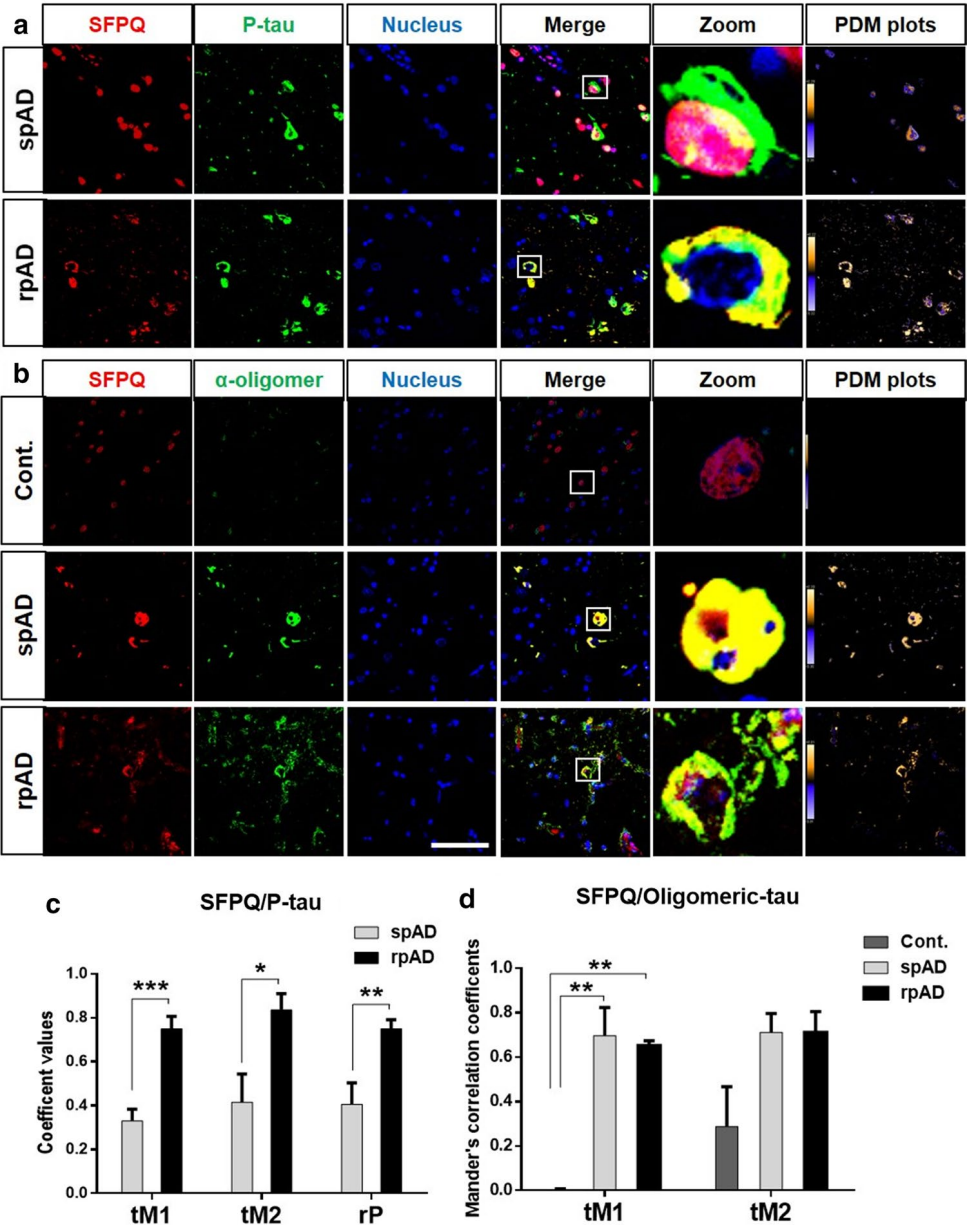
SFPQ colocalize with p-tau tangles and oligomeric-tau in rpAD brains. **a** Representative images stained with SFPQ (red) and α- p-tau (S199) (green) antibodies (scale bar = 50 μm) in spAD (*n* = 3) and rpAD (*n* = 3). Sections were counter stained with To-Pro-3 iodide to visualize nuclei (blue). PDM plots were prepared by intensity correlation analysis (ICA) using Image-J (WCIF plug-in). **b** Co-immunofluorescence images from control (*n* = 3), spAD (*n* = 5) and rpAD (*n* = 3) cortical sections, stained with α-SFPQ (red) and α-Tau oligomeric antibody: T-22 (green). PDM plots showing colocalization. **c** Pearson’s correlation coefficient (rP) and threshold Mander’s correlation coefficients (tM1& tM2) representing significant colocalization between SFPQ and p-tau in the rpAD group, in comparison to spAD group. Statistical significance was calculated by *t*-test, *p < 0.05, **p < 0.01, ***p < 0.001. **d** Threshold Mander’s correlation coefficient’s (tM1, tM2) showing significant colocalization between SFPQ and oligomeric-tau in both spAD and rpAD, as compared with control. Graphs were prepared with GraphPad Prism (6.01) using One-way ANOVA and Tukey post-hoc test for multiple comparisons, **p < 0.01

Cytoplasmic co-aggregation of some splicing factors with tau protein has been reported in both sporadic and familial cases of AD [7, 25, 85]. To test this possibility, we studied the relationship of SFPQ with tau-tangles using immunohistochemical analysis. A change in the fluorescence pattern was observed for SFPQ and p-tau, and typical cells were zoomed-in for closer details. In the human brain of spAD patients, SFPQ immunopositivity was detected in the nucleus and for p-tau tangles in the cytoplasm, with an occasional overlap between both in the nuclear region (Fig. 6a). In rpAD cases, two interesting findings were observed. Firstly, colocalization between SFPQ and p-tau was significantly increased compared with spAD group (Fig. 6c). Secondly, this colocalization was extranuclear rather than colocalization in the nuclear region that was observed in spAD cases.

Although, accumulation of cytoplasmic tau-tangles is a burden for the cell, it has recently been demonstrated that toxic-soluble oligomeric species of tau are the real culprits associated with cognitive decline, neuronal dysfunction, and death [31, 70]. In order to investigate the association of SFPQ with oligomeric-tau, we co-immuno-stained the cortical sections from control, spAD and rpAD cases (given that a significant association of SFPQ with tau-tangles was observed in the rpAD brain) with SFPQ and anti-oligomeric antibody for tau (T-22). Almost no reactivity was observed for oligomeric-tau in the control cases (Fig. 6b). Interestingly, the co-immunofluorescence analysis revealed a significant colocalization between SFPQ and tau-oligomers in both spAD and rpAD cases (Fig. 6b). The degree of colocalization was estimated with ICA (PDM plots) and Threshold Mander’s correlation coefficients (tM1, tM2) (Fig. 6d). Significant colocalization was identified in both spAD and rpAD cases by both methods.

### Role of SFPQ in stress response

**Fig. 7:**
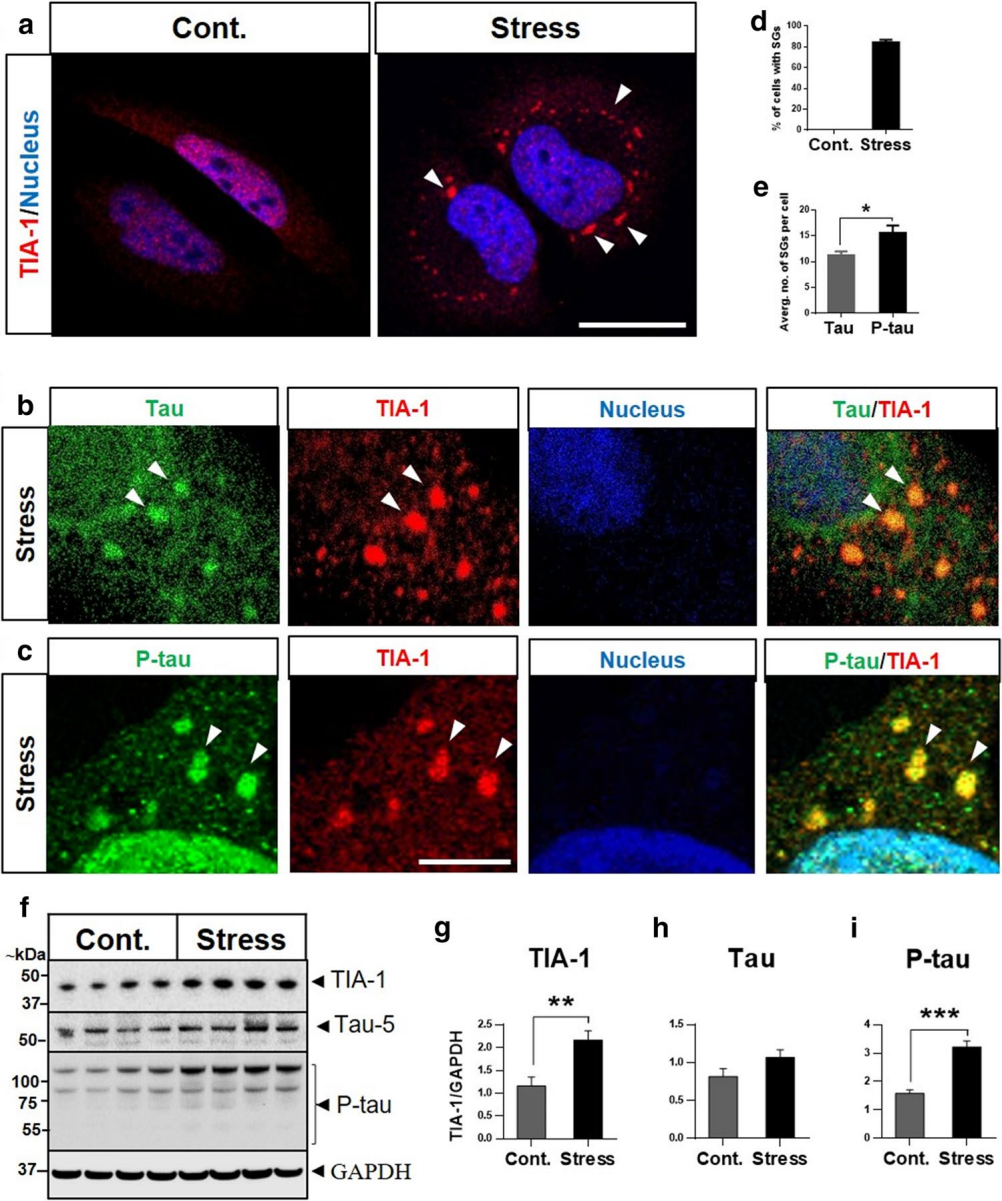
Sodium arsenite induces SGs in HeLa cells. **a** SGs were visualized by staining classical marker of SGs: TIA-1 in untreated (control) and sodium arsenite-treated (0.6 mM; 60 min) (stress) cells. The cells were counter-stained with DAPI to visualize nuclei, scale bar = 10 μm. **b**, **c** Tau and p-tau are recruited into SGs. The cells were co-immuno-stained with primary antibodies specific for total-tau, p-tau and TIA-1. **d** The cells positive for SGs were calculated with FIJI software. More than 80% cells were identified positive for SGs after stress treatment. **e** Tau and p-tau-positive SGs per cell (average no. of SGs) were calculated using FIJI software. Significance was calculated by t-test, *p < 0.05. **f**–**i** Stress induced increase in tau-phosphorylation and TIA-1 levels. Representative immunoblots for TIA-1, total-tau and p-tau in control (untreated) and stress (arsenite treated) cells. Statistical significance was calculated with *t*-tests **p < 0.01, ***p < 0.001

In order to investigate the significance of SFPQ mislocalization and association with the SG-marker TIA-1, a cellular model of stress was established in HeLa cells. A well-known oxidative stress-inducer, sodium arsenite, was used for stress induction. SGs were analysed, where the viability of stressed (arsenite treated) cells was not compromised (suppl. Figure 2h, online resource). After stress induction, cells were stained with a classical marker of SGs (TIA-1) to visualize SGs (Fig. 7a). Oxidative stress treatment induced the formation of clearly-defined cytoplasmic-foci positive for TIA-1 staining in more than 80% of cells (Fig. 7a, d).

Previously, it has been reported that SGs play an important role in tau aggregation [86]. Next, we examined the relationship between tau and SGs in our cellular model of stress by immunocytochemistry. Control (untreated) cells showed a positive signal for total-tau (tau-5) predominantly in the cytoplasm (suppl. Figure 3a, online resource). For p-tau (S199), a positive signal was detected in the cytosol and nucleus (suppl. Figure 3b,, online resource), with predominance in the nucleus, which is in-line with previous studies conducted in HeLa cells [36, 73]. Such a signal was increased upon oxidative stress induction. Stress treatment induced formation of both tau and p-tau granules, which showed colocalization with TIA-1 cytoplasmic SGs (Fig. 7b, c, zoom-in sections). The average number of SGs in each cell was estimated with FIJI-software. We identified a higher number of p-tau positive SGs, compared with tau-positive granules (Fig. 7e). Immunoblotting analysis revealed a significant increase in the intensity levels of TIA-1 in stress (arsenite treatment) cells, compared with controls (Fig. 7f, g). There were no significant changes observed for total-tau levels (Fig. 7f, h). For p-tau (S199), stress treatment induced significant increases in the levels of phosphorylation when compared with control (untreated) cells (Fig. 7f, i). This was found to be within HMW range (65–250 kDa), suggesting tau aggregation [76] under stressful conditions.

To rule out the possibility that the observed increase in the tau-phosphorylation in HeLa cells is cell line-specific, we also investigated tau-phosphorylation in the neuronal cell-line SH-SY5Y and HEK-293 cell line, after stress induction. Similar increases in the phosphorylated-tau (S199) levels in the HMW-range were identified in both cell-lines (suppl. Figure 2d–g, online resource). Overall, these results indicate that oxidative stress treatment increases tau-phosphorylation in all the cell lines tested.

#### Endogenous SFPQ redistributes into cytoplasm and assembles with SGs upon oxidative stress treatment

**Fig. 8:**
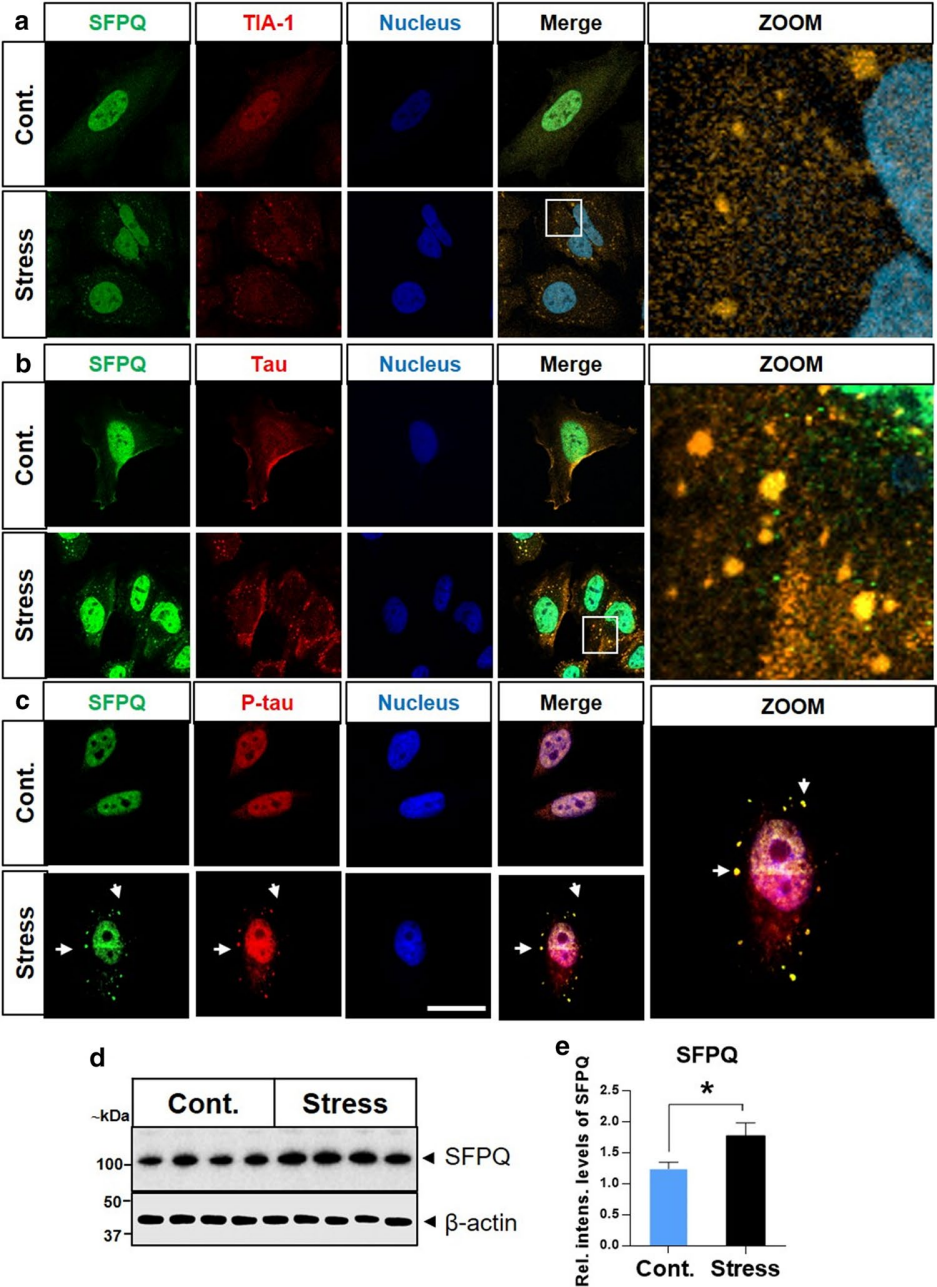
Recruitment of SFPQ into SGs after oxidative stress induction in HeLa cells. **a** Localization of SFPQ (green) and TIA-1 (red) was investigated using co-immunofluorescence, in sodium arsenite-treated (0.6 mM; 60 min) (stress) and untreated (control) cells. Cells were counter-stained to visualize nuclei (blue), scale bar = 10 μm. **b**, **c** SFPQ colocalizes with tau/p-tau in SGs after sodium arsenite induced oxidative stress in HeLa cells. Zoomed-in images of stress (arsenite treated) cells showing the overlap between SFPQ/tau in the cytoplasmic granules. **d** Representative immunoblot image for SFPQ after stress treatment. Intensity levels were normalized with β-actin. **e** The densitometric analysis was performed with Image Lab software. Statistical tests (*t*-tests) were applied in the GraphPad prism (6.01), *p < 0.05

To discover whether or not endogenous SFPQ forms SGs (as colocalization between SFPQ and TIA-1 was observed in the human brain), we examined subcellular localization of SFPQ after stress induction. SFPQ was primarily localized in the nucleus, in the control (untreated) cells, typical localization of SFPQ (Fig. 8a). Though the nuclear localization of SFPQ was not altered after arsenite treatment, stress (arsenite exposure) induced redistribution/translocation of SFPQ into granules in HeLa cells (arrowheads, Fig. 8a). To identify whether these SFPQ granules were actually SGs, HeLa cells were co-immuno-stained with the SG marker TIA-1. Colocalization was identified between the TIA-1 and SFPQ (yellow foci in the cytoplasm) (Fig. 8a, zoom-in section). The amount of cytoplasmic SFPQ signal was low because labelling only detected endogenous SFPQ, and SFPQ that was present in the cytoplasm was largely in the inclusions.

Overall, these results reveal that stress induces cytoplasmic redistribution and formation of SFPQ inclusions, which colocalize with TIA-1 positive SGs. To further confirm the granule formation propensity of SFPQ, we used catGRANULES algorithm (which assess the granule formation probability of a given protein on the basis of liquid–liquid phase (LLPS) separation property). A score of 1.66 was identified even higher than TIA-1(0.973), showing a high probability of SFPQ to form granules (suppl. Figure 3c, d, online resource).

#### SFPQ and tau/p-tau colocalize in the cytoplasmic granules

Recruitment of SFPQ into SGs and localization of tau in these SGs, raised the possibility that SFPQ and tau might colocalize in SGs and this functional interaction between SFPQ and tau might have implications for neurodegeneration. In the control cells, SFPQ was detected predominantly in the nucleus, while a translocation was identified in the granules in treated (stress-induced) cells. Colocalization between SFPQ and tau-5 was observed under both basal conditions (control) and in the granules after stress treatment (Fig. 8b, zoom-in section). Likewise, colocalization between SFPQ and p-tau (S199) was observed under control conditions and in cytoplasmic granules after stress treatment (Fig. 8c). Immunoblotting analysis indicated a significant increase in the intensity levels of SFPQ after stress induction, in comparison to control (untreated) cells (Fig. 8d, e).

#### SFPQ downregulation induced by human tau-expression

Braak stage-dependent reduction has been reported in the levels of SFPQ in the entorhinal cortex (EC) of AD cases [39]. In order to explore a direct pathological role of tau on SFPQ, we expressed human tau, both wild type (WT-tau) and mutated-tau (P301L-tau), transiently in HeLa cells.

**Fig. 9:**
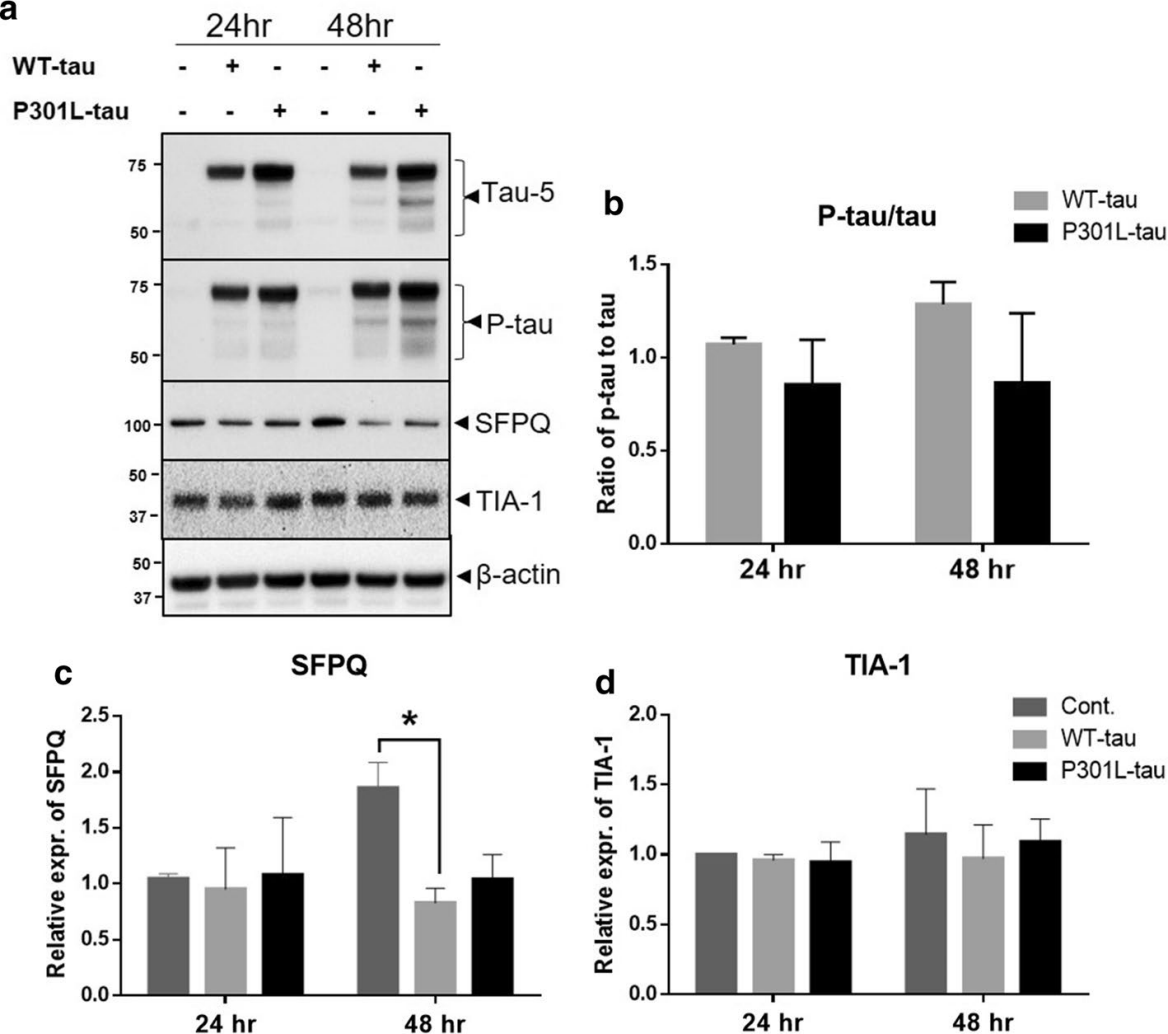
Human tau-induced downregulation of SFPQ in HeLa cells detected by immunoblotting. **a**–**d** Representative immunoblot images for tau, p-tau, SFPQ and TIA-1 after transient transfection of WT-tau or P301L-tau in HeLa cells. Densitometric analysis was performed with Lab image software. Statistical significance was estimated with one-way ANOVA and Tukey post-hoc test for multiple comparisons, *p < 0.05

To assess the expression of tau after transfections, immunoblotting analysis was performed at 24- and 48 h post-transfection. Protein extracts from transfected cells showed an increased level of total-tau and its phosphorylated form (S199) (Fig. 9a). There were no significant differences observed in the ratio of p-tau to tau between WT-tau and P301L-tau transfected cells (Fig. 9b). A trend of increased phosphorylation could be noted for WT-tau transfected extracts, in comparison to controls (Fig. 9b). Interestingly, expression of human-tau led to a significant reduction in the levels of SFPQ at 48 h post-transfection in WT-tau transfected cells, compared with controls (Fig. 9a, c). For P301L-tau expressing cells, a decreasing trend was also observed at 48 h post-transfection. There were no significant changes observed in the levels of TIA-1 protein under any of these conditions (Fig. 9a, d). MTS assay revealed a significant reduction in cell viability after tau-expression for both WT-tau and P301L-tau expressing cells, with more robust changes for WT-tau expressing cells at 48 h post-transfection (suppl. Figure 2i, online resource). Overall, immunoblotting analysis indicated that tau has a direct effect on SFPQ in the form of reduction, which coincides well with the massive reduction of SFPQ observed in the rpAD and sCJD patient’s brains.

##### Proteomic changes associated with SFPQ downregulation after human tau-expression

**Fig. 10:**
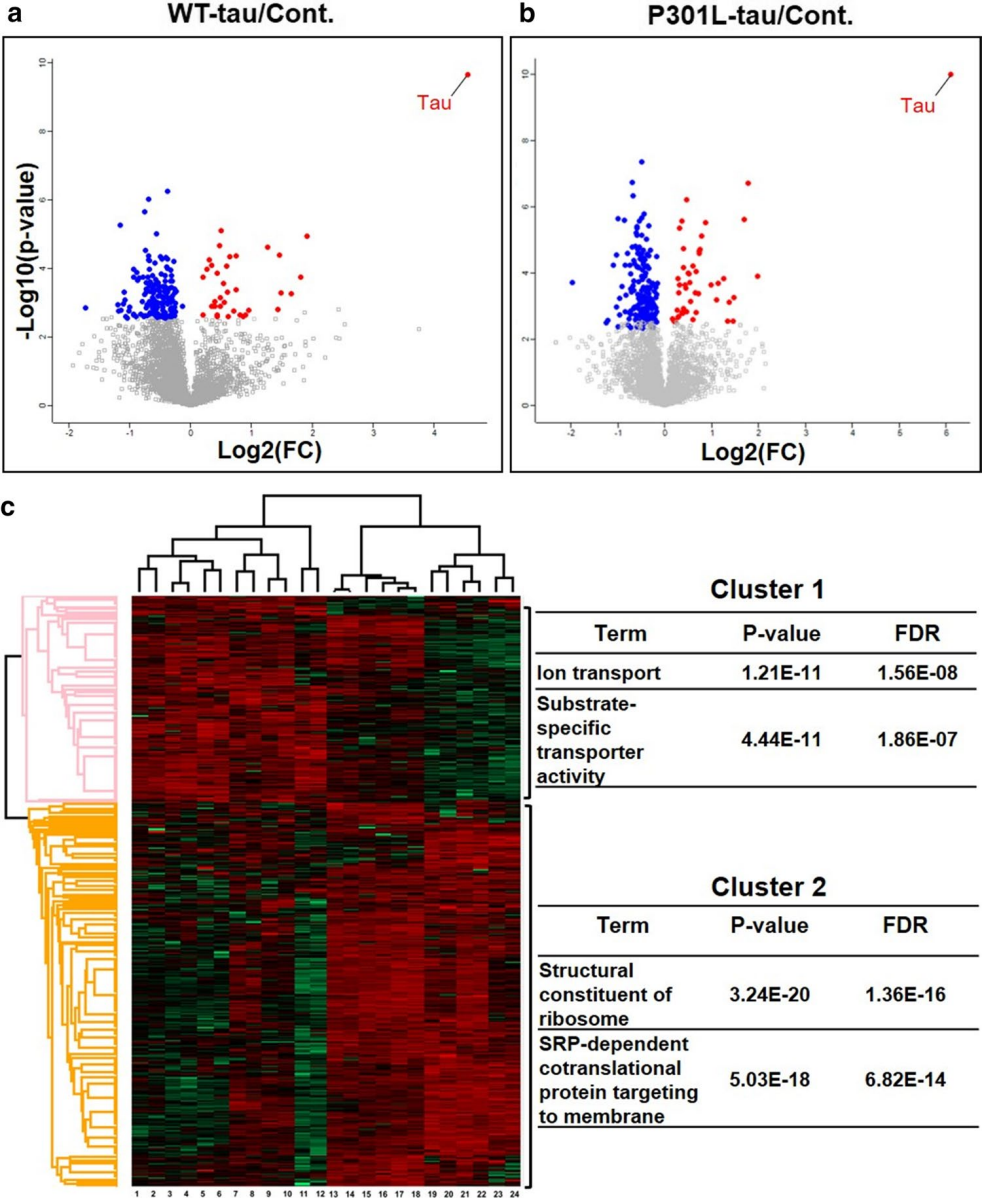
Human tau-expression induced proteomic alterations: **a**, **b** Volcano plots of pairwise comparisons (*t*-tests with BH correction), showing the -log10-*p*-values (*y*-axis) and the log2(FC) of the proteins that were differentially expressed (*x*-axis) in both WT-tau and P301L-tau expressing cells in comparison to controls. The data points depicted in red are showing upregulated proteins and blue is representing downregulated proteins. **c** The heatmap of hierarchical clustering analysis of 314 DEPs among technical and biological replicates generated by Perseus software. The Log2-transformed expression values of significantly DEPs were normalized by *Z*-score. The columns are representing samples (tau-expressing cells: lanes 1–12 & control: lanes 13–24) and rows indicating significantly modulated proteins (cluster1, up-regulated (red), cluster2, down-regulated (green). The enriched functional terms (Fisher’s exact test with FDR multiple test correction) with their *p*-values and FDR values are shown on the right side

Human-tau expression induced SFPQ downregulation in vitro*,* in the present study. To investigate the combinatorial effect of tau-toxicity and SFPQ-reduction hallmarks at 48 h post transfection, we employed a quantitative proteomics approach (SWATH-MS), which has emerged as a powerful technology for investigation of cellular signalling pathways [35, 44]. SWATH-MS identified 3597 proteins quantitatively at a critical FDR of 1%. Bioinformatics analysis was performed using Perseus software to identify differentially expressed proteins (DEPs). Proteins showing a corrected Benjamini Hochberg corrected *p*-value < 0.05 were considered differentially expressed (Fig. 10a, b). DEPs (314) were divided into two sets: the first consisted of 63 up-regulated proteins, and the second consisted of 251 down-regulated proteins (suppl. Tables 7 and 8, online resource).

A number of bioinformatics tools were used to examine enriched functional categories and pathways after human tau expression. We performed hierarchical clustering analysis and Fisher’s exact test by Perseus software. As shown in Fig. 10c, DEPs were clustered hierarchically across all the samples (columns). The main branch point in the columns separated the tau-expressing cells (Fig. 10c, Lanes 1–12) from controls (mock-transfected or non-transfected controls) (Fig. 10c, Lanes 13–24). The DEPs (rows) were grouped into two main clusters. Cluster-1 included proteins that were upregulated in tau-expressing cells (both WT-tau and P301L-tau). Most significant functional terms enriched for cluster-1 were ion transport and substrate specific transporter activity (Fig. 10c: right pane). The downregulated proteins (cluster-2) exhibited enrichment for functional-terms related to -structural constituent of ribosome and -SRP dependent co-translational protein targeting to membrane among other terms (Fig. 10c: right panel).

*Ingenuity pathway analysis (IPA)* Then, ingenuity pathway analysis (IPA, Qiagen, USA) was performed to identify major canonical pathways modified after human tau-expression. According to *p*-values, the top five significant pathways identified in WT-tau expressing cells as compared with mock transfected cells were; eukaryotic initiation factor 2 (eIF2) signalling (*p*-value = 2.28E−23), regulation of eIF4 and p70S6K signalling (*p*-value = 6.44E−07), mTOR signalling (*p*-value = 5.60E−06), mismatch repair for eukaryotes (*p*-value = 4.93E−05) and proline biosynthesis I (*p*-value = 1.23E−04).

The most significant canonical pathways modulated after P301L-tau expression were EIF2 signalling (*p*-value = 3.23E−18), interferon signalling (*p*-value = 2.86E−04), mTOR signalling (*p*-value = 9.50E−04), DNA double-strand break repair by non-homologous end joining (*p*-value = 1.11E−03), and telomere extension by telomerase (*p*-value = 1.28E−03). Overall, EIF2 signalling was the most-significant pathway in both WT-tau and P301L-tau expressing cells.

*Disease- and function-based protein networks* In addition to pathway mapping, DEPs were also categorized into related disease- and function-based protein networks. The most significant networks associated with significantly-modulated proteins are listed in the supplementary material (suppl. Tables 9 and 10, online resource) for both WT-tau and P301L-tau expressing cells, respectively. The most significant network based on highest percentage of focus molecules (score = 61), that is also shared between WT- and P301L-tau expressing cells is—RNA damage and repair, protein synthesis, cancer—consisting of 27 focus molecules, including 60S ribosomal subunit, C7orf50, CDK4/6, Eif4g, ERK1/2, IFIT1, Importin beta40s subunit, RPL15, RPL18, RPL18A, RPL19, RPL23A, RPL27A, RPL31, RPL32, RPL36, and RPL37A among others. The second most-enriched network was—cell morphology, cellular assembly and organization, DNA replication, recombination, and repair network—consisting of 20 focus molecules from DEPs from proteomic dataset.

Proteomics investigation with functional characterization has provided a comprehensive overview of major alterations associated with tau toxicity and downregulation of SFPQ. Analysis of global proteomic alterations revealed two major themes—-RNA metabolism and protein synthesis, and -DNA homeostasis-related processes—that were significantly altered after human tau-expression.

### Dysregulation of SFPQ and TIA-1 in 3xTg-mice

Given the long incubation periods of clinically silent neurodegeneration, identification of the modifiable risk factors at early stages of the disease is crucial. To extend our results from the analysis of terminal stage pathology from the human brain, a time-dependent expression profile of proteomic signatures (tau, p-tau, SFPQ and TIA-1) was examined in a mouse model (3xTg mice model of combined Aβ and tau pathology), at 4 time-points corresponding to -3, -6, -9 and 12 months post-inoculation (mpi).

**Fig. 11:**
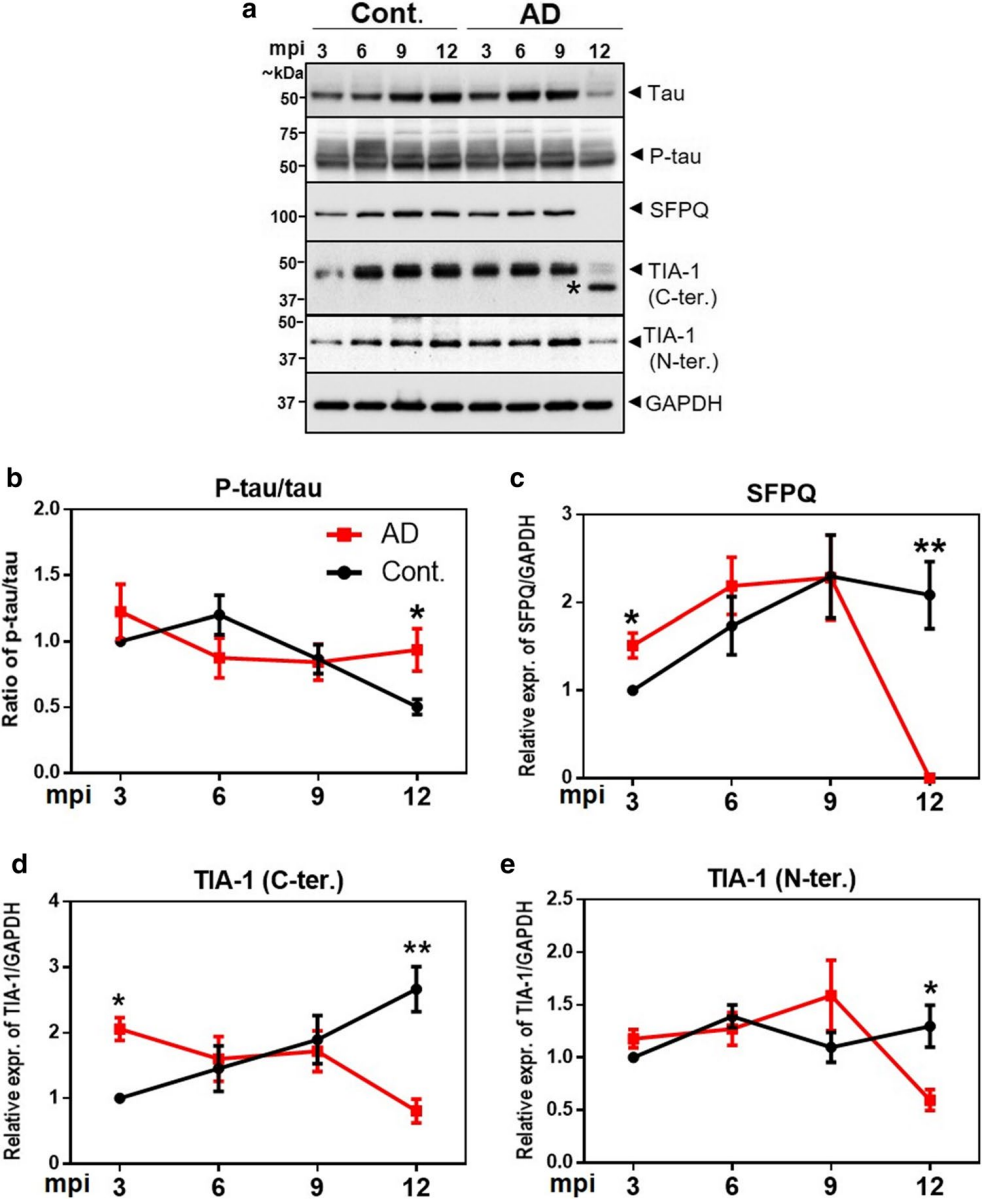
Temporal expression profile of total tau (tau-5), p-tau (S199), SFPQ and TIA-1 in 3xTg mice. **a** Representative immunoblot images showing expression of tau, p-tau, SFPQ and TIA-1 (C- & N-terminal specific antibodies) from AD (3xTg-inoculated, *n* = 4) mouse brain cortical tissues at the indicated ages (mpi: months post inoculation) and respective controls (3xTg-non-inoculated, *n* = 4). (*) We are unsure of this band. **b**–**e** The densitometric analyses were performed with Image Lab software. Unpaired t-tests were performed to calculate statistical significance at each time point. *p < 0.05, **p < 0.01

The ratio of p-tau to total-tau was significantly increased at the late stage of the disease between the experimental (3xTg-inoculated) and age-matched control (3xTg-non-inoculated) groups (*p* < 0.05) (Fig. 11a, b). Interestingly, SFPQ expression was significantly increased in comparison to controls at early stage after inoculation (3mpi) (Fig. 11a, c). At middle stages of the disease, no significant changes were observed and a direct reversal (a drastic reduction) was identified at late stage (12 mpi) of the disease (Fig. 11a, c). Within the sensitivity of the western blot, the band for SFPQ was not detectable (Fig. 11a), which coincided well with the reduced levels of SFPQ in the post-mortem brains, in rapidly-progressive forms of dementia (rpAD and sCJD).

Two different antibodies detecting C- and N-terminals of TIA-1 (TIA-1 abcam: recognizing amino acids 350 to the C-terminus; TIA-1 sc-166247 recognizing amino acids 37–65 at N-terminus) were used. The expression of TIA-1 was significantly decreased at terminal stages of the disease (12 mpi) for both antibodies (Fig. 11a, d, e). For TIA-1 (C-terminus) antibody, a significant increase was observed at the early stage of the disease (3mpi) (Fig. 11d). Overall, this time-point expression profile with prominent changes in SFPQ, tau and TIA-1 at 3mpi suggest a coordination of these molecules at the very early stages of the disease.

## Discussion

In this study, we identified and characterised RNA-binding proteome from human brain frontal cortex of three neurodegenerative disorders—spAD, rpAD, and sCJD (-MM1 & -VV2 subtypes) as well as control cases—using a brain-derived RNA-based pull-down approach followed by mass spectrometry analysis. One of these proteins—SFPQ is dysregulated and dislocated in the post-mortem brains of rpAD patients. SFPQ is predominantly a nuclear protein and a critical component of neuronal transport granules.

Proteomic study revealed characteristic qualitative and quantitative changes in the identified RNA-binding proteome in a disease subtype-specific manner. The differential enrichment analysis indicated distinct protein clusters specific to each group (Fig. 2g). Overall, this specific enrichment indicates differences in the RNA-RBP interactions in response to disease stress. These specific sets of proteins can be of potential relevance to identify subtype-specific differences in these heterogenous diseases. Of note, RBPome profile of rpAD, displayed major similarities with sCJD, compared with spAD (Fig. 2g). These results suggest that, both forms of rapidly progressive dementias (rpAD and sCJD) have resemblance in the RBP-mediated neuropathogenesis at the terminal stage of the disease.

The functional categories connected with—stress response and RNA binding—were enriched in rpAD and sCJD groups, compared with control and spAD groups, representing a more aggressive dysregulation of RBP-related processes. Stress, in various forms, has been associated with fast progression not only in AD but also in other neurodegenerative disorders, e.g. Huntington’s and Parkinson’s diseases [34]. Animal studies have also demonstrated stress as a contributing factor to the fast progression of AD, including increased accumulation of plaques, and tangle formation [29, 51]. Recently, stress response has also been linked to pathophysiology of tau protein in AD [19, 85], further strengthening the hypothesis that stress and post-transcriptional regulatory processes associated with RBPs are an integral feature of these neurodegenerative disorders.

In line with the previous reports [72], many putative RBP candidates were identified in the present study e.g. metabolic and catalytic enzymes among others. The identification of these putative RBPs in the RNA-binding proteome data set points towards a cross-talk between RBPs and other cellular proteins to meet the everchanging microenvironment within the neurons. Dysregulation of these intricate networks of proteins in the neurons may start a cascade of aberrant signaling, eventually leading to neurodegeneration. Many studies identifying RBPomes have reported similar putative RBP candidates, that have no prior linkage to RNA-associated functions [9, 11, 20]. These putative RBP candidates have been categorized as enigmRBPs. At this junction, the functions of most of these enigmRBPs as it relates to RNA are largely unknown. It is important to note that some of the identified putative RBP candidates (e.g. SNG3, HEBP1, SV2A) may not have RNA-binding activity themselves. They are identified in the current study due to binding with other proteins, that do have RNA-binding activity. Additionally, 24 prion-like domain (PLD) containing proteins, fulfilling the criteria to potentially behave as prion-like proteins were identified (e.g. SFPQ, BSN, EWS, PRIO, FUBP2) in the current study. PLDs are essential for RBP functions and enable them to undergo liquid–liquid phase separation, that is the basis of formation of higher-order structures, including oligomers and granules [13, 63]. PLDs carry out protein aggregation in the neurodegenerative disorders, e.g. amyotrophic lateral sclerosis (ALS) and, more recently, in AD [40, 85, 87].

In the present study, we observed that a PLD-containing protein (SFPQ) was exclusively identified in the RBPome of rpAD and sCJD groups, suggesting a potential role of SFPQ in rapidly progressive dementias. Indeed, we discovered dysregulation of SFPQ in association with tau and TIA-1 proteins, particularly in the rpAD and sCJD post-mortem brains.

Recent evidence has shown that neurodegenerative disorders can arise from alterations in dosage and dynamics of RBPs [22]. In the present study, down regulation of SFPQ at protein level in the frontal cortex of rapidly progressive dementias (rpAD and sCJD), suggest that SFPQ dysregulation may have a role in the rapid progression of these diseases. Ke et al. [39] demonstrated Braak stage dependent reduction in SFPQ levels in the entorhinal cortex (EC) of AD patients. Previously, SFPQ reduction has been linked with behavioral anomalies in mice, neuronal loss, and p-tau accumulation [37]. In another study, loss of SFPQ was found to lead to apoptosis in zebra fish, linking downregulation of SFPQ to neuronal cell death [48]. In contrast to the reduction at protein level, SFPQ expression was elevated at mRNA level in rpAD cases. One plausible explanation for this observation could be that transcription of SFPQ is increased in order to compensate for loss of SFPQ at the protein level. The elevated mRNA levels of SFPQ could also contribute directly to neurodegeneration: this excessive mRNA may sequester many proteins necessary for other cellular signalling, leading to translation defects among others, as has been noted for some other neurodegenerative disease associated proteins [30, 69, 79].

Further, the present study discovered a drastic nuclear depletion of SFPQ in the frontal cortex of rpAD cases. Specifically, higher dislocation rate (91% of cells) identified in rpAD cases in comparison to spAD (51%) and controls (43%), suggests an important role of SFPQ in the pathology of rpAD. A complete dislocation of SFPQ in the hippocampus of AD patients has been reported previously [39]. While, Lu et al. [49], demonstrated a moderate dislocation in AD brains, which was consistent with the observations regarding spAD in the present study. Based on data from the current study, from the frontal cortex and literature reports (showing dislocation in hippocampus), it can be concluded that dislocation of SFPQ is an important feature of Alzheimer’s pathology, and different brain regions have variable intensity of SFPQ dislocation. Increased dislocation in rpAD cases may be due to higher cell death (through apoptosis), as cytoplasmic localization of SFPQ has been observed in apoptotic cells [71]. SFPQ is predominantly a nuclear protein involved in multiple functions in the neurons, including transcription, alternative splicing, DNA-damage and repair, and RNA transport [41, 88]. Overall, dysregulation and nuclear depletion of SFPQ observed in rpAD cases, could contribute to neurodegeneration by both: loss of nuclear functions [49], and gain of toxic functions in the cytoplasm. To the best of our knowledge, this is the first study demonstrating dysregulation of SFPQ at both protein and mRNA level, and its dislocation from the nucleus in the frontal cortex of specifically rpAD cases. One potential limitation of these findings is that the regulation of proteomic targets could not be studied with reference to gender dependent differences due to limited availability of our cohort of control and patient cases. Given the explorative nature of the present study, further studies are needed with higher number of samples for a targeted approach.

SFPQ nuclear dislocation was associated with its cytoplasmic colocalization with SG-marker-TIA-1, p-tau tangles and oligomeric-tau in brain lesions of rpAD cases. Significant colocalization between SFPQ and p-tau in the rpAD brain in comparison with spAD, suggests involvement of different pathological mechanisms in subtypes of AD. Association of SFPQ with oligomeric-tau in both spAD and rpAD provides an intimate link of SFPQ to pathological tau. In line with the findings from the human brain, SFPQ and p-tau translocated from the nucleus and assembled into cytoplasmic TIA-1-positive SGs in response to oxidative stress stimuli in the cultures (HeLa cells). In the current study, HeLa cells were used as an appropriate model for stress induction. Several models have been used to understand SGs at cellular level, including recombinant cell lines (Aulas and Vande Velde [5]. A great deal of our knowledge of these cytoplasmic membrane-less foci (SGs) originates from studies in HeLa cells (154 publications, according to Aulas and Vande Velde [5]. Although, this is a non neuronal cell line, the cell line worked for the current study due to several advantages including; -large cytoplasmic volume of HeLa cells compared to other cell lines which allows accurate identification of cytoplasmic SGs, they are easy to maintain in culture and -reliably result in high transfection efficiency [8].

These findings from the current study indicate a role of SFPQ in the stress response under physiological conditions. The identification of SFPQ in the SG-interactome of U_2_OS cells [38], further confirms the involvement of SFPQ in the stress response. Redistribution and colocalization of SFPQ in cytoplasmic TIA-1-SGs provides a possible mechanism of SFPQ depletion/dislocation through pathological SGs. Pathological and persistent TIA-1-SGs have been implicated in AD [4]. This redistribution of SFPQ has been linked to cell cycle arrest [50], apoptosis [28, 71], and splicing abnormalities. All these evidences suggest that cells may use cytoplasmic activities of SFPQ as a gauge to cope with cellular crisis, while it might lead to more demise for the nuclear functions of SFPQ.

Cytoplasmic co-aggregation of tau protein with some splicing factors has been reported for both sporadic and familial AD cases [7, 12, 25]. In the current study, a significant cytoplasmic colocalization between SFPQ and p-tau (S199) in the rpAD brains, in comparison to spAD cases, suggest a change in the nuclear-functions of these proteins. We speculate that, at initial stages of disease, kinases can phosphorylate not only tau protein but also SFPQ. This phosphorylation can switch their localization, redistributing them either to the nuclear envelope or cytoplasm [49] and results in an overall “depletion’’ from the nucleus. As phosphorylation at C-terminal tyrosines leads to accumulation of SFPQ at the nuclear envelop or cytoplasm [50, 59]. Further, a complete depletion of nuclear-tau has been reported in the hippocampal neurons of AD patients [33]. Previously, pathological SGs have been linked to mislocalization of tau, FUS and TDP-43 [15, 24, 46, 85, 89].

Reduction in SFPQ levels after human-tau expression (0N4R), at 48 h post transfection in HeLa cells confirmed a causal role of tau in the downregulation of SFPQ. There were no significant changes observed in SFPQ levels at 24 h post transfection, in line with previous findings in SH-SY5Y cells [39]. Quantitative-MS (SWATH-MS) analysis from this model highlighted two major themes (DNA damage and repair and RNA-metabolism) that were altered as a result of combinatorial effect of tau-toxicity and SFPQ downregulation. To date, any role of tau protein in the maintenance of telomere has not been reported, the observed disturbances in the telomere maintenance, can be attributed to SFPQ reduction hallmarks. Overall, these findings confirm nuclear loss of function-abnormalities associated with SFPQ downregulation.

Finally, the present study uncovered that expression of SFPQ and TIA-1 is significantly elevated already at the early stage (3 mpi) in 3xTg mice model of combined Aβ and tau pathology, suggesting that these proteins could be of potential significance as early disease-modifying targets. This upregulation (of SFPQ and TIA-1) in mice is consistent with the acute-phase oxidative stress-induced increase in SFPQ and TIA-1 levels in HeLa cells, suggesting that at early stages of the disease stress associated with pre-tangle pathology might induce these changes [64]. A drastic reduction in SFPQ levels at late stage (12mpi) in 3xTg mice—in corroboration with reduced expression of SFPQ in post-mortem brains of both rapidly progressing diseases (rpAD and prion diseases); indicates that SFPQ could be of potential relevance to identify rapidly progressing forms of dementia. SFPQ reduction (knock down) leads to a decreased expression of amyloid precursor protein (APP) [77] in neuronal cells. Given the presence of extensive Aβ and tau pathology by 12 mpi and tau dependent reduction in SFPQ levels in our cellular study, indicates this reduction as a combinatorial effect of Aβ and tau pathology. Furthermore, a similar reduction in the levels of TIA-1, in both mice (at late stage of the disease) and spAD cases, suggest a compensatory/protective response adopted by the neurons. As reduction in TIA-1 (haplosufficiency) has been proven to be protective against neurodegeneration [4]. In contrast, a failure to adopt this protective response in rpAD cases, identified as a significant increase in TIA-1 levels in rpAD cases, in comparison to spAD cases, suggests higher neurodegeneration in rpAD cases.

## Conclusion

**Fig. 12:**
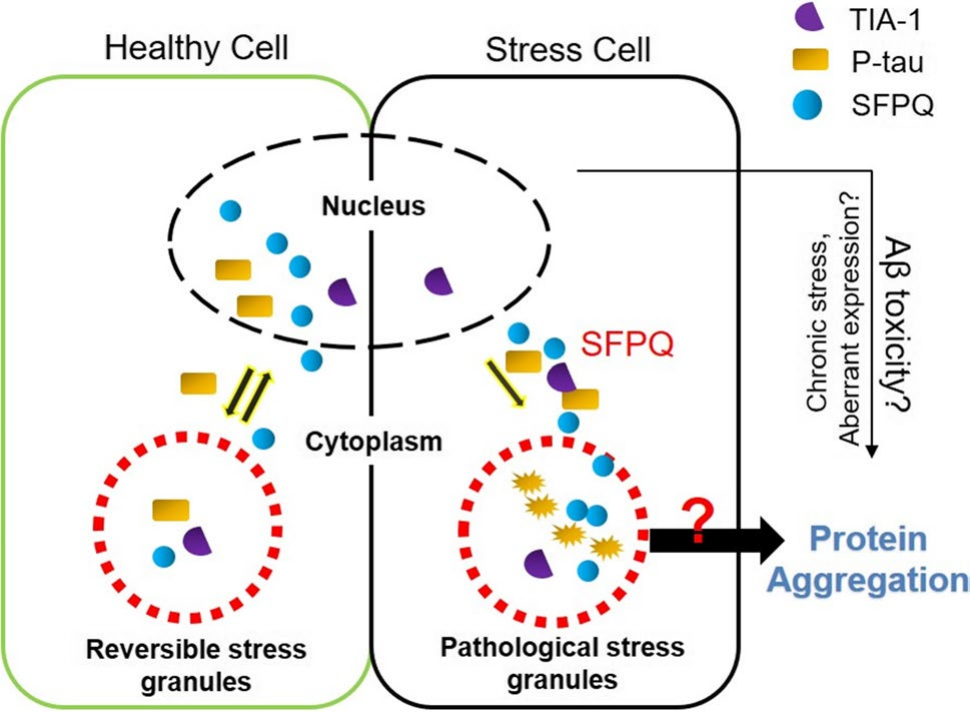
Current working model for SFPQ and tau-pathological features in rapidly progressive form of AD. The left box of the picture is depicting nuclear-cytoplasmic translocation of SFPQ and p-tau, and their localization in stress granules based on our data from the cellular model of stress. Oxidative stress induced redistribution of SFPQ and p-tau into cytoplasm, and recruitment into SGs. The right box is depicting pathological features of both proteins observed in the post-mortem brains of specifically rpAD cases. The nuclear dislocation of SFPQ was identified in the post-mortem brains of particularly rpAD cases. Further, SFPQ showed colocalization with tau-tangles, tau-oligomers and TIA-1 in the cytoplasm, in the human brain and also in cultures. Nuclear depletion of SFPQ can be toxic for the cells by both loss of function in the nucleus [49] and toxic gain of functions in the cytoplasm

Overall, our findings provide a comprehensive RBPome map (of spAD, rpAD and sCJD) and opens many new targets for exploration. The study has identified SFPQ, tau and TIA-1 as critical factors involved in rpAD pathology (Fig. 12). Co-immunofluorescence analysis demonstrated complete nuclear depletion of SFPQ, particularly in the rapidly progressive AD cases. This nuclear depletion was concomitantly associated with its cytoplasmic colocalization with p-tau (S199) tangles and oligomers of tau. In parallel with human brain findings, our in vitro study (cellular model of stress) clearly demonstrated the translocation of SFPQ and p-tau into cytoplasmic TIA-1-positive SGs, upon oxidative stress treatment. The expression of human tau (tau pathology model) in vitro induced significant downregulation of SFPQ, suggesting a causal role of tau in the downregulation of SFPQ. Finally, the 3xTg mice model study uncovered specifically very early changes (3mpi) of target proteomic signatures. The expression of SFPQ and TIA-1 was already significantly elevated at the early stages of the disease in the 3xTg mice model, suggesting that these proteins could be of potential significance as early therapeutic targets.

The re-establishment of the normal architecture of the nucleus, by treating SFPQ dislocation/depletion, could be an interesting candidate for future therapeutic research strategies. Results from the current study extend the pathological features of tau beyond microtubular and somatodendritic compartments to the nuclear processes.

## Electronic supplementary material

Below is the link to the electronic supplementary material.

Supplementary Fig.1 Patient cohorts used in the present study. a Comparison of age distribution of the diseased and control cases used in the current study. b Statistical analysis of RBPome data: Two different approaches (A and B) were adopted fora detailed analysis of RBPome identified by label free quantification-MS, from the human brain frontal cortical region of 20 cases (spAD, rpAD, sCJD-MM1, sCJD-VV2 as well as controls). Approach A: Differential enrichment analysis of RBPome candidates by Perseus software. Zero values from the total spectral counts of the proteins were imputed by half of the minimum value. Pairwise t-test comparisons were performed between all the group combinations to identify significantly-enriched proteins in each group. Approach B: Qualitative analysis of the identified RBPome. Proteins with a single quantitative value were included in this approach from all the groups to have a broader impression of the isolated proteome. A comparative RBPome profile was obtained to find out common and unique proteins in all groups.
Supplementary file1 (TIFF 558 kb)

Supplementary Fig. 2 Differential expression analysis of SFPQ and TIA-1. a Representative immunoblot images.Immunoblotting analysis was performed with frontal cortical human brain tissues from sCJD (-MM1 & -VV2 subtypes, n = 8) and non-demented controls (n = 8). b & c The densitometric analysis of SFPQ and TIA-1. One-way ANOVA was conducted,followed by Tukey post-hoc test for multiple comparisons. *p < 0.05, **p < 0.01. d-g Stress induced increase in tau phosphorylation in SH-SY5Y and HEK-293 cells. Representative immunoblots for p-tau in control (untreated) and stress (0.6mM NaASo2: 60 min) cells. Statistical significance was estimated by t-test **p < 0.01, ***p < 0.001. h Trypan-blue exclusion assay was used to estimate the cell viability after stress exposure. The percentage of viable cells was calculated in control(untreated) and stress cells. i The cell viability was measured by MTS assay, after expression of human-tau (both WT-tau and301L-tau) in comparison to controls at 24- and 48 hours post-transfection. One-way ANOVA followed by Tukey post-hoctest was used to calculate statistical significance, *p < 0.05, **p< 0.01, ***p < 0.001, ****p < 0.0001.
Supplementary file2 (TIFF 543 kb)

Supplementary Fig. 3 Stress induces tau and p-tau positive stress granules. a & b Tau and p-tau are recruited into TIA-1-positive SGs. The cells were co-immuno-stained with primary antibodies specific for total-tau, p-tau and TIA-1. Cells were counterstained to visualize nuclei (blue), scale bar = 25 μm for a and 10 μm for b. c & d Liquid-liquid phase separation properties of SFPQ and TIA-1 were assessed by catGRANULES algorithm.
Supplementary file3 (TIFF 1184 kb)

Supplementary Fig. 4 Prediction of the presence of amyloidogenic region in the SFPQ protein according to the algorithm ArchCandy. The height of the column on the graph corresponds to probability of formation of the amyloid structure by the corresponding protein region. The amino acids in the amyloidogenic sequence of the protein are highlighted in red.
Supplementary file4 (TIF 5095 kb)

Supplementary file5 (PDF 1158 kb)
