# Supplementary figures and images for: SFPQ and Tau: critical factors contributing to rapid progression of Alzheimer’s disease
Source: Acta Neuropathol. 2020 Jun 23;140(3):317–39. doi: 10.1007/s00401-020-02178-y (PMC7423812; doi:10.1007/s00401-020-02178-y)

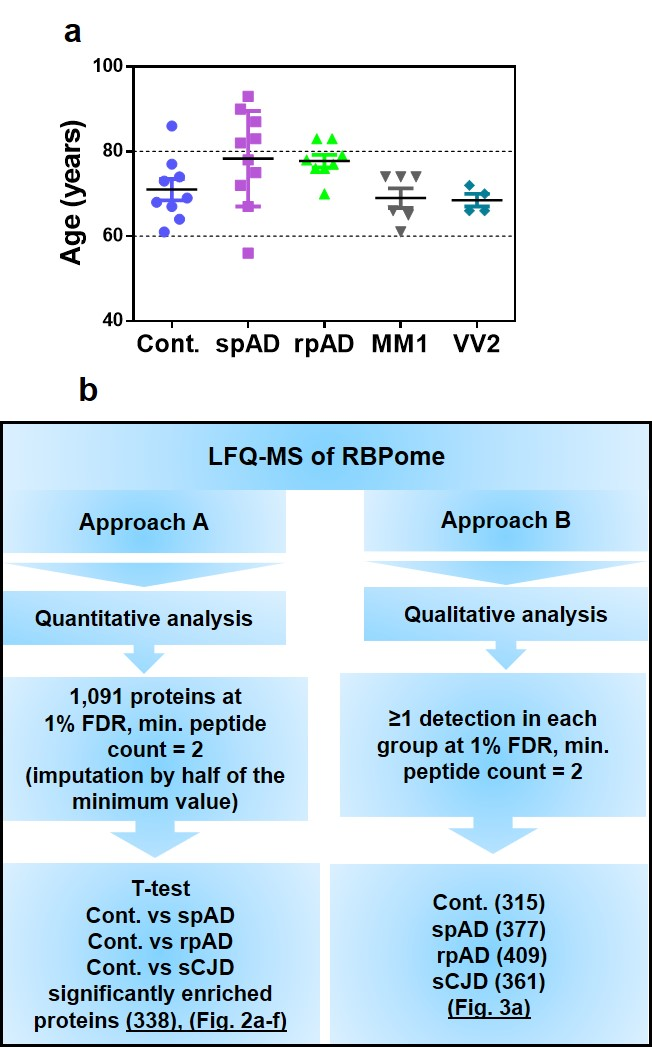

Supplement: Supplementary file 1 — Patient cohorts used in the present study. a Comparison of age distribution of the diseased and control cases used in the current study. b Statistical analysis of RBPome data: Two different approaches (A and B) were adopted fora detailed analysis of RBPome identified by label free quantification-MS, from the human brain frontal cortical region of 20 cases (spAD, rpAD, sCJD-MM1, sCJD-VV2 as well as controls). Approach A: Differential enrichment analysis of RBPome candidates by Perseus software. Zero values from the total spectral counts of the proteins were imputed by half of the minimum value. Pairwise t-test comparisons were performed between all the group combinations to identify significantly-enriched proteins in each group. Approach B: Qualitative analysis of the identified RBPome. Proteins with a single quantitative value were included in this approach from all the groups to have a broader impression of the isolated proteome. A comparative RBPome profile was obtained to find out common and unique proteins in all groups. Supplementary file1 (TIFF 558 kb) [file 401_2020_2178_MOESM1_ESM.tiff]

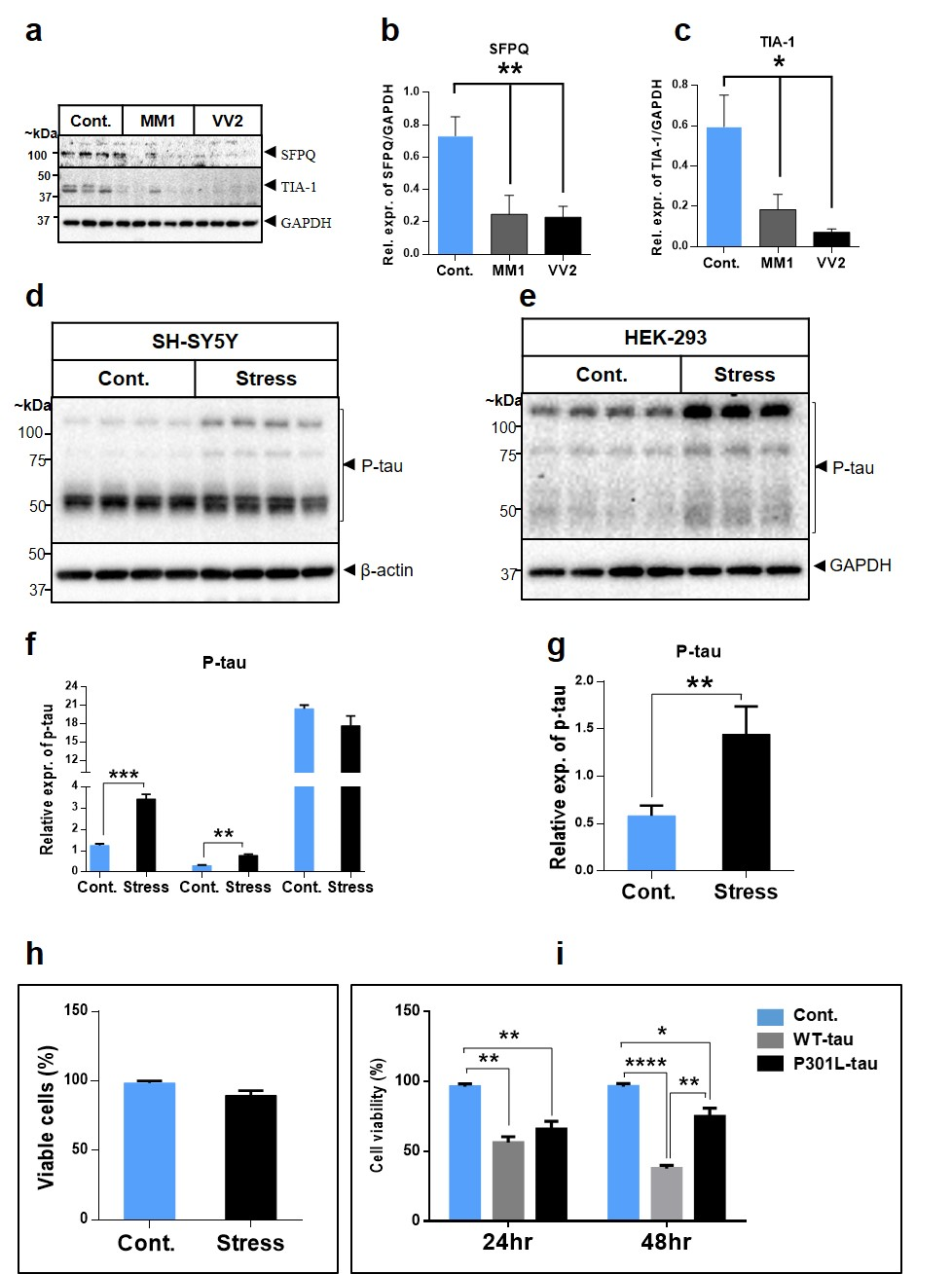

Supplement: Supplementary file 2 — Differential expression analysis of SFPQ and TIA-1. a Representative immunoblot images.Immunoblotting analysis was performed with frontal cortical human brain tissues from sCJD (-MM1 & -VV2 subtypes, n = 8) and non-demented controls (n = 8). b & c The densitometric analysis of SFPQ and TIA-1. One-way ANOVA was conducted,followed by Tukey post-hoc test for multiple comparisons. *p < 0.05, **p < 0.01. d-g Stress induced increase in tau phosphorylation in SH-SY5Y and HEK-293 cells. Representative immunoblots for p-tau in control (untreated) and stress (0.6mM NaASo2: 60 min) cells. Statistical significance was estimated by t-test **p < 0.01, ***p < 0.001. h Trypan-blue exclusion assay was used to estimate the cell viability after stress exposure. The percentage of viable cells was calculated in control(untreated) and stress cells. i The cell viability was measured by MTS assay, after expression of human-tau (both WT-tau and301L-tau) in comparison to controls at 24- and 48 hours post-transfection. One-way ANOVA followed by Tukey post-hoctest was used to calculate statistical significance, *p < 0.05, **p< 0.01, ***p < 0.001, ****p < 0.0001. Supplementary file2 (TIFF 543 kb) [file 401_2020_2178_MOESM2_ESM.tiff]

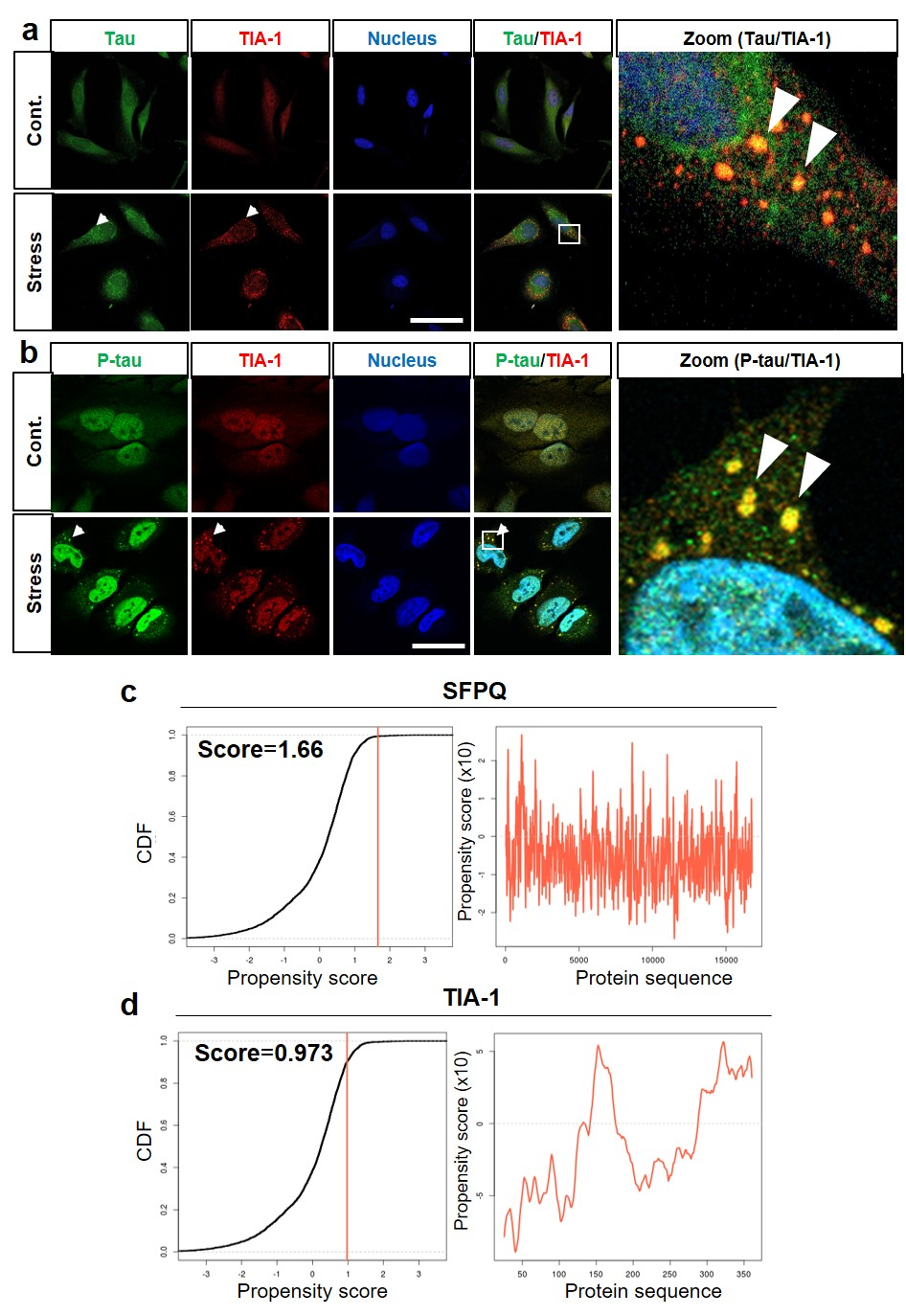

Supplement: Supplementary file 3 — Stress induces tau and p-tau positive stress granules. a & b Tau and p-tau are recruited into TIA-1-positive SGs. The cells were co-immuno-stained with primary antibodies specific for total-tau, p-tau and TIA-1. Cells were counterstained to visualize nuclei (blue), scale bar = 25 μm for a and 10 μm for b. c & d Liquid-liquid phase separation properties of SFPQ and TIA-1 were assessed by catGRANULES algorithm. Supplementary file3 (TIFF 1184 kb) [file 401_2020_2178_MOESM3_ESM.tiff]

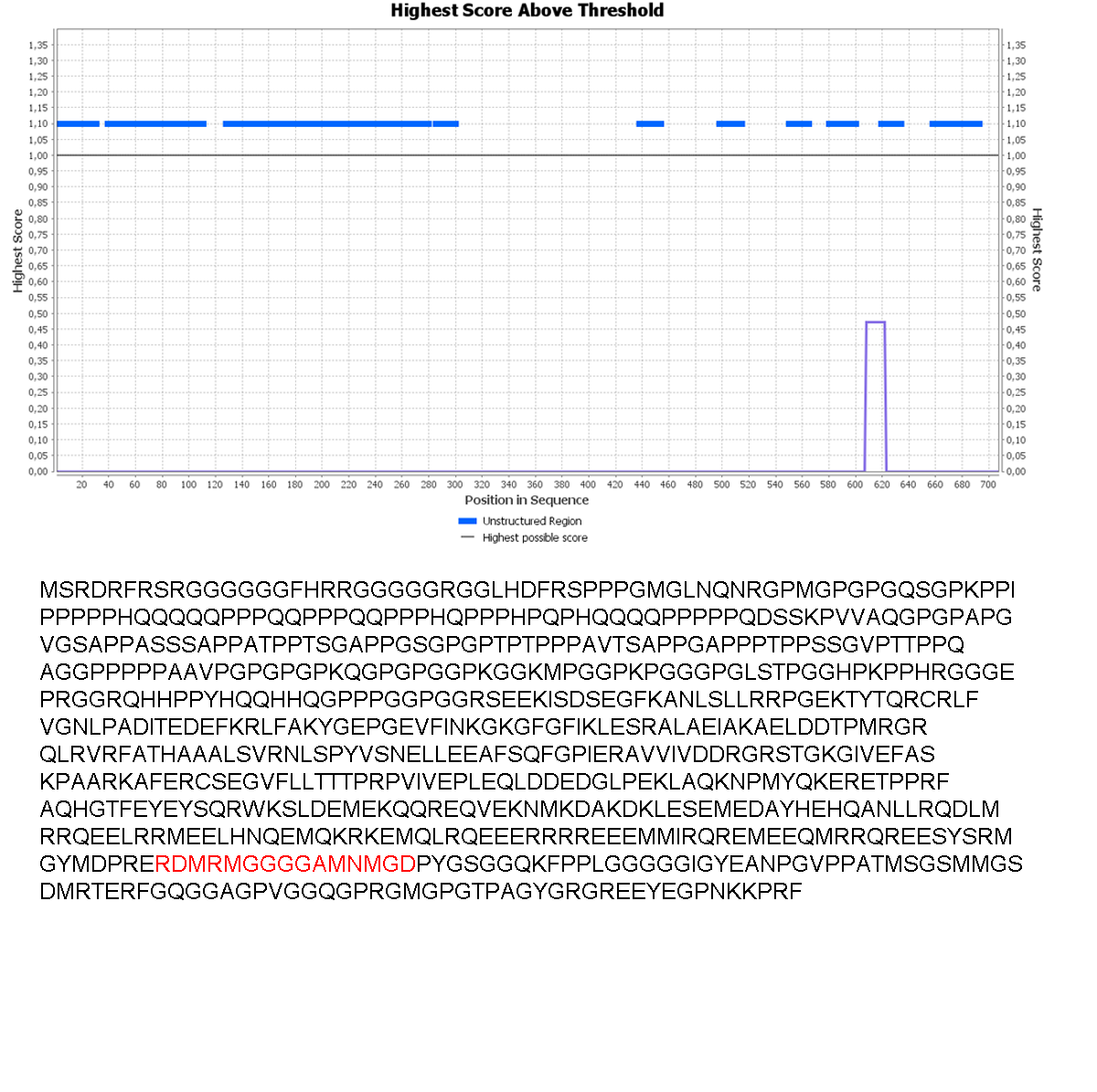

Supplement: Supplementary file 4 — Prediction of the presence of amyloidogenic region in the SFPQ protein according to the algorithm ArchCandy. The height of the column on the graph corresponds to probability of formation of the amyloid structure by the corresponding protein region. The amino acids in the amyloidogenic sequence of the protein are highlighted in red. Supplementary file4 (TIF 5095 kb) [file 401_2020_2178_MOESM4_ESM.tif]
