# Supplementary material for: SFPQ and Tau: critical factors contributing to rapid progression of Alzheimer’s disease
Source: Acta Neuropathol. 2020 Jun 23;140(3):317–39. doi: 10.1007/s00401-020-02178-y (PMC7423812; doi:10.1007/s00401-020-02178-y)
Supplement: Supplementary file 5 — Supplementary file5 (PDF 1158 kb) [file 401_2020_2178_MOESM5_ESM.pdf]

Supplemental Information:

SFPQ and Tau: critical factors contributing to rapid progression of Alzheimer's disease

Neelam Younas[1], Saima Zafar[1,2]*, Mohsin Shafiq[1,3], Aneeqa Noor[1], Anna Siegert[1], Amandeep Singh Arora[1,4], Alexey Galkin[5], Ayesha Zafar[3,6], Mathias Schmitz[1], Christine Stadelmann[7], Olivier Andreoletti[8], Isidre Ferrer[9,10,11,12], Inga Zerr[1]*

[1*] Department of Neurology, University Medical Center Göttingen and the German Center for Neurodegenerative Diseases (DZNE), Robert-Koch-Straße 40, 37075, Göttingen, Germany

[2] Biomedical Engineering and Sciences Department, School of Mechanical and Manufacturing Engineering (SMME), National University of Sciences and Technology (NUST), Islamabad, Pakistan

[3] Institute of Neuropathology, University Medical Center Hamburg-Eppendorf, Martinistraße 52, 20246, Hamburg, Germany

[4] Institute for Behavioral Medicine Research, 460 Medical Center Dr, Columbus, The Ohio State University, OH 43210, USA.

[5] St. Petersburg Branch, Vavilov Institute of General Genetics, St. Petersburg, Russia.

[6] College of Medicine Center for Pharmacogenomics, 1004 BRT, 460 W 12th Avenue, The Ohio State University Columbus, OH 43210, USA.

[7] Institute of Neuropathology, University Medical Center Göttingen, Germany.

[8] Institut National de la Recherche Agronomique/Ecole Nationale Vétérinaire, Toulouse, France.

[9] Department of Pathology and Experimental Therapeutics, University of Barcelona, Barcelona, Spain

[10] Bellvitge University Hospital-IDIBEL, Barcelona, Spain

[11] CIBERNED, Barcelona, Spain

[12] Hospitalet de Llobregat, Barcelona, Spain

* Correspondence to E-Mail: sz_awaan@yahoo.com, IngaZerr@med.uni-goettingen.de

2 Materials and methods

2.1 Quantitative real-time PCR (qRT-PCR)

cDNA was synthesized from brain-derived total RNA (1 µg), using High Capacity cDNA Reverse Transcription Kit (Thermo Fisher Scientific). All primer pairs for qRT-PCR assays were designed with Primer 3 (Table 1). Reaction mixtures for RT-PCR were prepared by mixing 1 uL of 10x PCR reaction buffer (Roche), 1 uL of dimethylsulphoxide (DMSO) (Sigma), 0.5 uL of 1:1000 dilutions of SYBR Green (Sigma), 0.2 mmol of each dNTP (Roche), 0.15 units of Taq-polymerase, 10 pm/uL of forward and reverse primers and 1 uL of cDNA (1:10 diluted); the volume was increased to 20 uL with RNAse-free water. Amplification was performed using Light Cycler 480 (Roche), with an initial denaturation at 95°C for 2 min, followed by 40-PCR cycles [(denaturation at 95°C for 30 seconds (sec), annealing at 56°C for 30 sec and extension at 72°C for 30 sec)]. Reactions were done in triplicates. No template controls were used to ensure reaction specificity. The data were analysed with Light Cycler 480 software SW1.5.1 (Roche) and values were normalised with GAPDH. The relative expression was calculated by comparative Ct method ($2^{\wedge -\Delta\Delta Ct}$) [45].

Table 1: List of primer pairs used in the study

Gene name	Direction	Sequence
GAPDH	*Forward*	TGGGTGTGAACCATGAGAAGTA
	Reverse	GAGTCCTTCCACGATACCAAAG
SFPQ	*Forward*	TGGGAAGTGACATGCGTACT
	Reverse	TGTTTGGGCCTTCGTACTCT
TIA-1	*Forward*	AGTTTCCTGGCCTGCATTTC
	Reverse	ACACTCGAGCTGTCTTTCCT

2.2 Immunoblotting

Frontal cortex tissues were lysed from spAD, rpAD, sCJD (-MM1 & -VV2 subtypes) and non-demented control subjects, along with cortical tissues from mouse brains, as published previously [91]. Equal amounts of proteins (30-50 ug) from each brain lysate or cell lysate were resolved by molecular weight on a 12% SDS-PAGE polyacrylamide gel (prepared in house) or 4-12% Bis-Tris gels (NuPAGE™ 4-12% Bis-Tris Protein Gels, Invitrogen). The protein marker (Precision plus protein dual color standards, Bio-Rad, Germany) was used to visualize the correct separation of the proteins and to confirm the correct protein band sizes. After separation on the gel, proteins were transferred onto a polyvinylidene difluoride (PVDF) membrane, with a 0.45 µm pore size, using a semi-dry blot chamber (Bio-Rad, Hercules, USA) for one hr. The membranes were blocked for 1 hr at RT in blocking reagent (5% non-fat dry milk in 1x TBS-T or PBS-T), followed by incubation with primary antibodies at 4°C overnight. All the primary antibodies (total-tau, phospho-tau (S199), SFPQ, TIA-1, β-actin, and GAPDH) were diluted in blocking buffer. Dilution of all the primary antibodies is given in table (see Table 2). Next, the membranes were washed in 1x PBS-T/TBS-T and incubated with secondary antibodies coupled to horseradish peroxidase (HRP) for 60 min at RT. Protein bands were detected using the enhanced chemiluminescent (ECL) method with Chemi-Doc (Bio-Rad). The densitometric analysis was performed with Image Lab software (3.0.1).

Table 2: List of primary antibodies used in the study

Primary Antibody	Origin	Dilution (IB)	Dilution (IF)	Cat. No./ Company
Tau-5	Mouse	1: 500	1: 100	ab80579/Abcam
Anti-tau (T22), oligomeric	Rabbit	1: 1000	1: 250	ABN454/Sigma-Aldrich
Phospho-tau (S199)	Rabbit	1: 1000	1: 100	ab81268/Abcam
TIA-1	Rabbit	1: 500	1: 100	ab140595/Abcam
TIA-1	Mouse	1: 500	-	ab40693/Abcam
TIA-1(G-3)	Rabbit	1: 200	1: 100	sc-166247/Santa Cruz
TIA-1 (G-3) AlexaFluor 488	Mouse	-	1: 50	sc-166247/Santa Cruz
SFPQ	Rabbit	1: 500	1: 100	ab38148/Abcam
GAPDH	Mouse	1: 3000	-	G8795/Sigma-Aldrich
β-Actin	Mouse	1: 1000	-	ab8227/Abcam

2.3 Immunocytochemistry

The cells were grown in T75 flasks with the culture medium (DMEM, 10% FBS, 1% PS). At confluency (70–90%), cells were trypsinized and seeded (5 x 10^4) on glass coverslips (13 mm) in 24 well plates. After undergoing the stress treatment as described above, the cells were fixed with 4% PFA for 20 min at RT, followed by 3x washes with ice-cold PBS for 5 min each. Permeabilization was achieved using 0.2% Triton X-100 in PBS for 10 min, followed by 3x washes with PBS for 5 min each. To avoid non-specific binding, cells were incubated with blocking buffer (1% BSA, 10% FBS in PBS) for 30 min at RT. Primary antibodies (in case of double labelling, both antibodies) were diluted in 1% BSA in PBS, followed by overnight incubation at 4°C. The cells were washed 3x with PBS (each wash was for 5 min) followed by incubation with secondary antibodies (AlexaFlour 488 and AlexaFlour 546) diluted in 1% BSA in PBS for two hrs at RT in the dark. The cells were washed 3x with PBS for 5 min each in the dark to remove the nonspecific binding. The cells were then counterstained with DAPI (one min) or a RedDot 2 Far red nuclear stain (20 min). After nuclear staining, the cells were washed 3x with PBS, followed by mounting with one drop of mounting medium (immuo-mount, Thermo Fisher Scientific). The slides were stored in the dark at -20°C or +4°C. Detail procedure for imaging the slides has been provided in the Co-immunoprecipitation protocol described in the main Manuscript. The average number of stress granules in each cell was calculated using FIJI software.

2.4 Cell lysis and protein extraction

Total protein extracts were prepared from 70-90% confluent HeLa, HEK-293 and SH-SY5Y cells. After stress treatment, all the cell lines were washed with 1x PBS, scraped and resuspended in lysis buffer [(50 mM Tris-HCl, pH 8, 1% Triton X-100, 0.5% CHAPS, 1mM DTT, protease, and phosphatase inhibitors (Roche, Germany)]. Lysates were sonicated using an ultrasonicator on ice, followed by incubation for one hr at 4°C with shaking. The lysates were centrifuged at 14000 rpm for 30 min at 4°C. The supernatants were transferred to new tubes. To harvest cells after transient transfections, cells were trypsinized and washed with 1x PBS followed by centrifugation at 400×g for 5 min at 4°C. The washed cell pellets were lysed, and protein quantification was performed with Bradford assay.

2.5 MTS assay

Cells were grown in T75 flasks with the culture medium (DMEM, 10% FBS, 1% PS). At confluency (70–90%), cells were trypsinized and then washed with 1x PBS, and seeded (10 x 10^3) in 96-well plates and incubated for 18-24 hours at 37°C. Transfections were performed with WT-tau and P301L-tau plasmids for variable periods of time (24 and 48 hours). To measure cell viability, MTS assay (ab197010, Abcam) was used according to the manufacturer's instructions. The culture media was replaced with a fresh medium before treatment with MTS [3-(4, 5-dimethylthiazol-2-yl)-5-(3-carboymethoxyphenyl)-2-(4-sulfophenyl)-2Htetrazolium, inner salt]. To estimate the effect of tau-expression on cell viability, reduced MTS tetrazolium complex (colored formazan product) was measured. This conversion is a property of metabolically active cells. For color development, cells were incubated at 37ºC for one hour and the absorbance measurement was taken at 490 nm using a Perkin Elmer Wallac 1420 Victor microplate reader (GMI, USA). Absorbance from the control wells (background) was subtracted from the experimental sample wells.

2.6 Trypan blue exclusion assay

Cell viability was also assessed through the trypan blue exclusion dye test. Briefly, the cell suspension in the culture medium (25 µL) after trypsinization was mixed with 25 µL of 0.4% trypan blue. After mixing, the 10 uL of cell suspension was loaded onto hemocytometer. Both viable (colorless) and dead (blue) cells were counted in each large square of the haemocytometer under (40x) objective for both untreated (control) and sodium arsenite treated (stress) cells.

2.7 SWATH-MS (Sequential Windowed Acquisition of All Theoretical Fragment Ion Mass Spectra) for global proteomics

2.7.1. Library preparation

Analytical-grade reagents were used for protein extraction and digestion. Ampuwa water (Ampuwa sterile water, Handels GmbH, Germany) was used to prepare all buffers and solutions. To prepare spectral peptide library, digested protein extracts (normalized for protein amounts) from each sample were pooled to a total amount of 220 µg and separated into fourteen staggered pooled fractions, using an Aekta pure (GE Healthcare) with a Hypersil Gold C18 column (Diam. 150x2,1 mm, Particle size: 3 µ). Digested proteins were analyzed on an Eksigent nanoLC425 nanoflow chromatography system associated with TripleTOF 5600$^+$, a hybrid triple quadrupole TOF mass spectrometer with a Nanospray III ionization source (Ionspray voltage 2400V, Interface heater temperature 150°C, Sheath gas setting 12). The peptides were dissolved in loading buffer (0.1% formic acid, 2% acetonitrile in optima water (Thermo Scientific) to a final concentration of 0.3 µg/µL and spiked with a synthetic peptide standard used for retention time alignment (iRT Standard, Schlieren, Schweiz). For every measurement, digested proteins (1.5 µg) were enriched on a precolumn (PharmFluidics µPAC Trapping Column) and separated on a PharmaFluidics µPAC micro Chip based seperations analytical column with 50 cm length) using a 120 min linear gradient of 5%-45% ACN, 0.1% FA) at a flow rate of 300 nL/min. Qualitative LC-MS/MS measurement was carried out using a Top 20 data-dependent acquisition (DDA) mode with a mass range of *m/z* 350–1250 for 250 milliseconds (ms). The resolution for data extraction was 30,000 full width at half maximum (FWHM). MS/MS scans of mass range *m/z* 180–1600 at a resolution of 17,500 FWHM for 85 ms and a precursor isolation width of 0.7 FWHM with a cycle time of 2.9 sec was used. For MS/MS, the criteria for precursor ions selection was: a

threshold intensity of more than 125 cps with charge states of 2+, 3+, and 4+, and the dynamic exclusion of 30 sec. In every reversed-phase fraction, two technical replicates were analyzed for the construction of the spectral library.

2.7.2 Quantitative SWATH measurement

For the SWATH measurement, MS/MS data was obtained using windows of 65 variable sizes across a mass range of 400-900 *m/z*. For each biological sample, two technical replicates were performed. Protein identifications were obtained using ProteinPilot Software version 5.0 build 4769 (AB Sciex) at "thorough" settings. MS/MS spectra were searched in the UniProtKB using *Homo sapiens* as reference proteome (revision 04/2018, 93661 entries) at a false discovery rate (FDR) of 1%. Spectral library and SWATH peak extraction were obtained in PeakView Software version 2.1 build 11041 (AB Sciex). Following retention time correction using the iRT standard, peak areas were extracted using information from the MS/MS library at 1% FDR. The peak areas were summed up to peptide and finally corresponding protein area values that were used for downstream statistical and functional analysis.

2.8 Bioinformatics Analysis

2.8.1 Differential enrichment analysis of RBPome

For a detailed analysis of the isolated RNA-binding proteomic candidates, two different approaches were used, here labeled approach A and B.

Approach A: to find out differentially enriched proteins in disease groups, pairwise t-tests (two-sided) were performed for all disease group combinations using Perseus software (version **1.5.0.31**). Missing or Zero values are observed in the data for several proteins in MS-based proteomics data. These values appear when the mass spectrometer cannot detect peptides having abundances below the censoring cutoff of the mass spectrometer. Such values are informative because they are below the lowest abundance observed for a peptide. In such situations, when quantitative values are missing in one group but are present in other groups, this more likely represents differences in abundance between groups, which might indicate interesting features specific to that group. Therefore, zero values were imputed by half of the minimum value of total spectrum count values, to have statistical analysis of the proteomic candidates (Approach A). Fold change (FC) for all comparisons was set at ±1.5 and a p-value < 0.05 for significance. Proteins which were identified as significantly enriched were used to make heatmaps using Perseus software (version **1.5.0.31).** Volcano plots were also calculated using Perseus software, where FC was log2-transformed, so that the data are centered on zero, while the p-values were transformed into −log10.

Approach B: given the exploratory nature of this discovery-based proteomics work-flow, the criteria were relaxed and proteins with a single quantitative value were included in the analysis to compare all the disease groups. To find similar and unique proteins, venn diagram was prepared using functional enrichment and analysis tool (FunRich).

2.8.2 Gene Ontology analysis and functional network mapping

To gain functional insights from proteomics data, three different enrichment strategies were used. Functional enrichment analysis was initially performed using Perseus software for significantly-enriched Gene Ontology

(GO) processes including the Biological Process and Molecular Function by Fisher's exact-test. Then, overrepresentation enrichment analysis was performed using the web-based Gene SeT Analysis Toolkit (WebGestalt), in the domains of Biological and Molecular Functions to have a GO Slim summary of enriched terms. Finally, an Ingenuity Pathway Analysis (IPA, Qiagen, USA) was performed to find out canonical pathways associated with up- and down-regulated proteins after tau-expression as described below.

2.8.3 Ingenuity Pathway Analysis (IPA)

The proteomic dataset containing the fold change and p-values of significantly regulated proteins was uploaded to IPA for core analysis (Qiagen, USA). The protein candidates from the submitted dataset generated top molecular networks based on Molecular and Biological Functions including canonical pathways, potential upstream regulators, and disease-based networks. The settings for analysis were based on direct and indirect relationships between differentially expressed proteins (DEPs), and were supported by experimentally reported data from human, mouse and rat studies [27]. Potential upstream regulators were designated as inhibited or activated according to the fold change and p-values (-log10-p-values) [65] of the DEPs.

Table 3: Prion-like domain prediction score of RBPs from PLAAC database

Experimental group	SEQid	COREscore	PAPAprop
**Cont., sCJD**	sp|Q12906|ILF3	30.688	0.092
**Cont. rpAD, sCJD**	sp|P08247|SYPH	25.009	-0.105
**Cont. spAD, sCJD**	sp|Q5D862|FILA2	24.633	0.109
**Cont., spAD, rpAD, sCJD**	sp|P17600|SYN1	20.311	-0.054
**Cont, sCJD**	sp|O43390|HNRPR	17.059	-0.049
**Cont., sCJD**	sp|P09012|SNRPA	11.165	-0.146
**spAD, rpAD**	sp|Q9UPA5|BSN	22.864	-0.044
**spAD, rpAD, sCJD**	sp|P20073|ANXA7	17.868	-0.033
**spAD, sCJD**	sp|Q92945|FUBP2	15.926	-0.065
**spAD, rpAD, sCJD**	sp|P02671|FIBA	12.12	0.033
**rpAD, sCJD**	sp|Q14103|HNRPD	30.124	0.164
**rpAD, sCJD**	sp|P23246|SFPQ	28.671	-0.1
**rpAD**	sp|P04156|PRIO	14.844	0.02
**sCJD**	sp|Q92734|TFG	38.015	-0.005
**sCJD**	sp|Q01844|EWS	34.368	0.057
**sCJD**	sp|P22626|ROA2	30.362	0.043
**sCJD**	sp|P09651|ROA1	28.381	0.093
**sCJD**	sp|Q8WUM4|PDC6	26.882	0.049
**sCJD**	sp|P50995|ANX11	19.803	-0.059

**sCJD**	sp|Q9UBV8|PEF1	19.787	0.005
**sCJD**	sp|P17931|LEG3	17.868	-0.033
**sCJD**	sp|P49840|GSK3A	11.251	-0.103
**sCJD**	sp|P14678|RSMB	10.958	-0.121
**sCJD**	sp|O60506|HNRPQ	8.799	-0.019

PLAAC: Prion-like amino acid composition, SEQid: sequence id from fasta file, COREscore: max sum of PLAAC LLRs, PAPAprop: max score of PAPA prion propensities [81], Cont.: control, spAD: sporadic Alzheimer's disease, rpAD: rapidly progressive Alzheimer's disease, sCJD: sporadic Creutzfeldt-Jakob disease.

Table 4: Summary of cases. rpAD: rapid Alzheimer disease; spAD: sporadic AD; Cont: control; CJD: Creutzfeldt-Jakob disease; N: normal; Braak NFT: Braak neurofibrillary tangle pathology (0-VI); TAP: Thal αB phase (1-5); CERAD: Consortium to Establish a Registry for Alzheimer disease (C0-C3); NIA score: National Institute on Aging (A0-A3); PMI: post-mortem delay. ABC categorization: A: TAP: amyloid score; B: Baak NFT pathology; C: CERAD (CERAD-NIA-AA score)

No.	Case	Clinical diagnosis	Age	Gender	Disease duration (y)	PMI (hr)	TAP	Braak NFT	CERAD-(NIA-AA score)	NIA-AA score
1	rpAD1	CJD	70	Male	< 4	11:30	3	VI	C (C3)	A2, B3, C3
2	rpAD2	CJD	76	Female	< 4	18	4	VI	C (C3)	A3, B3, C3
3	rpAD3	CJD	79	Female	< 4	05:30	3	V	C (C3)	A2, B3, C3
4	rpAD4	CJD	83	Male	< 4	05:30	5	VI	C (C3)	A3, B3, C3
5	rpAD5	CJD	83	Male	< 4	08:20	3	V	C (C3)	A2, B3, C3
6	rpAD6	CJD	76	Male	< 4	06:30	4	VI	C (C3)	A3, B3, C3
7	rpAD7	CJD	78	Male	< 4	03:30	5	VI	C (C3)	A3, B3, C3
8	spAD1	AD	78	Male	> 4	09:30	4	V	C (C3)	A3, B3, C3
9	spAD2	AD	72	Female	> 4	09:30	3	V	C (C3)	A2, B3, C3
10	spAD3	AD	82	Female	> 4	01:45	4	VI	C (C3)	A3, B3, C3
11	spAD4	AD-mixed dementia	56	Female	> 4	07	4	VI	C (C3)	A3, B3; C3
12	spAD5	AD	87	Male	> 4	07:05	3	V	C (C3)	A2, B3, C3
13	spAD6	AD-mixed dementia	75	Female	> 4	04:15	4	V	C (C3)	A3, B3, C3
14	spAD7	AD	93	Male	> 4	03	3	V	C (C3)	A2, B3, C3
15	spAD8	AD	74	Female	-	5:30	4	V	C (C3)	A3, B3, C3
16	spAD9	AD	82	Male	-	3:45	4	V	C (C3)	A3, B3, C3
17	Cont1	N	69	Male	-	03:45	1	II	A (C1)	A1, B1, C1

18	Cont2	N	68	Male	-	10:55	0	I	0 (C0)	A0, B1, C0
19	Cont.3	N	64	Male	-	08:35	0	I	0 (C0)	A0, B1, C0
20	Cont.4	N	67	Male	-	14:40	0	I	0 (C0)	A0, B1, C0
21	Cont.5	N	74	Male	-	05:30	1	II	A (C1)	A1, B1, C1
22	Cont.6	N	86	Male	-	05:30	1	II	0 (C1)	A1, B1, C1
23	Cont.7	N	73	Female	-	15:45	0	I	0 (C0)	A0, B1, C0
24	Cont.8	N	61	Male	-	04:30	0	I	0 (C0)	A0, B1, C0
25	Cont.9	N	77	Male	-	06:55	1	I	A (C1)	A1, B1, C1

Table 5: Details of sporadic Creutzfeldt Jakob disease subtype cases: neuropathological examination of all these cases was performed in the context of suspected prion disease in all cases. When PrP^{SC} positivity was observed, additional pathologies such as amyloid deposits were described briefly.

No.	Case	Age	Gender	Disease duration (y)	Genotype	PMI (hr)	Amyloid deposits
1	sCJD (MM1)1	65	Male	<1	MM/MV1	09:45	"Numerous amorphous amyloid deposits in the isocortex"
2	sCJD (MM1)2	74	Female	<1	MM/MV1	07:50	"Countless, mostly diffuse amyloid deposits in the isocotex and in the central gray matter"
3	sCJD (MM1)3	61	Male	<1	MM/MV1	07	"Diffuse amyloid deposits in frontal and occipital lobes"
4	sCJD (MM1)4	66	Female	<1	MM/MV1	05:05	"No other relevant pathological findings"
5	sCJD (MM1)5	74	Female	<1	MM/MV1	11	"Numerous amyloid beta deposits in the isocortex, and in the allocortex"
6	sCJD (MM1)6	74	Male	<1	MM/MV1	04:50	"Numerous amyloid deposits in the isocortex"
7	sCJD (VV2)1	66	Male	<1	VV2	15:30	"No other relevant pathological findings"
8	sCJD (VV2)2	70	Female	<1	VV2	11	"Few diffuse amyloid deposits in frontal lobes"
9	sCJD (VV2)3	72	Female	<1	VV2	06	"No other relevant pathological findings"
10	sCJD (VV2)4	66	Female	<1	VV2	04	"No other relevant pathological findings"

Table 6: Unique and common proteins identified in each group after RNA pull-down and mass spectrometry analysis

Disease Group	*IDs*	*Uniprot Acc. No.*	*Protein names*	*Involvement in disease*
spAD	**AEDO**	Q96SZ5	2-aminoethanethiol dioxygenase	
	**AL1L1**	O75891	Cytosolic 10-formyltetrahydrofolate dehydrogenase	
	**AL4A1**	P30038	Delta-1-pyrroline-5-carboxylate dehydrogenase, mitochondrial	Hyperprolinemia 2
	**AP2M1**	Q96CW1	AP-2 complex subunit mu	
	**ARP2**	P61160	Actin-related protein 2	
	**ARRB1**	P49407	Beta-arrestin-1	
	**ATPD**	P30049	ATP synthase subunit delta, mitochondrial	Mitochondrial complex V deficiency, nuclear type 5
	**CAD13**	P55290	Cadherin-13	
	**CADM4**	Q8NFZ8	Cell adhesion molecule 4	
	**CANB1**	P63098	Calcineurin subunit B type 1	
	**CAND1**	Q86VP6	Cullin-associated NEDD8-dissociated protein 1	
	**CAPZB**	P47756	F-actin-capping protein subunit beta	
	**CASA1**	P47710	Alpha-S1-casein [Cleaved into: Casoxin-D]	
	**CAZA1**	P52907	F-actin-capping protein subunit alpha-1	
	**CC50A**	Q9NV96	Cell cycle control protein 50A	
	**CD47**	Q08722	Leukocyte surface antigen CD47	
	**CLCA**	P09496	Clathrin light chain A	
	**CLH2**	P53675	Clathrin heavy chain 2	
	**CPLX2**	Q6PUV4	Complexin-2	
	**CSN2**	P61201	COP9 signalosome complex subunit 2	
	**CYFP2**	Q96F07	Cytoplasmic FMR1-interacting protein 2	Epileptic encephalopathy, early infantile, 65
	**DBNL**	Q9UJU6	Drebrin-like protein	
	**DC1L2**	O43237	Cytoplasmic dynein 1 light intermediate chain 2	
	**DCXR**	Q7Z4W1	L-xylulose reductase	Pentosuria
	**ECHA**	P40939	Trifunctional enzyme subunit alpha, mitochondrial	Mitochondrial trifunctional protein deficiency
	**ENOB**	P13929	Beta-enolase	Glycogen storage disease 13
	**FLNA**	P21333	Filamin-A	Periventricular nodular heterotopia 1
	**FLOT1**	O75955	Flotillin-1	
	**FUBP2**	Q92945	Far upstream element-binding protein 2	
	**GANAB**	Q14697	Neutral alpha-glucosidase AB	Polycystic kidney disease 3 with or without polycystic liver disease
	**GBB4**	Q9HAV0	Guanine nucleotide-binding protein subunit beta-4	Charcot-Marie-Tooth disease, dominant, intermediate type, F
	**GBG3**	P63215	Guanine nucleotide-binding protein G	
	**GDE**	P35573	Glycogen debranching enzyme	Glycogen storage disease 3
	**GLU2B**	P14314	Glucosidase 2 subunit beta	Polycystic liver disease 1 with or without kidney cysts

**GNAQ**	P50148	Guanine nucleotide-binding protein G	Capillary malformations, congenital
**GPD1L**	Q8N335	Glycerol-3-phosphate dehydrogenase 1-like protein	Brugada syndrome 2
**GPDM**	P43304	Glycerol-3-phosphate dehydrogenase, mitochondrial	
**HINT1**	P49773	Histidine triad nucleotide-binding protein 1	Neuromyotonia and axonal neuropathy, autosomal recessive
**HS74L**	O95757	Heat shock 70 kDa protein 4L	
**HSP76**	P17066	Heat shock 70 kDa protein 6	
**ICAM5**	Q9UMF0	Intercellular adhesion molecule 5	
**IDH3B**	O43837	Isocitrate dehydrogenase [NAD] subunit beta, mitochondrial	Retinitis pigmentosa 46
**IF4A1**	P60842	Eukaryotic initiation factor 4A-I	
**IF4B**	P23588	Eukaryotic translation initiation factor 4B	
**IPYR**	Q15181	Inorganic pyrophosphatase	
**KCC2A**	Q9UQM7	Calcium/calmodulin-dependent protein kinase type II subunit alpha	Mental retardation, autosomal dominant 53
**KCRM**	P06732	Creatine kinase M-type	
**KT3K**	Q9HA64	Ketosamine-3-kinase	
**L1CAM**	P32004	Neural cell adhesion molecule L1	Hydrocephalus due to stenosis of the aqueduct of Sylvius
**LASP1**	Q14847	LIM and SH3 domain protein 1	
**LIGO1**	Q96FE5	Leucine-rich repeat and immunoglobulin-like domain-containing nogo receptor-interacting protein 1	Mental retardation, autosomal recessive 64
**MAOM**	P23368	NAD-dependent malic enzyme, mitochondrial	
**MAP4**	P27816	Microtubule-associated protein 4	
**MK03**	P27361	Mitogen-activated protein kinase 3	
**MT1F**	P04733	Metallothionein-1F	
**NCHL1**	O00533	Neural cell adhesion molecule L1-like protein	
**NCKP1**	Q9Y2A7	Nck-associated protein 1	
**NCKX2**	Q9UI40	Sodium/potassium/calcium exchanger 2	
**NDRG1**	Q92597	Protein NDRG1	Charcot-Marie-Tooth disease 4D
**NEDD8**	Q15843	NEDD8	
**NEGR1**	Q7Z3B1	Neuronal growth regulator 1	
**NP1L4**	Q99733	Nucleosome assembly protein 1-like 4	
**NTRI**	Q9P121	Neurotrimin	
**ODPA**	P08559	Pyruvate dehydrogenase E1 component subunit alpha, somatic form, mitochondrial	Pyruvate dehydrogenase E1-alpha deficiency
**OLA1**	Q9NTK5	Obg-like ATPase 1	
**OPA1**	O60313	Dynamin-like 120 kDa protein, mitochondrial	Optic atrophy 1
**OPCM**	Q14982	Opioid-binding protein/cell adhesion molecule	Ovarian cancer
**PAK1**	Q13153	Serine/threonine-protein kinase PAK 1	Intellectual developmental disorder with macrocephaly, seizures, and speech delay

	**PAK3**	Q75914	Serine/threonine-protein kinase PAK 3	Mental retardation, X-linked 30
	**PCSK1**	Q9UHG2	ProSAAS	
	**PCY2**	Q99447	Ethanolamine-phosphate cytidylyltransferase	
	**PFKAL**	P17858	ATP-dependent 6-phosphofructokinase, liver type	
	**PFKAP**	Q01813	ATP-dependent 6-phosphofructokinase, platelet type	
	**PHIPL**	Q96FC7	Phytanoyl-CoA hydroxylase-interacting protein-like	
	**PLIN3**	O60664	Perilipin-3	
	**PPT1**	P50897	Palmitoyl-protein thioesterase 1	Ceroid lipofuscinosis, neuronal, 1
	**PTN11**	Q06124	Tyrosine-protein phosphatase non-receptor type 11	LEOPARD syndrome 1
	**QCR2**	P22695	Cytochrome b-c1 complex subunit 2, mitochondrial	Mitochondrial complex III deficiency, nuclear 5
	**RAB8B**	Q92930	Ras-related protein Rab-8B	
	**RALA**	P11233	Ras-related protein Ral-A	
	**RD23B**	P54727	UV excision repair protein RAD23 homolog B	
	**REEP5**	Q00765	Receptor expression-enhancing protein 5	
	**RHOC**	P08134	Rho-related GTP-binding protein RhoC	
	**SEMG1**	P04279	Semenogelin-1	
	**Septin-3**	Q9UH03	Neuronal-specific septin-3	
	**Septin-6**	Q14141	Septin-6	
	**Septin-9**	Q9UHD8	Septin-9	
	**SH3G1**	Q99961	Endophilin-A2	
	**SHLB2**	Q9NR46	Endophilin-B2	
	**SNAG**	Q99747	Gamma-soluble NSF attachment protein	
	**SPTN2**	O15020	Spectrin beta chain, non-erythrocytic 2	Spinocerebellar ataxia 5
	**SRC8**	Q14247	Src substrate cortactin	
	**SYNPO**	Q8N3V7	Synaptopodin	
	**TCAL5**	Q5H9L2	Transcription elongation factor A protein-like 5	
	**TCPB**	P78371	T-complex protein 1 subunit beta	
	**TCPQ**	P50990	T-complex protein 1 subunit theta	
	**TCTP**	P13693	Translationally-controlled tumor protein	
rpAD	**4F2**	P08195	4F2 cell-surface antigen heavy chain	
	**ACTA**	P62736	Actin, aortic smooth muscle	
	**ACY2**	P45381	Aspartoacylase	Canavan disease
	**ADDA**	P35611	Alpha-adducin	
	**ADT2**	P05141	ADP/ATP translocase 2	
	**AL7A1**	P49419	Alpha-aminoadipic semialdehyde dehydrogenase	Pyridoxine-dependent epilepsy
	**AMER2**	Q8N7J2	APC membrane recruitment protein 2	
	**AMPL**	P28838	Cytosol aminopeptidase	
	**ANK2**	Q01484	Ankyrin-2	Long QT syndrome 4
	**AOFA**	P21397	Amine oxidase [flavin-containing] A	Brunner syndrome
	**AOFB**	P27338	Amine oxidase [flavin-containing] B	
	**AP180**	O60641	Clathrin coat assembly protein AP180	
	**ASAH1**	Q13510	Acid ceramidase	Farber lipogranulomatosis

**AT2A2**	P16615	Sarcoplasmic/endoplasmic reticulum calcium ATPase 2	Acrokeratosis verruciformis
**ATP5H**	O75947	ATP synthase subunit d, mitochondrial	
**ATP5J**	P18859	ATP synthase-coupling factor 6, mitochondrial	
**ATP5L**	O75964	ATP synthase subunit g, mitochondrial	
**CALX**	P27824	Calnexin	
**CD44**	P16070	CD44 antigen	
**CDS2**	O95674	Phosphatidate cytidylyltransferase 2	
**CLD11**	O75508	Claudin-11	
**CMC1**	O75746	Calcium-binding mitochondrial carrier protein Aralar1	Epileptic encephalopathy, early infantile, 39
**CO4A**	P0C0L4	Complement C4-A	Complement component 4A deficiency
**CO4B**	P0C0L5	Complement C4-B	Systemic lupus erythematosus
**COX41**	P13073	Cytochrome c oxidase subunit 4 isoform 1, mitochondrial	
**CPNS1**	P04632	Calpain small subunit 1	
**CUTA**	O60888	Protein CutA	
**DC1I2**	Q13409	Cytoplasmic dynein 1 intermediate chain 2	
**DDTL**	A6NHG4	D-dopachrome decarboxylase-like protein	
**EF2**	P13639	Elongation factor 2	Spinocerebellar ataxia 26
**ERMIN**	Q8TAM6	Ermin	
**FIS1**	Q9Y3D6	Mitochondrial fission 1 protein	
**GBG2**	P59768	Guanine nucleotide-binding protein G	
**GHC1**	Q9H936	Mitochondrial glutamate carrier 1	Epileptic encephalopathy, early infantile, 3
**GPM6B**	Q13491	Neuronal membrane glycoprotein M6-b	
**HEBP1**	Q9NRV9	Heme-binding protein 1	
**HNRPD**	Q14103	Heterogeneous nuclear ribonucleoprotein D0	
**HYEP**	P07099	Epoxide hydrolase 1	
**IMB1**	Q14974	Importin subunit beta-1	
**LAMP1**	P11279	Lysosome-associated membrane glycoprotein 1	
**LANC2**	Q9NS86	LanC-like protein 2	
**MAG**	P20916	Myelin-associated glycoprotein	Spastic paraplegia 75, autosomal recessive
**MRP**	P49006	MARCKS-related protein	
**MTAP2**	P11137	Microtubule-associated protein 2	
**MTPN**	P58546	Myotrophin	
**NDKA**	P15531	Nucleoside diphosphate kinase A	
**NDUA4**	O00483	Cytochrome c oxidase subunit NDUFA4	Leigh syndrome
**ODO2**	P36957	Dihydrolipoyllysine-residue succinyltransferase component of 2-oxoglutarate dehydrogenase complex, mitochondrial	Platelet-activating factor acetylhydrolase IB subunit beta
**PA1B2**	P68402		
**PCBP2**	Q15366	Poly(rC)-binding protein 2	
**PGAM2**	P15259	Phosphoglycerate mutase 2	Glycogen storage disease 10
**PHB**	P35232	Prohibitin	

	**PHB2**	Q99623	Prohibitin-2	
	**PI42A**	P48426	Phosphatidylinositol 5-phosphate 4-kinase type-2 alpha	
	**PRIO**	P04156	Major prion protein	prion diseases, like: Creutzfeldt-Jakob disease
	**QCR1**	P31930	Cytochrome b-c1 complex subunit 1, mitochondrial	
	**QCR6**	P07919	Cytochrome b-c1 complex subunit 6, mitochondrial	
	**RAP1A**	P62834	Ras-related protein Rap-1A	
	**SCG1**	P05060	Secretogranin-1	
	**SCG2**	P13521	Secretogranin-2	
	**SDHA**	P31040	Succinate dehydrogenase [ubiquinone] flavoprotein subunit, mitochondrial	Mitochondrial complex II deficiency
	**Septin-4**	O43236	Septin-4	
	**SERA**	O43175	D-3-phosphoglycerate dehydrogenase	Phosphoglycerate dehydrogenase deficiency
	**SIRB1**	O00241	Signal-regulatory protein beta-1	
	**SNG3**	O43761	Synaptogyrin-3	
	**SV2A**	Q7L0J3	Synaptic vesicle glycoprotein 2A	
	**SV2B**	Q7L1I2	Synaptic vesicle glycoprotein 2B	
	**SYNJ1**	O43426	Synaptojanin-1	Parkinson disease 20, early-onset
	**TBA1B**	P68363	Tubulin alpha-1B chain	
	**TCPE**	P48643	T-complex protein 1 subunit epsilon	Neuropathy, hereditary sensory, with spastic paraplegia, autosomal recessive
	**TENA**	P24821	Tenascin	Deafness, autosomal dominant, 56
	**VA0D1**	P61421	V-type proton ATPase subunit d 1	
	**VAT1**	Q99536	Synaptic vesicle membrane protein VAT-1 homolog<br>Voltage-dependent anion-selective channel protein 1	Voltage-dependent anion-selective channel protein 2<br>Voltage-dependent anion-selective channel protein 3
	**VDAC1**	P21796		
	**VDAC2**	P45880		
	**VDAC3**	Q9Y277		
sCJD	**A2MG**	P01023	Alpha-2-macroglobulin	
	**AACT**	P01011	Alpha-1-antichymotrypsin	
	**ACO13**	Q9NPJ3	Acyl-coenzyme A thioesterase 13	
	**ADHX**	P11766	Alcohol dehydrogenase class-3	
	**AL1A1**	P00352	Retinal dehydrogenase 1	
	**ALDR**	P15121	Aldo-keto reductase family 1 member B1	
	**APEX1**	P27695	DNA-(apurinic or apyrimidinic site) lyase	
	**APOA1**	P02647	Apolipoprotein A-I	High density lipoprotein deficiency 2
	**ARP3**	P61158	Actin-related protein 3	
	**ASGL1**	Q7L266	Isoaspartyl peptidase/L-asparaginase	
	**BIEA**	P53004	Biliverdin reductase A	Hyperbiliverdinemia
	**CAB39**	Q9Y376	Calcium-binding protein 39	

**CATA**	P04040	Catalase	Acatalasemia
**CD59**	P13987	CD59 glycoprotein	Hemolytic anemia, CD59-mediated, with or without polyneuropathy
**CLIC4**	Q9Y696	Chloride intracellular channel protein 4	
**CMBL**	Q96DG6	Carboxymethylenebutenolidase homolog	
**CPPED**	Q9BRF8	Serine/threonine-protein phosphatase CPPED1	
**DOPD**	P30046	D-dopachrome decarboxylase	
**DYL2**	Q96FJ2	Dynein light chain 2, cytoplasmic	
**EFHD2**	Q96C19	EF-hand domain-containing protein D2	
**FABP7**	O15540	Fatty acid-binding protein, brain	
**FAHD1**	Q6P587	Acylpyruvase FAHD1, mitochondrial	
**FBX2**	Q9UK22	F-box only protein 2	
**FIBB**	P02675	Fibrinogen beta chain	Congenital afibrinogenemia
**FIBG**	P02679	Fibrinogen gamma chain	Congenital afibrinogenemia
**FKBP4**	Q02790	Peptidyl-prolyl cis-trans isomerase FKBP4	
**G6PD**	P11413	Glucose-6-phosphate 1-dehydrogenase	Anemia, non-spherocytic hemolytic, due to G6PD deficiency
**GBRL2**	P60520	Gamma-aminobutyric acid receptor-associated protein-like 2	
**GGCT**	O75223	Gamma-glutamylcyclotransferase	
**GNAI2**	P04899	Guanine nucleotide-binding protein G	
**GNPI1**	P46926	Glucosamine-6-phosphate isomerase 1	
**GSHB**	P48637	Glutathione synthetase	Glutathione synthetase deficiency
**HMGB1**	P09429	High mobility group protein B1	
**HNRPK**	P61978	Heterogeneous nuclear ribonucleoprotein K	Au-Kline syndrome
**IDHC**	O75874	Isocitrate dehydrogenase [NADP] cytoplasmic	Glioma
**IDHP**	P48735	Isocitrate dehydrogenase [NADP], mitochondrial	D-2-hydroxyglutaric aciduria 2
**IGLL5**	B9A064	Immunoglobulin lambda-like polypeptide 5	
**ILF2**	Q12905	Interleukin enhancer-binding factor 2	
**KAD3**	Q9UIJ7	GTP: AMP phosphotransferase AK3, mitochondrial	Phospholysine phosphohistidine inorganic pyrophosphate phosphatase
**LHPP**	Q9H008		
**LKHA4**	P09960	Leukotriene A-4 hydrolase	
**MPI**	P34949	Mannose-6-phosphate isomerase	Congenital disorder of glycosylation 1B
**MT2**	P02795	Metallothionein-2	
**NNRE**	Q8NCW5	NAD(P)H-hydrate epimerase	Encephalopathy, progressive, early-onset, with brain edema and/or leukoencephalopathy
**NQO2**	P16083	Ribosyldihydronicotinamide dehydrogenase	
**NUDT5**	Q9UKK9	ADP-sugar pyrophosphatase	
**PDCD6**	O75340	Programmed cell death protein 6	
**PITH1**	Q9GZP4	PITH domain-containing protein 1	

	**PROF2**	P35080	Profilin-2	
	**PSA1**	P25786	Proteasome subunit alpha type-1	
	**PSA2**	P25787	Proteasome subunit alpha type-2	
	**PSA4**	P25789	Proteasome subunit alpha type-4	
	**PSA5**	P28066	Proteasome subunit alpha type-5	
	**PSA6**	P60900	Proteasome subunit alpha type-6	
	**PSA7**	O14818	Proteasome subunit alpha type-7	
	**PSB1**	P20618	Proteasome subunit beta type-1	
	**PSB3**	P49720	Proteasome subunit beta type-3	
	**PSB5**	P28074	Proteasome subunit beta type-5	
	**PTGR1**	Q14914	Prostaglandin reductase 1	
	**RAB21**	Q9UL25	Ras-related protein Rab-21	
	**RAB5B**	P61020	Ras-related protein Rab-5B	
	**RASK**	P01116	GTPase KRas	Leukemia, acute myelogenous
	**RB11B**	Q15907	Ras-related protein Rab-11B	Neurodevelopmental disorder with ataxic gait, absent speech, and decreased cortical white matter
	**RHOA**	P61586	Transforming protein RhoA	
	**SH3L2**	Q9UJC5	SH3 domain-binding glutamic acid-rich-like protein 2	
	**SKP1**	P63208	S-phase kinase-associated protein 1	
	**SPB6**	P35237	Serpin B6	Deafness, autosomal recessive, 91
	**SYWC**	P23381	Tryptophan--tRNA ligase, cytoplasmic	Neuronopathy, distal hereditary motor, 9
	**TOLIP**	Q9H0E2	Toll-interacting protein	
	**UBC12**	P61081	NEDD8-conjugating enzyme Ubc12	
rpAD and sCJD			**Common between rpAD and SCJD**	
	**A1AG1**	P02763	Alpha-1-acid glycoprotein 1	
	**A1AT**	P01009	Alpha-1-antitrypsin	Alpha-1-antitrypsin deficiency
	**ACTN1**	P12814	Alpha-actinin-1	Bleeding disorder, platelet-type 15
	**ACYP2**	P14621	Acylphosphatase-2	
	**AK1A1**	P14550	Aldo-keto reductase family 1 member A1	
	**CD81**	P60033	CD81 antigen	Immunodeficiency, common variable, 6
	**CYTB**	P04080	Cystatin-B	Epilepsy, progressive myoclonic 1
	**DNJC5**	Q9H3Z4	DnaJ homolog subfamily C member 5	Ceroid lipofuscinosis, neuronal, 4B
	**FRIL**	P02792	Ferritin light chain	Hyperferritinemia with or without cataract
	**FSCN1**	Q16658	Fascin	
	**GLTP**	Q9NZD2	Glycolipid transfer protein	
	**GSTM2**	P28161	Glutathione S-transferase Mu 2	
	**HPLN1**	P10915	Hyaluronan and proteoglycan link protein 1	
	**LIS1**	P43034	Platelet-activating factor acetylhydrolase IB subunit alpha	Lissencephaly 1
	**OTUB1**	Q96FW1	Ubiquitin thioesterase OTUB1	
	**PDIA3**	P30101	Protein disulfide-isomerase A3	
	**PEA15**	Q15121	Astrocytic phosphoprotein PEA-15	

	**PIPNA**	Q00169	Phosphatidylinositol transfer protein alpha isoform	
	**PPIA**	P62937	Peptidyl-prolyl cis-trans isomerase A	
	**PTGDS**	P41222	Prostaglandin-H2 D-isomerase	
	**QOR**	Q08257	Quinone oxidoreductase	
	**RAB5C**	P51148	Ras-related protein Rab-5C	
	**RIDA**	P52758	2-iminobutanoate/2-iminopropanoate deaminase	
	**SCRN1**	Q12765	Secernin-1	
	**SERC**	Q9Y617	Phosphoserine aminotransferase	Phosphoserine aminotransferase deficiency
	**SFPQ**	P23246	Splicing factor, proline- and glutamine-rich	
	**SH3L1**	O75368	SH3 domain-binding glutamic acid-rich-like protein	
	**SNAA**	P54920	Alpha-soluble NSF attachment protein	
	**SNAB**	Q9H115	Beta-soluble NSF attachment protein	
	**SODM**	P04179	Superoxide dismutase [Mn], mitochondrial	Microvascular complications of diabetes 6
	**TKT**	P29401	Transketolase	Short stature, developmental delay, and congenital heart defects
	**TRFE**	P02787	Serotransferrin	
	**UGPA**	Q16851	UTP--glucose-1-phosphate uridylyltransferase	
	**VATH**	Q9UI12	V-type proton ATPase subunit H	
	**VCP**	P55072	Valosin containing protein	Inclusion body myopathy with early-onset Paget disease with or without frontotemporal dementia 1
spAD and sCJD			**Common between spAD and sCJD**	
		O43488	Aflatoxin B1 aldehyde reductase member 2	
	**CYBP**	Q9HB71	Calcyclin-binding protein	
	**DDAH2**	O95865	N(G), N(G)-dimethylarginine dimethylaminohydrolase 2	
	**ECH1**	Q13011	Delta(3,5)-Delta (2,4)-dienoyl-CoA isomeras, mitochondrial	
	**ENOPH**	Q9UHY7	Enolase-phosphatase E1	
	**FPPS**	P14324	Farnesyl pyrophosphate synthase	Porokeratosis 9, multiple types (POROK9)
	**GLO2**	Q16775	Hydroxyacylglutathione hydrolase, mitochondrial	
	**GLOD4**	Q9HC38	Glyoxalase domain-containing protein 4	
	**GMFB**	P60983	Glia maturation factor beta	
	**GSTM1**	P09488	Glutathione S-transferase Mu 1	
	**GSTM3**	P21266	Glutathione S-transferase Mu 3	
	**LGUL**	Q04760	Lactoylglutathione lyase	
	**LSAMP**	Q13449	Limbic system-associated membrane protein	
	**MK01**	P28482	Mitogen-activated protein kinase 1	
	**NCDN**	Q9UBB6	Neurochondrin	
	**PGM2L**	Q6PCE3	Glucose 1,6-bisphosphate synthase	

	**PP2BB**	P16298	Serine/threonine-protein phosphatase 2B catalytic subunit beta isoform	
	**RAB2A**	P61019	Ras-related protein Rab-2A	
	**RAB5A**	P20339	Ras-related protein Rab-5A	
	**TAGL**	Q01995	Transgelin	
spAD and rpAD			**Common between spAD and rpAD**	
	**AKA12**	Q02952	A-kinase anchor protein 12	
	**ANXA7**	P20073	Annexin A7	
	**APOE**	P02649	Apolipoprotein E	Hyperlipoproteinemia 3
	**AQP4**	P55087	Aquaporin-4	
	**AT2B1**	P20020	Plasma membrane calcium-transporting ATPase 1	
	**AT2B2**	Q01814	Plasma membrane calcium-transporting ATPase 2	
	**AT2B3**	Q16720	Plasma membrane calcium-transporting ATPase 3	Spinocerebellar ataxia, X-linked 1
	**AT2B4**	P23634	Plasma membrane calcium-transporting ATPase 4	
	**AT5F1**	P24539	ATP synthase F	
	**ATPG**	P36542	ATP synthase subunit gamma, mitochondrial	
	**BCAS1**	O75363	Breast carcinoma-amplified sequence 1	
	**BSN**	Q9UPA5	Protein bassoon	
	**CADM2**	Q8N3J6	Cell adhesion molecule 2	
	**CXA1**	P17302	Gap junction alpha-1 protein	Oculodentodigital dysplasia
	**EAA1**	P43003	Excitatory amino acid transporter 1	Episodic ataxia 6
	**EAA2**	P43004	Excitatory amino acid transporter 2	Epileptic encephalopathy, early infantile, 41
	**F10A1**	P50502	Hsc70-interacting protein	
	**FIBA**	P02671	Fibrinogen alpha chain	Congenital afibrinogenemia
	**HECAM**	Q14CZ8	Hepatocyte cell adhesion molecule	Leukoencephalopathy, megalencephalic, with subcortical cysts, 2A
	**MAON**	Q16798	NADP-dependent malic enzyme, mitochondrial	
	**MOG**	Q16653	Myelin-oligodendrocyte glycoprotein	Narcolepsy 7
	**MT1G**	P13640	Metallothionein-1G	
	**MT3**	P25713	Metallothionein-3	
	**NCAM2**	O15394	Neural cell adhesion molecule 2	
	**NCAN**	O14594	Neurocan core protein	
	**NHRF1**	O14745	Na (+)/H (+) exchange regulatory cofactor NHE-RF1	Nephrolithiasis/osteoporosis, hypophosphatemic, 2
	**NPTN**	Q9Y639	Neuroplastin	
	**NRCAM**	Q92823	Neuronal cell adhesion molecule	
	**OXR1**	Q8N573	Oxidation resistance protein 1	
	**PADI2**	Q9Y2J8	Protein-arginine deiminase type-2	
	**PALM**	O75781	Paralemmin-1	
	**PDIA6**	Q15084	Protein disulfide-isomerase A6	
	**PFKAM**	P08237	ATP-dependent 6-phosphofructokinase, muscle type	Glycogen storage disease 7

**PLCB1**	Q9NQ66	1-phosphatidylinositol 4,5-bisphosphate phosphodiesterase beta-1	Epileptic encephalopathy, early infantile, 12
**PRRT2**	Q7Z6L0	Proline-rich transmembrane protein 2	Episodic kinesigenic dyskinesia 1
**PTMA**	P06454	Prothymosin alpha	
**PTPRZ**	P23471	Receptor-type tyrosine-protein phosphatase zeta	
**RAB6B**	Q9NRW1	Ras-related protein Rab-6B	
**RTN1**	Q16799	Reticulon-1	
**RTN4**	Q9NQC3	Reticulon-4	
**S10AD**	Q99584	Protein S100-A13	
**SH3L3**	Q9H299	SH3 domain-binding glutamic acid-rich-like protein 3	
**SNG1**	O43759	Synaptogyrin-1	
**TENR**	Q92752	Tenascin-R	
**TYB4**	P62328	Thymosin beta-4	
**VPP1**	Q93050	V-type proton ATPase 116 kDa subunit a isoform 1	

Table 7: Tau-up regulated proteins: list of unique and common proteins (with their $-log_{10}$p-values), that were upregulated after expression of either WT-tau or P301L-tau

No.	UniProt ID	UniProt Accession	Protein names	(WT vs Cont.) (p-values)	(P301L vs Cont.) (p-values)
1	**IST1**	P53990	IST1 homolog	2.80404	
2	**XCT**	Q9UPY5	Cystine/glutamate transporter	2.70942	
3	**ITPR3**	Q14573	Inositol 1,4,5-trisphosphate receptor type 3	3.74147	
4	**CAD13**	P55290	Cadherin-13	3.29636	
5	**TMEDA**	P49755	Transmembrane emp24 domain-containing protein 10	2.79315	
6	**APC7**	Q9UJX3	Anaphase-promoting complex subunit 7	2.64649	
7	**PXDC2**	Q6UX71	Plexin domain-containing protein 2	2.60679	
8	**PLCA**	Q99943	1-acyl-sn-glycerol-3-phosphate acyltransferase alpha	2.77202	
9	**NUDC1**	Q96RS6-2	NudC domain-containing protein 1	2.58966	
10	**SYUG**	O76070	Gamma-synuclein	3.30288	
11	**A0A1W2PS43**	A0A1W2PS 43	Lysosome membrane protein 2	3.00432	
12	**F8VX04**	F8VX04	Sodium-coupled neutral amino acid transporter 1	3.15582	
13	**RAB18**	Q9NP72	Ras-related protein Rab-18	2.60603	
14	**Q5VZR0**	Q5VZR0	Golgi-associated plant pathogenesis-related protein 1	2.6549	
15	**CD44**	P16070	CD44 antigen	2.89138	

16	**COX2**	P00403	Cytochrome c oxidase subunit 2	2.89006	
17	**PYRG1**	P17812	CTP synthase 1	2.64826	
18	**C9JYN0**	C9JYN0	Synaptophysin-like protein 1	3.73999	
19	**E7ER44**	E7ER44	Lactotransferrin		5.61449
20	**K7ENL2**	K7ENL2	WW domain-binding protein 2		3.25965
21	**IF16**	Q16666	Gamma-interferon-inducible protein 16		2.54071
22	**A0A1B0GWC0**	A0A1B0GWC0	Carnitine O-palmitoyltransferase 2, mitochondrial		3.83877
23	**A0A087WX97**	A0A087WX97	Bcl-2-like protein 13		3.19665
24	**RND3**	P61587	Rho-related GTP-binding protein RhoE		3.65369
25	**PODXL**	O00592	Podocalyxin		5.52997
26	**MOT1**	P53985	Monocarboxylate transporter 1		4.65667
27	**CLIC4**	Q9Y696	Chloride intracellular channel protein 4		4.5989
28	**A0A075B730**	A0A075B730	Epiplakin		2.80249
29	**S38A2**	Q96QD8	Sodium-coupled neutral amino acid transporter 2		3.7117
30	**TPBG**	Q13641	Trophoblast glycoprotein		3.97711
31	**LAT1**	Q01650	Large neutral amino acids transporter small subunit 1		6.21328
32	**SQOR**	Q9Y6N5	Sulfide: quinone oxidoreductase, mitochondrial		3.5484
33	**E9PEB5**	E9PEB5	Far upstream element-binding protein 1		3.65134
34	**AAAT**	Q15758	Neutral amino acid transporter B		4.73081
35	**AHNK2**	Q8IVF2	Protein AHNAK2		2.93411
36	**RTN4**	Q9NQC3-2	Reticulon-4		2.78976
37	**AT1A1**	P05023-4	Sodium/potassium-transporting ATPase subunit alpha-1		2.80245
38	**A0A087X054**	A0A087X054	Hypoxia up-regulated protein 1		2.67896
39	**J3KPF3**	J3KPF3	4F2 cell-surface antigen heavy chain		3.40599
40	**VINC**	P18206	Vinculin		2.67223
41	**SYWC**	P23381-2	Tryptophan--tRNA ligase, cytoplasmic		2.87585
42	**UAP1**	Q16222	UDP-N-acetylhexosamine pyrophosphorylase		2.53642

43	**PLST**	P13797	Plastin-3		2.62095
			**Common in both WT- and P301L-tau expressing cells**		
44	**Tau**	P10636-6	Microtubule-associated protein tau	9.66209	9.99752
45	**STX12**	Q86Y82	Syntaxin-12	4.38935	3.90083
46	**A0A0A0MRJ7**	A0A0A0MRJ 7	Coagulation factor V	2.79996	2.55125
47	**TSP1**	P07996	Thrombospondin-1	4.63034	3.69623
48	**STOM**	P27105	Erythrocyte band 7 integral membrane protein	2.63921	4.70864
49	**AT1B1**	P05026	Sodium/potassium-transporting ATPase subunit beta-1	4.36966	4.04487
50	**SNG2**	O43760	Synaptogyrin-2	3.38169	3.37281
51	**SODM**	P04179	Superoxide dismutase [Mn], mitochondrial	4.35392	4.21758
52	**E9PR17**	E9PR17	CD59 glycoprotein	4.06777	5.10992
53	**MOT4**	O15427	Monocarboxylate transporter 4	3.57067	3.15312
54	**RAI3**	Q8NFJ5	Retinoic acid-induced protein 3	5.11684	3.4051
55	**RTN3**	O95197-3	Reticulon-3	2.89615	2.83528
56	**FLNB**	O75369-8	Filamin-B	4.65931	4.00561
57	**QCR1**	P31930	Cytochrome b-c1 complex subunit 1, mitochondrial	3.85388	5.56346
58	**B4DKB2**	B4DKB2	Endothelin-converting enzyme 1	2.89353	2.60173
59	**VDAC2**	P45880	Voltage-dependent anion-selective channel protein 2	3.02634	3.84069
60	**ANXA3**	P12429	Annexin A3	2.89038	4.15502
61	**BIP**	P11021	Endoplasmic reticulum chaperone BiP	4.09375	5.35755
62	**ANXA4**	P09525	Annexin A4	4.25635	3.6306
63	**A0A0G2JIW1**	A0A0G2JIW 1	Heat shock 70 kDa protein 1B	3.97859	2.54103

Table 8: Tau down-regulated proteins: list of unique and common proteins (with their -$\log_{10}$p-values), that were down-regulated after expression of either WT-tau or P301L-tau

No.	**UniProt ID**	**UniProt Accession**	**Protein names**	**WT vs Cont. (p-values)**	**P301L vs Cont. (p-values)**
1	**CAPZB**	P47756-2	F-actin-capping protein subunit beta	2.90632	
2	**CLIC1**	O00299	Chloride intracellular channel protein 1	2.74343	
3	**1433E**	P62258	14-3-3 protein epsilon	2.97992	
4	**PDLI7**	Q9NR12	PDZ and LIM domain protein 7	2.87187	
5	**DHPR**	P09417	Dihydropteridine reductase	3.29976	
6	**TBB4B**	P68371	Tubulin beta-4B chain	2.61161	

7	**CYBP**	Q9HB71	Calcyclin-binding protein	4.20659
8	**GSH0**	P48507	Glutamate--cysteine ligase regulatory subunit	3.64493
9	**IDHC**	O75874	Isocitrate dehydrogenase [NADP] cytoplasmic	3.18656
10	**CRK**	P46108	Adapter molecule crk	2.66295
11	**LKHA4**	P09960	Leukotriene A-4 hydrolase	3.07686
12	**LDHA**	P00338	L-lactate dehydrogenase A chain	2.60496
13	**COF1**	P23528	Cofilin-1	2.86394
14	**PSME3**	P61289	Proteasome activator complex subunit 3	2.59093
15	**XPO6**	Q96QU8	Exportin-6	2.63751
16	**PFKAM**	P08237	ATP-dependent 6-phosphofructokinase, muscle type	2.77411
17	**METK2**	P31153	S-adenosylmethionine synthase isoform type-2	2.59551
18	**I3L0H8**	I3L0H8	ATP-dependent RNA helicase DDX19A	3.00712
19	**A0A087WYT3**	A0A087WYT3	Prostaglandin E synthase 3	6.25017
20	**MBB1A**	Q9BQG0	Myb-binding protein 1A	3.31109
21	**GANAB**	Q14697-2	Neutral alpha-glucosidase AB	3.99331
22	**RPAC1**	O15160	DNA-directed RNA polymerases I and III subunit RPAC1	2.95675
23	**6PGD**	P52209	6-phosphogluconate dehydrogenase, decarboxylating	3.15286
24	**F2Z2Y4**	F2Z2Y4	Pyridoxal kinase	3.349
25	**KTHY**	P23919	Thymidylate kinase	4.05072
26	**ACADM**	P11310-2	Medium-chain specific acyl-CoA dehydrogenase, mitochondrial	3.37726
27	**RT27**	Q92552	28S ribosomal protein S27, mitochondrial	2.65651
28	**A0A1B0GW77**	A0A1B0GW77	Alpha-aminoadipic semialdehyde dehydrogenase	2.57993
29	**IPYR**	Q15181	Inorganic pyrophosphatase	2.56092
30	**KPYM**	P14618	Pyruvate kinase PKM	3.3471
31	**TWF1**	Q12792	Twinfilin-1	3.16219
32	**MCM6**	Q14566	DNA replication licensing factor MCM6	2.98343
33	**I3L2B0**	I3L2B0	Clustered mitochondria protein homolog	3.14353
34	**KPRA**	Q14558	Phosphoribosyl pyrophosphate synthase-associated protein 1	2.88641
35	**ERO1A**	Q96HE7	ERO1-like protein alpha	3.48398
36	**AP3D1**	O14617	AP-3 complex subunit delta-1	2.64148
37	**Q5QPM7**	Q5QPM7	Proteasome inhibitor PI31 subunit	2.779
38	**TNPO3**	Q9Y5L0	Transportin-3	3.19029
39	**NAT10**	Q9H0A0	RNA cytidine acetyltransferase	2.60558
40	**F6WQW2**	F6WQW2	Ran-specific GTPase-activating protein	2.6097
41	**HAT1**	O14929	Histone acetyltransferase type B catalytic subunit	2.61858
42	**E7ESZ7**	E7ESZ7	NADH dehydrogenase [ubiquinone] 1 alpha subcomplex subunit 10, mitochondrial	3.34015
43	**A0A1B0GWE8**	A0A1B0GWE8	Cathepsin D	3.59333
44	**APEX1**	P27695	DNA-(apurinic or apyrimidinic site) lyase	2.573

45	**LNP**	Q9C0E8	Endoplasmic reticulum junction formation protein lunapark	3.024
46	**PARK7**	Q99497	Protein/nucleic acid deglycase DJ-1	3.53845
47	**PP2AA**	P67775	Serine/threonine-protein phosphatase 2A catalytic subunit alpha isoform	2.85783
48	**DUS23**	Q9BVJ7	Dual specificity protein phosphatase 23	2.6001
49	**NUP43**	Q8NFH3	Nucleoporin Nup43	2.92109
50	**TF3C4**	Q9UKN8	General transcription factor 3C polypeptide 4	2.85149
51	**UBA3**	Q8TBC4	NEDD8-activating enzyme E1 catalytic subunit	2.85788
52	**B1AH49**	B1AH49	3-mercaptopyruvate sulfurtransferase	4.16312
53	**ABCD3**	P28288	ATP-binding cassette sub-family D member 3	2.89151
54	**Q5QPR3**	Q5QPR3	Cyclin-dependent kinase 11A	3.07425
55	**D6RG13**	D6RG13	40S ribosomal protein S3a	3.40351
56	**B7Z4B8**	B7Z4B8	Heterogeneous nuclear ribonucleoprotein U-like protein 1	3.80247
57	**AN32E**	Q9BTT0	Acidic leucine-rich nuclear phosphoprotein 32 family member E	3.0431
58	**HPBP1**	Q9NZL4	Hsp70-binding protein 1	3.14118
59	**PAIRB**	Q8NC51	Plasminogen activator inhibitor 1 RNA-binding protein	2.72168
60	**CDK1**	P06493	Cyclin-dependent kinase 1	6.03772
61	**X6RLT1**	X6RLT1	Negative elongation factor C/D	2.93622
62	**TNAP2**	Q03169	Tumor necrosis factor alpha-induced protein 2	2.62938
63	**MLKL**	Q8NB16	Mixed lineage kinase domain-like protein	3.39344
64	**E7ETK0**	E7ETK0	40S ribosomal protein S24	3.59647
65	**DHX36**	Q9H2U1	ATP-dependent DNA/RNA helicase DHX36	3.02912
66	**TBCC**	Q15814	Tubulin-specific chaperone C	2.65875
67	**RL6**	Q02878	60S ribosomal protein L6	3.71789
68	**TOM34**	Q15785	Mitochondrial import receptor subunit TOM34	3.64649
69	**HP1B3**	Q5SSJ5	Heterochromatin protein 1-binding protein 3	3.88668
70	**C19L1**	Q69YN2	CWF19-like protein 1	2.65963
71	**RS17**	P08708	40S ribosomal protein S17	3.74528
72	**E7ESA6**	E7ESA6	Focal adhesion kinase 1	2.64713
73	**AP3M1**	Q9Y2T2	AP-3 complex subunit mu-1	2.75333
74	**M0QXD6**	M0QXD6	General transcription factor IIF subunit 1	2.56235
75	**WNT5A**	P41221	Protein Wnt-5a	2.59575
76	**GMDS**	O60547	GDP-mannose 4,6 dehydratase	3.08654
77	**I3L0X5**	I3L0X5	Sperm-associated antigen 7	3.3113
78	**E9PH64**	E9PH64	NADH dehydrogenase [ubiquinone] 1 beta subcomplex subunit 9	2.95705
79	**MIPEP**	Q99797	Mitochondrial intermediate peptidase	2.7807
80	**A0A087WZR9**	A0A087WZR9	Pyrroline-5-carboxylate reductase	5.26058
81	**CHIP**	Q9UNE7	E3 ubiquitin-protein ligase CHIP	2.93915

82	**TBCE**	Q15813	Tubulin-specific chaperone E	2.57258	
83	**SAAL1**	Q96ER3	Protein SAAL1	2.84296	
84	**SYRC**	P54136	Arginine--tRNA ligase, cytoplasmic		3.68764
85	**HTAI2**	Q9BUP3	Oxidoreductase HTATIP2		2.52883
86	**GRP75**	P38646	Stress-70 protein, mitochondrial		3.35915
87	**PSB3**	P49720	Proteasome subunit beta type-3		2.82347
88	**GSTP1**	P09211	Glutathione S-transferase P		3.25767
89	**RAB7A**	P51149	Ras-related protein Rab-7a		2.95429
90	**TCPG**	P49368	T-complex protein 1 subunit gamma		3.5822
91	**RAB9A**	P51151	Ras-related protein Rab-9A		2.60002
92	**PHB**	P35232	Prohibitin		2.76765
93	**RS27A**	P62979	Ubiquitin-40S ribosomal protein S27a		3.44529
94	**E7EQR4**	E7EQR4	Ezrin		2.70739
95	**RIR1**	P23921	Ribonucleoside-diphosphate reductase large subunit		2.55098
96	**J3QQT2**	J3QQT2	60S ribosomal protein L17		2.99247
97	**SRP68**	Q9UHB9	Signal recognition particle subunit SRP68		3.01785
98	**SMD3**	P62318	Small nuclear ribonucleoprotein Sm D3		3.12363
99	**SYQ**	P47897	Glutamine--tRNA ligase		2.65577
100	**H3BQI1**	H3BQI1	Dynein light chain roadblock-type 2		2.72107
101	**MCM5**	P33992	DNA replication licensing factor MCM5		2.94611
102	**Q8WVC2**	Q8WVC2	40S ribosomal protein S21		2.76124
103	**NDUS3**	O75489	NADH dehydrogenase [ubiquinone] iron-sulfur protein 3, mitochondrial		3.63337
104	**RL7**	P18124	60S ribosomal protein L7		2.73981
105	**C9JA28**	C9JA28	Translocon-associated protein subunit gamma		3.0665
106	**S4R3E9**	S4R3E9	NEDD8-MDP1 readthrough		3.09505
107	**VIME**	P08670	Vimentin		3.24861
108	**I3L504**	I3L504	Eukaryotic translation initiation factor 5A-1		2.65408
109	**TM109**	Q9BVC6	Transmembrane protein 109		3.52926
110	**DRG1**	Q9Y295	Developmentally-regulated GTP-binding protein 1		3.14037
111	**SMD1**	P62314	Small nuclear ribonucleoprotein Sm D1		3.54617
112	**H0YEN5**	H0YEN5	40S ribosomal protein S2		3.76272
113	**MCM3**	P25205-2	DNA replication licensing factor MCM3		3.01339
114	**ARF6**	P62330	ADP-ribosylation factor 6		3.18205
115	**RL7A**	P62424	60S ribosomal protein L7a		3.56152
116	**BZW2**	Q9Y6E2	Basic leucine zipper and W2 domain-containing protein 2		4.20893
117	**A0A0A0MTN0**	A0A0A0MTN0	Cullin-2		2.85465
118	**THOP1**	P52888	Thimet oligopeptidase		3.49956
119	**J3QRI7**	J3QRI7	60S ribosomal protein L26		5.03306
120	**ATPO**	P48047	ATP synthase subunit O, mitochondrial		2.73623
121	**LSM3**	P62310	U6 snRNA-associated Sm-like protein LSm3		3.12073

122	**SRP14**	P37108	Signal recognition particle 14 kDa protein	2.97832
123	**NCBP2**	P52298	Nuclear cap-binding protein subunit 2	2.55743
124	**RAN**	P62826	GTP-binding nuclear protein Ran	2.58877
125	**J3KQ48**	J3KQ48	Peptidyl-tRNA hydrolase 2, mitochondrial	4.00593
126	**TXD17**	Q9BRA2	Thioredoxin domain-containing protein 17	3.25391
127	**MGST1**	P10620	Microsomal glutathione S-transferase 1	2.82746
128	**SNX1**	Q13596	Sorting nexin-1	2.5274
129	**HNRPF**	P52597	Heterogeneous nuclear ribonucleoprotein F	2.89938
130	**RL13**	P26373	60S ribosomal protein L13	3.9096
131	**A0A087WUD3**	A0A087WUD3	Oligosaccharyltransferase complex subunit OSTC	2.65514
132	**RS28**	P62857	40S ribosomal protein S28	5.78257
133	**J3KTA4**	J3KTA4	Probable ATP-dependent RNA helicase DDX5	4.43944
134	**DDX6**	P26196	Probable ATP-dependent RNA helicase DDX6	2.70974
135	**ZC3HF**	Q8WU90	Zinc finger CCCH domain-containing protein 15	2.64774
136	**RM21**	Q7Z2W9	39S ribosomal protein L21, mitochondrial	3.18026
137	**ULA1**	Q13564	NEDD8-activating enzyme E1 regulatory subunit	3.12154
138	**RL11**	P62913	60S ribosomal protein L11	4.33191
139	**RS11**	P62280	40S ribosomal protein S11	3.57281
140	**RS8**	P62241	40S ribosomal protein S8	4.43922
141	**RL21**	P46778	60S ribosomal protein L21	3.30215
142	**A0A087X0X3**	A0A087X0X3	Heterogeneous nuclear ribonucleoprotein M	2.92309
143	**RS23**	P62266	40S ribosomal protein S23	4.45084
144	**ATD3A**	Q9NVI7-2	ATPase family AAA domain-containing protein 3A	2.65768
145	**RL23**	P62829	60S ribosomal protein L23	4.39359
146	**2A5D**	Q14738-3	Serine/threonine-protein phosphatase 2A 56 kDa regulatory subunit delta isoform	3.24429
147	**RS13**	P62277	40S ribosomal protein S13	5.13601
148	**G5E9Q6**	G5E9Q6	Profilin	3.91099
149	**RL12**	P30050	60S ribosomal protein L12	4.5988
150	**XPO5**	Q9HAV4	Exportin-5	2.81304
151	**DDX23**	Q9BUQ8	Probable ATP-dependent RNA helicase DDX23	3.09004
152	**RL27**	P61353	60S ribosomal protein L27	5.56322
153	**E5RI99**	E5RI99	60S ribosomal protein L30	4.79294
154	**SHIP2**	O15357	Phosphatidylinositol 3,4,5-trisphosphate 5-phosphatase 2	2.93228
155	**PRP6**	O94906	Pre-mRNA-processing factor 6	4.71226
156	**SYDM**	Q6PI48	Aspartate--tRNA ligase, mitochondrial	4.19994
157	**KIF2A**	O00139-2	Kinesin-like protein KIF2A	2.70644
158	**J3QR09**	J3QR09	Ribosomal protein L19	2.74218
159	**OCAD2**	Q56VL3	OCIA domain-containing protein 2	4.79686
160	**J3QSV6**	J3QSV6	Ribosomal L1 domain-containing protein 1	4.28401
161	**RL38**	P63173	60S ribosomal protein L38	2.93381

162	**A0A087X2D0**	A0A087X2D0	Serine/arginine-rich-splicing factor 3		4.36894
163	**GPI8**	Q92643	GPI-anchor transamidase		2.86273
164	**NU160**	Q12769	Nuclear pore complex protein Nup160		2.76185
165	**SMYD3**	Q9H7B4	Histone-lysine N-methyltransferase SMYD3		3.32229
166	**YMEL1**	Q96TA2	ATP-dependent zinc metalloprotease YME1L1		2.84011
167	**PDCD4**	Q53EL6	Programmed cell death protein 4		3.59557
168	**TMED1**	Q13445	Transmembrane emp24 domain-containing protein 1		2.72926
169	**RCN2**	Q14257	Reticulocalbin-2		3.22922
170	**R39L5**	Q59GN2	Putative 60S ribosomal protein L39-like 5		5.63947
171	**SRP09**	P49458	Signal recognition particle 9 kDa protein		4.54385
172	**TSR1**	Q2NL82	Pre-rRNA-processing protein TSR1 homolog		2.96676
173	**DYR**	P00374	Dihydrofolate reductase		4.23334
174	**DHX8**	Q14562	ATP-dependent RNA helicase DHX8		2.56375
175	**RIPK1**	Q13546	Receptor-interacting serine/threonine-protein kinase 1		3.70312
Common in both WT- and P301L- tau expressing cells					
176	**F8WCF6**	F8WCF6	Actin-related protein 2/3 complex subunit 4	3.33715	4.14691
177	**RACK1**	P63244	Receptor of activated protein C kinase 1	3.39824	4.51018
178	**SYDC**	P14868	Aspartate--tRNA ligase, cytoplasmic	2.74959	2.57307
179	**EIF3K**	Q9UBQ5	Eukaryotic translation initiation factor 3 subunit K	2.81828	4.58194
180	**H9KV45**	H9KV45	Ubiquitin-conjugating enzyme E2 D3	2.67823	5.42209
181	**Q5JR08**	Q5JR08	Rho-related GTP-binding protein RhoC	3.44748	3.81268
182	**PROF1**	P07737	Profilin-1	3.07127	4.69295
183	**FPPS**	P14324	Farnesyl pyrophosphate synthase	3.80014	3.24431
184	**Q5VV89**	Q5VV89	Microsomal glutathione S-transferase 3	3.6279	4.42465
185	**SYK**	Q15046	Lysine--tRNA ligase	3.9661	3.1659
186	**PUR2**	P22102	Trifunctional purine biosynthetic protein adenosine-3 [Includes: Phosphoribosylamine--glycine ligase	2.81901	2.93211
187	**SND1**	Q7KZF4	Staphylococcal nuclease domain-containing protein 1	3.019	3.608
188	**C9JZR2**	C9JZR2	Catenin delta-1	2.6207	2.99525
189	**CAN2**	P17655	Calpain-2 catalytic subunit	3.42537	3.11891
190	**AAAS**	Q9NRG9	Aladin	4.28764	4.41039
191	**SYLC**	Q9P2J5	Leucine--tRNA ligase, cytoplasmic	3.39758	3.6911
192	**RNZ2**	Q9BQ52	Zinc phosphodiesterase ELAC protein 2	3.34161	3.22
193	**TIF1B**	Q13263	Transcription intermediary factor 1-beta	3.23108	3.44094
194	**EIF3B**	P55884	Eukaryotic translation initiation factor 3 subunit B	4.32237	4.51386
195	**PPIA**	P62937	Peptidyl-prolyl cis-trans isomerase A	3.8054	4.17
196	**H0YAK1**	H0YAK1	G-rich sequence factor 1	3.46677	2.53854

197	**EIF3G**	O75821	Eukaryotic translation initiation factor 3 subunit G	2.72457	3.04545
198	**Q32Q12**	Q32Q12	Nucleoside diphosphate kinase	3.00854	3.04007
199	**EFTU**	P49411	Elongation factor Tu, mitochondrial	3.64175	3.42478
200	**RS3**	P23396	40S ribosomal protein S3	2.61813	3.38603
201	**MTREX**	P42285	Exosome RNA helicase MTR4	2.57741	2.61761
202	**RS26**	P62854	40S ribosomal protein S26	2.66498	3.15958
203	**RS16**	P62249	40S ribosomal protein S16	2.87042	5.66172
204	**A0A087WZT3**	A0A087WZT3	BolA-like protein 2	2.62918	3.9663
205	**LANC1**	O43813	Glutathione S-transferase LANCL1	4.28469	3.09074
206	**CND3**	Q9BPX3	Condensin complex subunit 3	2.9969	2.66232
207	**AIMP2**	Q13155	Aminoacyl tRNA synthase complex-interacting multifunctional protein 2	3.83739	3.0309
208	**G3V325**	G3V325	ATP5MF-PTCD1 readthrough	4.35305	4.77429
209	**TGM2**	P21980	Protein-glutamine gamma-glutamyltransferase 2	3.47902	2.55024
210	**RS18**	P62269	40S ribosomal protein S18	3.20805	4.17992
211	**RS14**	P62263	40S ribosomal protein S14	2.67833	4.25944
212	**C9JXB8**	C9JXB8	60S ribosomal protein L24	2.98512	4.69503
213	**MGST2**	Q99735	Microsomal glutathione S-transferase 2	2.68604	3.32233
214	**RL13A**	P40429	60S ribosomal protein L13a	2.87139	4.09016
215	**SNR40**	Q96DI7	U5 small nuclear ribonucleoprotein 40 kDa protein	2.83902	2.94416
216	**SRP54**	P61011	Signal recognition particle 54 kDa protein	3.37647	3.10435
217	**CUL4A**	Q13619	Cullin-4A	5.00644	2.58406
218	**UCK2**	Q9BZX2	Uridine-cytidine kinase 2	2.99612	2.78459
219	**K7EP65**	K7EP65	60S ribosomal protein L22	3.68703	6.73393
220	**RS19**	P39019	40S ribosomal protein S19	3.54415	7.35693
221	**TSYL1**	Q9H0U9	Testis-specific Y-encoded-like protein 1	2.66813	3.09096
222	**E9PJD9**	E9PJD9	60S ribosomal protein L27a	3.04224	5.20122
223	**VPS45**	Q9NRW7	Vacuolar protein sorting-associated protein 45	2.73297	4.2448
224	**M0R3D6**	M0R3D6	60S ribosomal protein L18a	3.29172	4.15871
225	**RS6**	P62753	40S ribosomal protein S6	2.85538	3.91982
226	**H7C2W9**	H7C2W9	60S ribosomal protein L31	2.97225	6.33005
227	**NUCL**	P19338	Nucleolin	3.49248	4.59942
228	**A0A0D9SG12**	A0A0D9SG12	ATP-dependent RNA helicase DDX3X	2.69323	4.56215
229	**CDC73**	Q6P1J9	Parafibromin	3.60285	2.99202
230	**APT**	P07741	Adenine phosphoribosyltransferase	3.34193	2.61598
231	**E7ENU7**	E7ENU7	Ribosomal protein L15	3.97322	2.8185
232	**G3BP1**	Q13283	Ras GTPase-activating protein-binding protein 1	3.04396	3.61278
233	**K7ERT8**	K7ERT8	60S ribosomal protein L23a	4.22784	5.36401
234	**J3QQ67**	J3QQ67	60S ribosomal protein L18	2.66443	2.95231
235	**XRCC6**	P12956	X-ray repair cross-complementing protein 6	3.48879	3.75823
236	**M0R0R2**	M0R0R2	40S ribosomal protein S5	4.26617	5.14718
237	**RFC5**	P40937	Replication factor C subunit 5	4.36703	3.35394

238	**F8W727**	F8W727	60S ribosomal protein L32	3.74762	5.39745
239	**MD2L1**	Q13257	Mitotic spindle assembly checkpoint protein MAD2A	2.92545	2.98028
240	**D6RAN4**	D6RAN4	60S ribosomal protein L9	3.31616	4.19725
241	**RL4**	P36578	60S ribosomal protein L4	2.85467	3.7287
242	**RL36**	Q9Y3U8	60S ribosomal protein L36	4.53022	5.5977
243	**SSBP**	Q04837	Single-stranded DNA-binding protein, mitochondrial	5.65442	4.22935
244	**RS10**	P46783	40S ribosomal protein S10	3.25188	3.02068
245	**C9JZI1**	C9JZI1	Replication factor C subunit 4	2.62627	3.47232
246	**XRCC5**	P13010	X-ray repair cross-complementing protein 5	2.60763	3.48406
247	**AMPN**	P15144	Aminopeptidase N	3.75088	3.23543
248	**GTF2I**	P78347	General transcription factor II-I	2.60151	3.52247
249	**RS7**	P62081	40S ribosomal protein S7	3.97012	3.42622
250	**C9JQV0**	C9JQV0	Uncharacterized protein C7orf50	2.86837	2.93894
251	**ACOX3**	O15254	Peroxisomal acyl-coenzyme A oxidase 3	2.7579	4.58583

Table 9: Top 5 networks identified from IPA analysis in WT-tau expressing cells

ID	Associated network functions	Score
1	RNA damage and repair, protein synthesis, cancer	61
2	DNA replication, recombination, and repair, cellular assembly and organization, cell morphology	41
3	Cell cycle, cellular function and maintenance, molecular transport	28
4	Cellular movement, haematological system development and function, immune cell trafficking	21
5	Organismal injury and abnormalities, cellular movement, skeletal and muscular system development and function	17

Table 10: Top 5 networks identified from IPA analysis in P301L-tau expressing cells

ID	Associated network functions	Score
1	RNA damage and repair, protein synthesis, cancer	55
2	Cellular assembly and organization, cellular compromise, cellular function and maintenance	52
3	Nucleic acid metabolism, small molecule biochemistry, digestive system development and function	27
4	Cellular compromise, developmental disorder, endocrine system disorders	22
5	Developmental disorder, endocrine system disorders, organ morphology	11

Table 11: Significantly enriched proteins in all comparisons (rpAD vs Cont, spAD vs Cont, sCJD vs Cont, rpAD vs spAD, spAD vs sCJD, rpAD vs sCJD) are listed

No.	Proteins	Accession No.	rpAD/cont	spAD/cont	sCJD/cont	rpAD/spAD	spAD/sCJD	rpAD/sCJD
1	Clathrin coat assembly protein AP180	AP180	+	+	+			
2	60 kDa heat shock protein, mitochondrial	CH60	+	+	+			
3	N(G),N(G)-dimethylarginine dimethylaminohydrolase 1	DDAH1		+	+		+	+
4	Filaggrin-2	FILA2	+	+	+			
5	Fascin	FSCN1	+	+	+		+	
6	Guanine nucleotide-binding protein G(o) subunit alpha	GNAO		+	+			
7	Lysozyme C	LYSC	+	+	+			
8	Microtubule-associated protein 1A	MAP1A	+	+	+			
9	Protein kinase C and casein kinase substrate in neurons protein 1	PACN1	+	+	+			
10	Ras-related protein Rab-1A	RAB1A	+	+	+		+	
11	Rho-related GTP-binding protein RhoB	RHOB		+	+	+		
12	Ribonuclease inhibitor	RINI	+	+	+	+	+	
13	Prosaposin	SAP		+	+	+	+	
14	Secernin-1	SCRN1		+	+			
15	Septin-7	Septin-7	+	+	+	+		
16	Protein-glutamine gamma-glutamyltransferase E	TGM3	+	+	+			
17	14-3-3 protein sigma	1433S			+			
18	3-hydroxyisobutyrate dehydrogenase, mitochondrial	3HIDH			+		+	+
19	6-phosphogluconate dehydrogenase	6PGD	+		+	+	+	
20	Alpha-1-acid glycoprotein 1	A1AG1			+		+	
21	Alpha-1-antitrypsin	A1AT	+		+	+	+	
22	Alpha-2-macroglobulin	A2MG			+		+	+
23	Alpha-1-antichymotrypsin	AACT			+		+	
24	Aspartate aminotransferase, cytoplasmic	AATC	+		+	+	+	
25	Aconitate hydratase, mitochondrial	ACON	+		+		+	
26	Alpha-actinin-1	ACTN1			+		+	+
27	Acylphosphatase-2	ACYP2	+		+	+	+	
28	Beta-adducin	ADDB			+		+	+
29	Alcohol dehydrogenase class-3	ADHX			+		+	+
30	Retinal dehydrogenase 1	AL1A1			+		+	
31	Delta-1-pyrroline-5-carboxylate dehydrogenase, mitochondrial	AL4A1			+		+	
32	Alpha-aminoadipic semialdehyde dehydrogenase	AL7A1	+		+		+	+

33	4-trimethylaminobutyraldehyde dehydrogenase	AL9A1			+		+	
34	Aldehyde dehydrogenase, mitochondrial	ALDH2	+		+	+	+	+
35	Fructose-bisphosphate aldolase A	ALDOA			+		+	
36	Fructose-bisphosphate aldolase C	ALDOC			+		+	
37	Aldose reductase	ALDR			+		+	
38	Cytosol aminopeptidase	AMPL			+		+	
39	Annexin A2	ANXA2			+		+	
40	Annexin A5	ANXA5			+	+	+	
41	Annexin A6	ANXA6	+		+		+	
42	Annexin A7	ANXA7			+			
43	AP-2 complex subunit beta	AP2B1			+		+	+
44	Apolipoprotein E	APOE			+			
45	ADP-ribosylation factor 1	ARF1			+		+	
46	Aflatoxin B1 aldehyde reductase member 2	ARK72			+		+	
47	Actin-related protein 2	ARP2			+		+	+
48	Sodium/potassium-transporting ATPase subunit beta-1	AT1B1			+		+	
49	ATP synthase subunit beta, mitochondrial	ATPB			+		+	
50	Cytosolic acyl coenzyme A thioester hydrolase	BACH			+		+	+
51	Myc box-dependent-interacting protein 1	BIN1	+		+			
52	Carbonic anhydrase 1	CAH1			+		+	
53	Carbonic anhydrase 2	CAH2			+	+	+	
54	Cullin-associated NEDD8-dissociated protein 1	CAND1			+		+	
55	Adenylyl cyclase-associated protein 1	CAP1			+		+	
56	Cathepsin D	CATD	+		+		+	
57	F-actin-capping protein subunit alpha-2	CAZA2			+		+	
58	Carbonyl reductase [NADPH] 1	CBR1			+		+	
59	Cell division control protein 42 homolog	CDC42			+			
60	10 kDa heat shock protein, mitochondrial	CH10	+		+		+	
61	Citrate synthase, mitochondrial	CISY	+		+		+	
62	Cytosolic non-specific dipeptidase	CNDP2			+		+	
63	CB1 cannabinoid receptor-interacting protein 1	CNRP1			+		+	
64	Contactin-1	CNTN1			+			
65	Cofilin-1	COF1			+	+	+	
66	Copine-5	CPNE5			+		+	
67	Cytochrome c	CYC	+		+	+	+	
68	2,4-dienoyl-CoA reductase, mitochondrial	DECR			+		+	

69	Glutamate dehydrogenase 1, mitochondrial	DHE3	+		+		+	+
70	Dihydropteridine reductase	DHPR			+			
71	Dihydrolipoyl dehydrogenase, mitochondrial	DLDH			+		+	+
72	D-dopachrome decarboxylase	DOPD			+		+	
73	Dihydropyrimidinase-related protein 1	DPYL1			+			
74	Dihydropyrimidinase-related protein 3	DPYL3			+		+	
75	Dihydropyrimidinase-related protein 5	DPYL5			+		+	
76	Dynein light chain 2, cytoplasmic	DYL2			+		+	
77	Delta(3,5)-Delta(2,4)-dienoyl-CoA isomerase, mitochondrial	ECH1			+			
78	Enoyl-CoA hydratase, mitochondrial	ECHM	+		+	+	+	
79	Enoyl-CoA delta isomerase 1, mitochondrial	ECI1			+		+	
80	Elongation factor 1-gamma	EF1G			+		+	
81	Alpha-enolase	ENOA			+		+	
82	Enolase-phosphatase E1	ENOPH			+		+	
83	S-formylglutathione hydrolase	ESTD			+		+	
84	Electron transfer flavoprotein subunit alpha, mitochondrial	ETFA			+		+	
85	Ezrin	EZRI			+		+	+
86	Hsc70-interacting protein	F10A1	+		+		+	
87	Protein FAM49B	FA49B			+	+	+	
88	Fatty acid-binding protein, heart	FABPH			+		+	
89	Acylpyruvase FAHD1, mitochondrial	FAHD1			+		+	
90	Fibrinogen beta chain	FIBB			+		+	
91	Fibrinogen gamma chain	FIBG			+		+	
92	Filaggrin	FILA			+			
93	Ferritin heavy chain	FRIH			+	+	+	
94	Ferritin light chain	FRIL			+		+	
95	Fumarate hydratase, mitochondrial	FUMH	+		+			
96	Glucose-6-phosphate isomerase	G6PI			+		+	
97	4-aminobutyrate aminotransferase, mitochondrial	GABT	+		+		+	
98	Guanine nucleotide-binding protein G(I)/G(S)/G(T) subunit beta-1	GBB1			+		+	
99	Rab GDP dissociation inhibitor alpha	GDIA	+		+		+	
100	Rab GDP dissociation inhibitor beta	GDIB	+		+	+	+	
101	Rho GDP-dissociation inhibitor 1	GDIR1			+		+	
102	Gelsolin	GELS			+		+	

103	Gamma-glutamylcyclotransferase	GGCT			+		+	
104	Glutamine synthetase	GLNA			+		+	+
105	Glyoxalase domain-containing protein 4	GLOD4			+		+	
106	Neuronal membrane glycoprotein M6-a	GPM6A	+		+		+	
107	Stress-70 protein, mitochondrial	GRP75			+		+	
108	Glutathione S-transferase Mu 1	GSTM1			+		+	+
109	Glutathione S-transferase Mu 2	GSTM2			+		+	+
110	Glutathione S-transferase Mu 3	GSTM3			+		+	+
111	Glutathione S-transferase omega-1	GSTO1			+		+	+
112	Glutathione S-transferase P	GSTP1			+		+	
113	Guanine deaminase	GUAD			+		+	+
114	Haloacid dehalogenase-like hydrolase domain-containing protein 2	HDHD2			+		+	
115	Heterogeneous nuclear ribonucleoprotein D0	HNRPD			+			
116	Heterogeneous nuclear ribonucleoprotein K	HNRPK			+		+	
117	Hyaluronan and proteoglycan link protein 1	HPLN1			+		+	
118	Hypoxanthine-guanine phosphoribosyltransferase	HPRT	+		+	+	+	+
119	Haptoglobin	HPT			+		+	+
120	Heat shock protein 105 kDa	HS105			+		+	+
121	Heat shock protein HSP 90-alpha	HS90A			+		+	
122	Heat shock protein HSP 90-beta	HS90B			+		+	
123	Heat shock 70 kDa protein 4	HSP74			+		+	
124	Hexokinase-1	HXK1			+		+	
125	Immunoglobulin gamma-1 heavy chain	IGG1	+		+	+	+	
126	Immunoglobulin heavy constant alpha 1	IGHA1			+		+	
127	Immunoglobulin heavy constant gamma 2	IGHG2			+		+	
128	Immunoglobulin heavy constant gamma 3	IGHG3	+		+	+	+	
129	Immunoglobulin heavy constant gamma 4	IGHG4	+		+	+	+	
130	Immunoglobulin kappa constant	IGKC	+		+	+	+	
131	Inositol monophosphatase 1	IMPA1			+		+	
132	GTP:AMP phosphotransferase AK3, mitochondrial	KAD3			+		+	
133	Pyruvate kinase PKM	KPYM			+		+	
134	LanC-like protein 1	LANC1	+		+	+	+	
135	Galectin-1	LEG1	+		+			
136	Malate dehydrogenase, cytoplasmic	MDHC			+		+	

137	Malate dehydrogenase, mitochondrial	MDHM			+		+	
138	Mitogen-activated protein kinase 1	MK01			+			
139	Methylmalonate-semialdehyde dehydrogenase [acylating], mitochondrial	MMSA			+		+	
140	Moesin	MOES			+		+	+
141	NAD kinase 2, mitochondrial	NAKD2			+		+	
142	Neural cell adhesion molecule 1	NCAM1			+			
143	Neurocan core protein	NCAN	+		+	+	+	
144	Neurochondrin	NCDN			+		+	+
145	Neurofascin	NFASC			+		+	
146	NAD(P)H-hydrate epimerase	NNRE			+		+	
147	Neuronal cell adhesion molecule	NRCAM			+		+	
148	Protein/nucleic acid deglycase DJ-1	PARK7			+	+	+	
149	Poly(rC)-binding protein 2	PCBP2			+		+	
150	Protein disulfide-isomerase	PDIA1			+		+	+
151	Protein disulfide-isomerase A6	PDIA6			+		+	
152	ATP-dependent 6-phosphofructokinase, muscle type	PFKAM			+		+	+
153	Phosphoglycerate mutase 1	PGAM1			+			
154	Brevican core protein	PGCB	+		+		+	
155	Phosphoglycerate kinase 1	PGK1			+		+	
156	Phosphoglucomutase-1	PGM1			+		+	+
157	Protein-L-isoaspartate(D-aspartate) O-methyltransferase	PIMT			+		+	+
158	Junction plakoglobin	PLAK			+			
159	Pyridoxal phosphate phosphatase	PLPP			+			
160	Protein phosphatase 1 regulatory subunit 7	PP1R7			+		+	
161	Serine/threonine-protein phosphatase 2A catalytic subunit alpha isoform	PP2AA			+		+	
162	Serine/threonine-protein phosphatase 2B catalytic subunit alpha isoform	PP2BA			+		+	+
163	Serine/threonine-protein phosphatase 5	PPP5			+		+	+
164	Palmitoyl-protein thioesterase 1	PPT1	+		+			
165	Peroxiredoxin-1	PRDX1			+		+	
166	Peroxiredoxin-2	PRDX2			+		+	
167	Peroxiredoxin-5, mitochondrial	PRDX5			+		+	
168	Peroxiredoxin-6	PRDX6			+	+	+	
169	Profilin-1	PROF1			+	+	+	
170	Profilin-2	PROF2			+		+	+

171	Puromycin-sensitive aminopeptidase	PSA			+		+	+
172	Proteasome subunit alpha type-4	PSA4			+		+	+
173	Proteasome subunit alpha type-6	PSA6			+		+	
174	Proteasome subunit alpha type-7	PSA7			+		+	+
175	Proteasome subunit beta type-4	PSB4			+		+	
176	Proteasome subunit beta type-6	PSB6			+		+	
177	Prostaglandin-H2 D-isomerase	PTGDS			+		+	+
178	Receptor-type tyrosine-protein phosphatase zeta	PTPRZ			+		+	+
179	Multifunctional protein ADE2	PUR6			+		+	+
180	Quinone oxidoreductase	QOR			+		+	
181	Ras-related protein Rab-2A	RAB2A			+			
182	Ras-related protein Rab-5A	RAB5A			+		+	+
183	Ras-related protein Rab-5B	RAB5B			+			
184	Ras-related protein Rab-5C	RAB5C			+		+	
185	Ras-related protein Rab-7a	RAB7A			+		+	+
186	Ras-related C3 botulinum toxin substrate 1	RAC1			+		+	+
187	Radixin	RADI			+		+	+
188	GTP-binding nuclear protein Ran	RAN			+		+	
189	Transforming protein RhoA	RHOA			+			
190	2-iminobutanoate/2-iminopropanoate deaminase	RIDA			+	+	+	
191	40S ribosomal protein S16	RS16			+			
192	Reticulon-1	RTN1			+			
193	U2 small nuclear ribonucleoprotein B"	RU2B			+			
194	Adenosylhomocysteinase	SAHH			+		+	+
195	S-adenosylhomocysteine hydrolase-like protein 1	SAHH2			+		+	+
196	Selenium-binding protein 1	SBP1			+	+	+	
197	Septin-11	Septin-11	+		+		+	
198	Septin-2	Septin-2	+		+	+	+	
199	Phosphoserine aminotransferase	SERC			+		+	
200	Endophilin-A1	SH3G2			+		+	+
201	Superoxide dismutase [Cu-Zn]	SODC			+			
202	Superoxide dismutase [Mn], mitochondrial	SODM	+		+	+	+	
203	Serpin B3	SPB3			+			
204	Spectrin beta chain, non-erythrocytic 1	SPTB2			+		+	+
205	Spectrin alpha chain, non-erythrocytic 1	SPTN1			+		+	
206	Single-stranded DNA-binding protein, mitochondrial	SSBP			+			

207	Succinate-semialdehyde dehydrogenase, mitochondrial	SSDH			+		+	+
208	Stress-induced-phosphoprotein 1	STIP1			+		+	
209	Syntaxin-1B	STX1B	+		+			
210	Synapsin-2	SYN2			+		+	
211	Transgelin-3	TAGL3	+		+	+	+	
212	Transaldolase	TALDO			+		+	
213	T-complex protein 1 subunit theta	TCPQ			+		+	
214	Tenascin-R	TENR			+			
215	Transitional endoplasmic reticulum ATPase	TERA	+		+			
216	Acetyl-CoA acetyltransferase, mitochondrial	THIL	+		+			
217	Thy-1 membrane glycoprotein	THY1			+		+	+
218	Transketolase	TKT	+		+	+	+	
219	Triosephosphate isomerase	TPIS			+	+	+	
220	Serotransferrin	TRFE			+		+	
221	Ubiquitin-conjugating enzyme E2 variant 2	UB2V2			+		+	
222	Polyubiquitin-B	UBB			+	+	+	
223	UTP--glucose-1-phosphate uridylyltransferase	UGPA			+		+	
224	V-type proton ATPase catalytic subunit A	VATA			+		+	+
225	V-type proton ATPase subunit B, brain isoform	VATB2			+		+	
226	V-type proton ATPase subunit E 1	VATE1			+		+	+
227	V-type proton ATPase subunit H	VATH			+			
228	Visinin-like protein 1	VISL1			+		+	
229	14-3-3 protein epsilon	1433E		+		+	+	
230	14-3-3 protein eta	1433F		+			+	
231	Serum albumin	ALBU		+			+	
232	Plasma membrane calcium-transporting ATPase 2	AT2B2	+	+			+	+
233	Cell adhesion molecule 2	CADM2		+		+	+	
234	Cell adhesion molecule 3	CADM3		+			+	
235	Cell cycle exit and neuronal differentiation protein 1	CEND	+	+			+	+
236	Hydroxyacylglutathione hydrolase, mitochondrial	GLO2		+		+		
237	Microtubule-associated protein 1B	MAP1B		+				
238	MARCKS-related protein	MRP	+	+			+	+
239	Metallothionein-2	MT2	+	+			+	+
240	Metallothionein-3	MT3		+			+	+
241	Neurogranin	NEUG	+	+			+	+
242	Neuroplastin	NPTN	+	+			+	+
243	Oxidation resistance protein 1	OXR1		+		+		
244	ProSAAS	PCSK1		+			+	

245	Synaptojanin-1	SYNJ1		+				
246	Alpha-synuclein	SYUA		+			+	
247	Ubiquitin-like modifier-activating enzyme 1	UBA1		+				
248	Aspartate aminotransferase, mitochondrial	AATM				+	+	
249	L-aminoadipate-semialdehyde dehydrogenase-phosphopantetheinyl transferase	ADPPT					+	
250	ADP/ATP translocase 1	ADT1				+		+
251	Amine oxidase [flavin-containing] A	AOFA						+
252	Amine oxidase [flavin-containing] B	AOFB	+			+		+
253	Actin-related protein 2/3 complex subunit 1A	ARC1A					+	
254	Acid ceramidase	ASAH1					+	
255	Sodium/potassium-transporting ATPase subunit alpha-3	AT1A3	+					
256	Sodium/potassium-transporting ATPase subunit beta-2	AT1B2						+
257	ATP synthase F(0) complex subunit B1, mitochondrial	AT5F1						+
258	ATP synthase subunit d, mitochondrial	ATP5H	+			+		+
259	ATP synthase-coupling factor 6, mitochondrial	ATP5J	+			+		+
260	ATP synthase subunit g, mitochondrial	ATP5L	+			+		+
261	ATP synthase subunit O, mitochondrial	ATPO				+		+
262	Biliverdin reductase A	BIEA					+	
263	Calmodulin-1	CALM1				+		+
264	Catalase	CATA					+	
265	CD81 antigen	CD81	+			+		+
266	CDGSH iron-sulfur domain-containing protein 1	CISD1	+			+		+
267	Clusterin	CLUS					+	
268	Cofilin-2	COF2				+	+	
269	Lambda-crystallin homolog	CRYL1					+	
270	Ketimine reductase mu-crystallin	CRYM					+	
271	Versican core protein	CSPG2					+	
272	Cysteine and glycine-rich protein 1	CSRP1					+	
273	Excitatory amino acid transporter 1	EAA1				+	+	+
274	Excitatory amino acid transporter 2	EAA2	+					
275	Electron transfer flavoprotein subunit beta	ETFB					+	
276	Fatty acid-binding protein, epidermal	FABP5					+	
277	Growth arrest-specific protein 7	GAS7					+	

278	Glial fibrillary acidic protein	GFAP					+	
279	78 kDa glucose-regulated protein	GRP78				+	+	
280	Very-long-chain (3R)-3-hydroxyacyl-CoA dehydratase 3	HACD3						+
281	Hepatocyte cell adhesion molecule	HECAM	+			+		+
282	Delta-aminolevulinic acid dehydratase	HEM2					+	
283	Heat shock 70 kDa protein 1A	HS71A					+	
284	Heat shock-related 70 kDa protein 2	HSP72					+	
285	Heat shock cognate 71 kDa protein	HSP7C					+	
286	Isocitrate dehydrogenase [NADP] cytoplasmic	IDHC					+	
287	Immunoglobulin lambda constant 2	IGLC2				+	+	
288	Immunoglobulin superfamily member 8	IGSF8	+					
289	LIM and SH3 domain protein 1	LASP1					+	
290	Myelin-associated glycoprotein	MAG					+	
291	Microtubule-associated protein 6	MAP6	+				+	+
292	Myristoylated alanine-rich C-kinase substrate	MARCS				+		+
293	Myelin basic protein	MBP					+	
294	Macrophage migration inhibitory factor	MIF					+	
295	Myelin-oligodendrocyte glycoprotein	MOG				+		+
296	Metallothionein-1F	MT1F					+	
297	Myotrophin	MTPN				+		
298	Myelin proteolipid protein	MYPR				+		+
299	Protein NDRG1	NDRG1	+					
300	Protein NDRG2	NDRG2	+					
301	Dihydrolipoyllysine-residue succinyltransferase component of 2-oxoglutarate dehydrogenase complex, mitochondrial	ODO2					+	
302	Platelet-activating factor acetylhydrolase IB subunit beta	PA1B2					+	
303	Programmed cell death 6-interacting protein	PDC6I					+	
304	Phosphatidylethanolamine-binding protein 1	PEBP1				+	+	
305	Phosphatidylinositol-binding clathrin assembly protein	PICAL				+		
306	Phosphatidylinositol transfer protein alpha isoform	PIPNA					+	
307	Plectin	PLEC					+	
308	Serine/threonine-protein ph	PP1G					+	

309	Peptidyl-prolyl cis-trans isomerase A	PPIA					+	
310	Peptidyl-prolyl cis-trans isomerase B	PPIB					+	
311	PRA1 family protein 3	PRAF3						+
312	Proline-rich transmembrane protein 2	PRRT2	+			+		+
313	Proteasome subunit alpha type-3	PSA3					+	
314	Prothymosin alpha	PTMA						+
315	Glycogen phosphorylase, brain form	PYGB					+	
316	Cytochrome b-c1 complex subunit 1, mitochondrial	QCR1	+			+		+
317	UV excision repair protein RAD23 homolog B	RD23B						+
318	Protein S100-B	S100B	+			+		+
319	Protein S100-A1	S10A1	+			+		
320	Neutral amino acid transporter A	SATT						+
321	Succinate dehydrogenase [ubiquinone] flavoprotein subunit, mitochondrial	SDHA	+			+		+
322	SH3 domain-binding glutamic acid-rich-like protein	SH3L1	+			+		
323	Tyrosine-protein phosphatase non-receptor type substrate 1	SHPS1	+					+
324	Beta-soluble NSF attachment protein	SNAB	+					
325	Synaptogyrin-3	SNG3	+					
326	Serpin B6	SPB6					+	
327	Syntaxin-12	STX12					+	
328	Synaptotagmin-1	SYT1					+	
329	Transcription elongation factor A protein-like 3	TCAL3					+	
330	Toll-interacting protein	TOLIP					+	
331	Thioredoxin-like protein 1	TXNL1					+	
332	Thymosin beta-10	TYB10					+	
333	Thymosin beta-4	TYB4					+	
334	Ubiquitin-conjugating enzyme E2 L3	UB2L3	+			+		
335	Ubiquitin-conjugating enzyme E2 N	UBE2N				+	+	
336	Up-regulated during skeletal muscle growth protein 5	USMG5	+			+		+
337	Vesicle-associated membrane protein 2	VAMP2	+			+		
338	Voltage-dependent anion-selective channel protein 2	VDAC2						+

Annexure

Uncropped blots

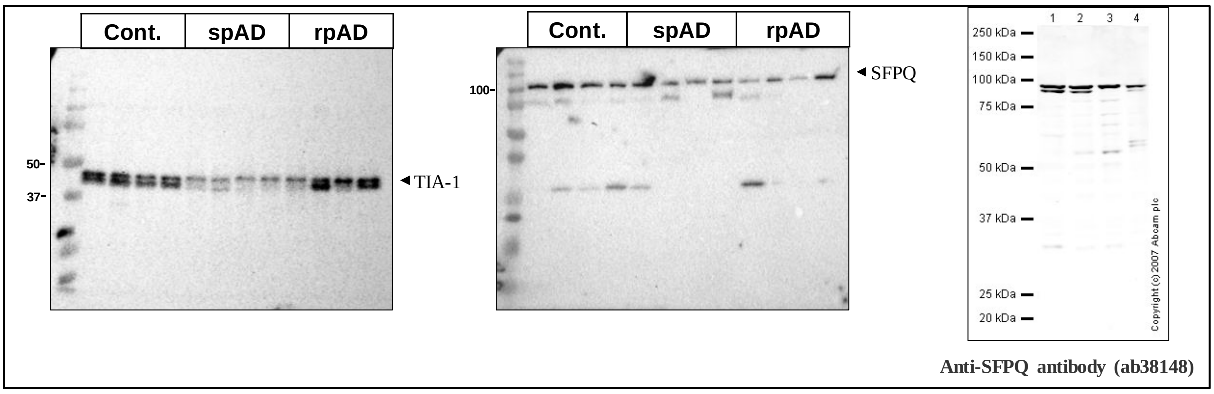

Fig. 5a uncropped blots: Additional bands for SFPQ: We are unsure as to the identity of these extra bands. For reference abcam image has been displayed showing additional bands.

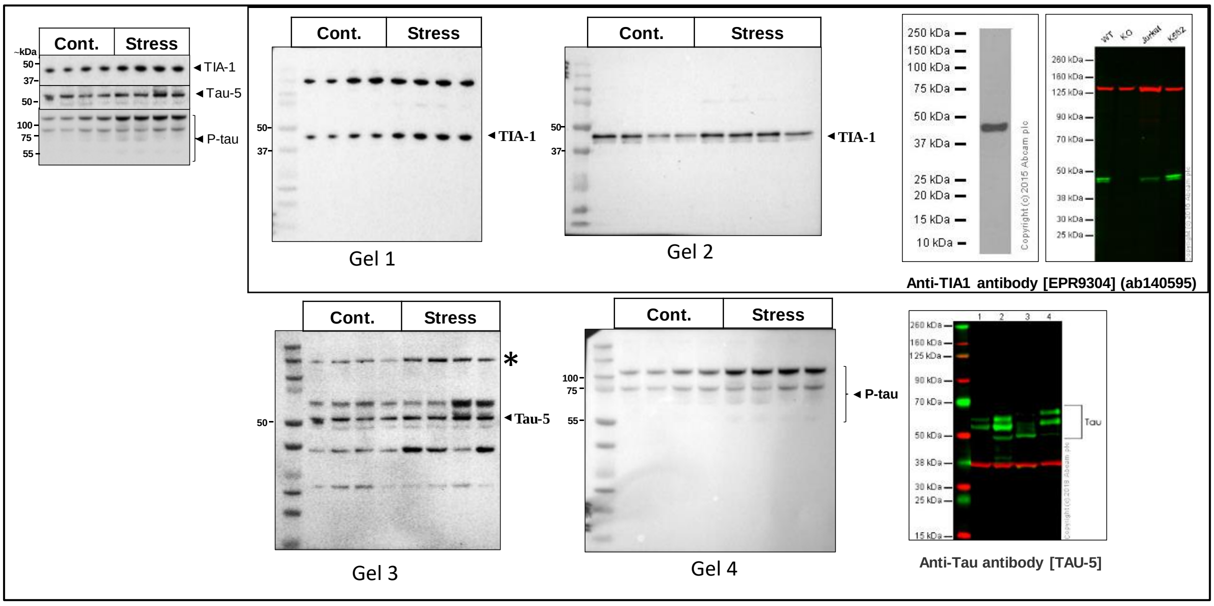

Fig. 7f uncropped blots: For TIA-1, two banding patterns have been reported, in 12 % gels, a double band pattern and in gradient gels (4-12 %), a single band pattern. We have run TIA-1 in both gradient (**Gel 1**, representative blot in the Manuscript) and in 12% gel (**Gel 2**) for confirmation. Abcam images for the antibody are also displayed on the right side. **Gel 3:** Tau-5 antibody, Tau-5 detects total tau both phosphorylated and unphosphorylated form of tau protein. Different isoforms of tau are reported between 75-50 kDa and tau cleavage products below 50 kDa. Star ***** represents high molecular weight phosphorylated oligomeric species of tau as Tau-5 also detects phosphorylated tau.

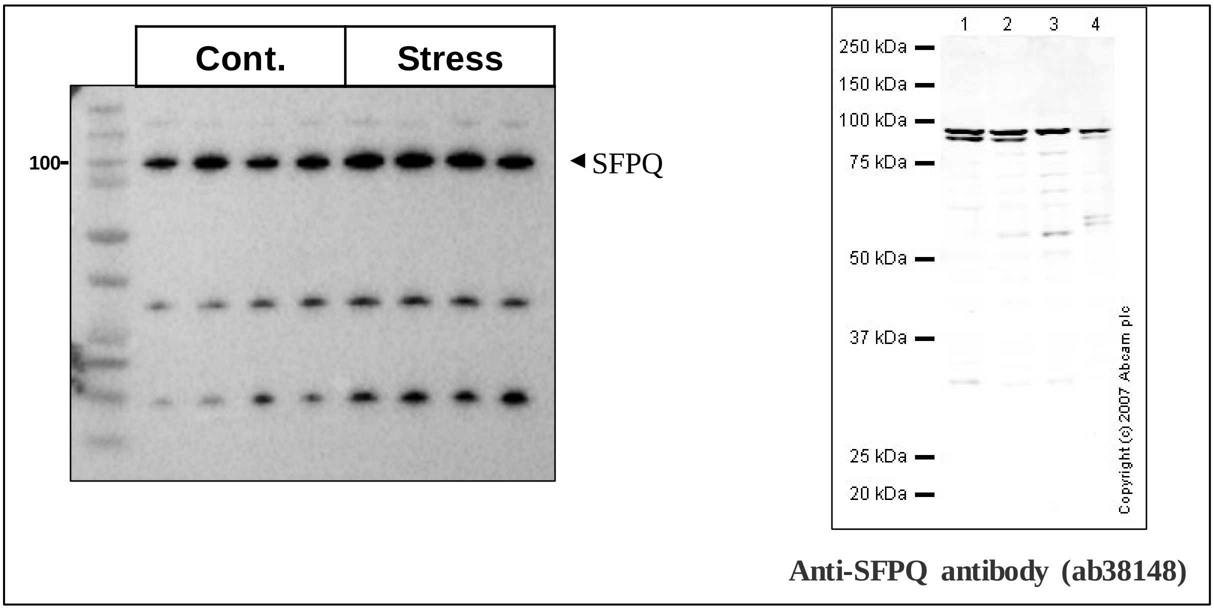

Fig. 8d uncropped blots: **Additional bands** for SFPQ: We are unsure as to the identity of these extra bands. For reference abcam image has been attached.

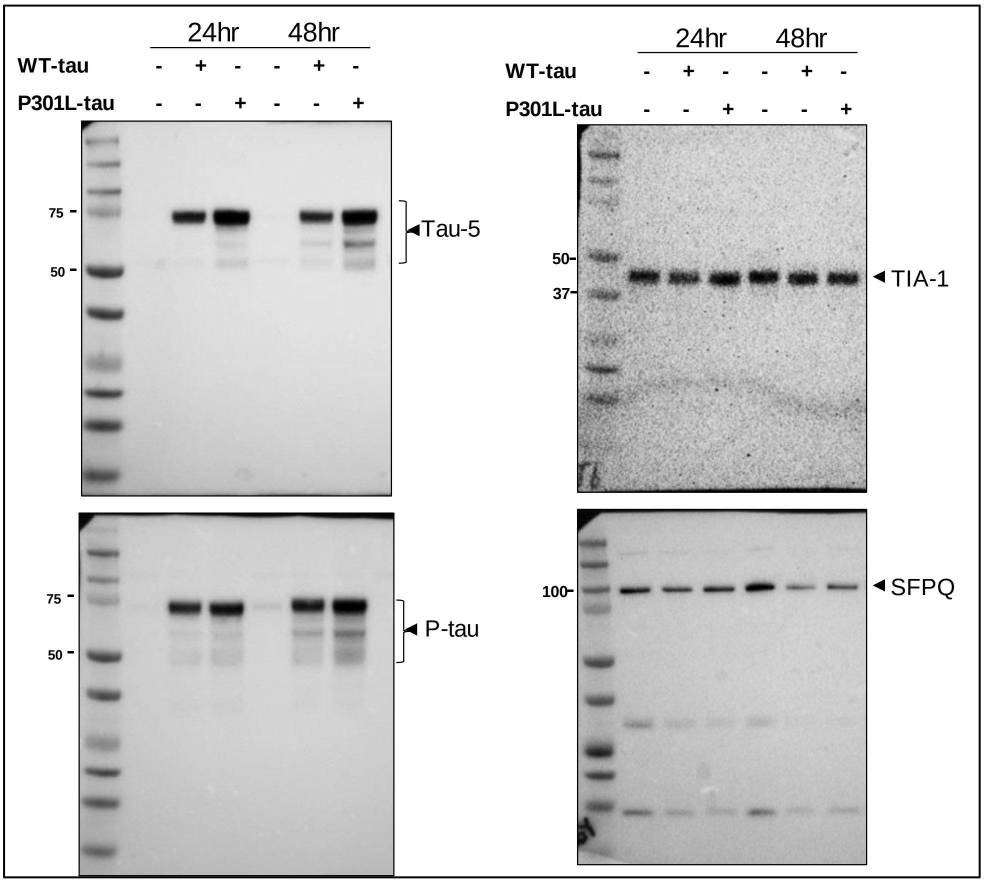

Fig. 9 uncropped blots: Representative immunoblot images for tau, p-tau, SFPQ and TIA-1 after transient transfection of WT-tau or P301L-tau (0N4R) in HeLa cells. All bands for tau and its phosphorylated form were cut and identified by Mass spectrometry to confirm the identification of Tau after Transfection.

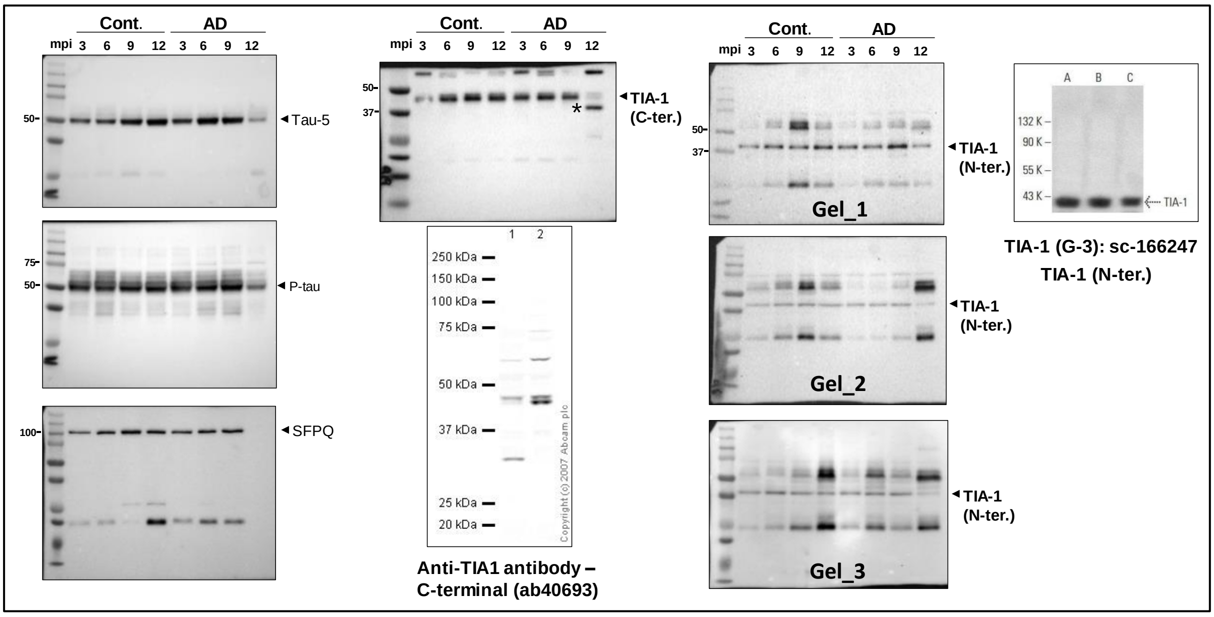

Fig. 11a uncropped blots: Representative full blots and reference blots of corresponding antibodies are displayed. For TIA-1 (N-terminal), 3 different gels have been displayed. We are unsure of these additional bands which are appearing with TIA-1 (N-terminal).

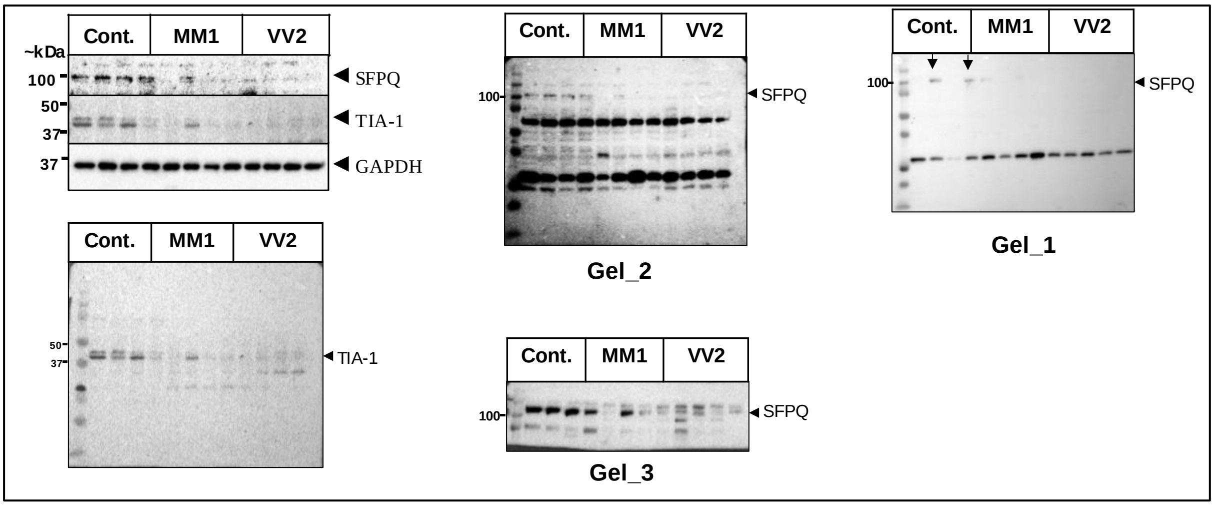

Supplementary Fig. 2a uncropped blot: Representative full blots, For TIA-1 a typical double band pattern was observed. For SFPQ, **Gel_1:** As the expression of SFPQ was very low in particularly MM1 and VV2, and bands were visible only in controls (**refer to arrows in Gel-1**). Another gel is displayed (**Gel-2**), where concentration of secondary antibody was increased to enhance detection of SFPQ, which has resulted in high background bands. A third gel (**Gel-3**) is displayed as well for reference, where upper part of the membrane was probed with SFPQ to increase specificity, showing a good expression of SFPQ.
